# Lie Group Cohomology and (Multi)Symplectic Integrators: New Geometric Tools for Lie Group Machine Learning Based on Souriau Geometric Statistical Mechanics

**DOI:** 10.3390/e22050498

**Published:** 2020-04-25

**Authors:** Frédéric Barbaresco, François Gay-Balmaz

**Affiliations:** 1 Key Technology Domain PCC (Processing, Control & Cognition) Representative, Thales Land & Air Systems, Voie Pierre-Gilles de Gennes, F91470 Limours, France; frederic.barbaresco@thalesgroup.com; 2 Centre National de la Recherche Scientifique (CNRS), Le Laboratoire de Météorologie Dynamique (LMD), Ecole Normale Supérieure, 75005 Paris, France

**Keywords:** momentum maps, cocycles, Lie group actions, coadjoint orbits, variational integrators, (multi)symplectic integrators, fisher metric, Gibbs probability density, entropy, Lie group machine learning, Casimir functions

## Abstract

In this paper, we describe and exploit a geometric framework for Gibbs probability densities and the associated concepts in statistical mechanics, which unifies several earlier works on the subject, including Souriau’s symplectic model of statistical mechanics, its polysymplectic extension, Koszul model, and approaches developed in quantum information geometry. We emphasize the role of equivariance with respect to Lie group actions and the role of several concepts from geometric mechanics, such as momentum maps, Casimir functions, coadjoint orbits, and Lie-Poisson brackets with cocycles, as unifying structures appearing in various applications of this framework to information geometry and machine learning. For instance, we discuss the expression of the Fisher metric in presence of equivariance and we exploit the property of the entropy of the Souriau model as a Casimir function to apply a geometric model for energy preserving entropy production. We illustrate this framework with several examples including multivariate Gaussian probability densities, and the Bogoliubov-Kubo-Mori metric as a quantum version of the Fisher metric for quantum information on coadjoint orbits. We exploit this geometric setting and Lie group equivariance to present symplectic and multisymplectic variational Lie group integration schemes for some of the equations associated with Souriau symplectic and polysymplectic models, such as the Lie-Poisson equation with cocycle.

## 1. Introduction

A geometric theory of statistical mechanics was developed by Souriau [1], motivated by the observation that Gibbs equilibrium states do not satisfy the usual physical covariance assumptions. This geometric theory, called by him *Lie Groups Thermodynamics*, is based on a Hamiltonian action of a Lie group on a symplectic manifold, to which are associated generalized Gibbs states, indexed by a Lie algebra parameter β playing the role of a geometric (Planck) temperature. Usual Gibbs states defined from a Hamiltonian appear as special cases in which the Lie group is a one-parameter group. The generalized Gibbs states become compatible with Galileo relativity in classical mechanics and with Poincaré relativity in relativistic mechanics, and the maximum entropy principle is preserved. See [2] for an exposition of Souriau’s approach.

A natural equilibrium state is characterized by an element β of the Lie algebra of the Lie group, determining the equilibrium temperature. In this geometric setting, the logarithm of the partition function, identified with the Massieu potential Φ(β), is defined on this Lie algebra. Its derivative, called the thermodynamic heat Q(β), gives the mean value of the energy and is an element of the dual of the Lie algebra. From this, two important quantities are defined. First the *geometric heat capacity*, given by minus the derivative of *Q* and giving the Fisher metric of the generalized Gibbs probability densities, second the *entropy* defined on the dual of the Lie algebra as the Legendre transform of the Massieu potential.

This geometric setting of Souriau was exploited and developed in [3,4,5,6,7,8,9,10] towards applications in information geometry and Lie group machine learning. Different tools developed based on Souriau Lie groups thermodynamics are explored in artificial intelligence for “Supervised Machine Learning” and “Non-Supervised Machine Learning” approaches. For “Supervised Machine Learning”, neural network natural gradient from information geometry could be extended on Lie algebra based on Fisher extension with respect to Souriau covariant maximum entropy Gibbs density on coadjoint orbits. For “Non-Supervised Machine Learning”, Souriau-Fisher metric transforms problems of learning on Lie groups to more classical problems of learning on metric spaces: extension of mean/median barycenter on Lie groups by Fréchet definition of geodesic barycenter, solved by Hermann Karcher flow and by exponential map (based on Souriau algorithm for matrix characteristic polynomial computation). For “Non-Supervised Machine Learning”, extension of “mean-shift” for homogeneous symplectic manifolds and Souriau-Fisher metric space. We can also make reference to GEOMSTATS libraries [11] developing codes for machine learning on Riemannian manifolds and Lie groups.

This paper introduces basic tools to extend supervised classification, using tools from geometric statistical mechanics and information geometry, which are applied to an extension of statistical learning theory for data as elements of Lie Groups. Classically, statistical machine learning is based on convex analysis on the set of posterior probability measures with respect to Gibbs posterior measures. Lie Groups thermodynamics introduces a generalized fully covariant Gibbs density on symplectic manifolds endowed with a symplectic Lie group action admitting a momentum map. An important example is the case of coadjoint orbits endowed with the Kirilov-Kostant-Souriau symplectic forms, and its extension with non-zero cohomology. We illustrate these statistical and geometrical tools for general Lie groups, which include affine groups such as Galileo group in mechanics, and the special Euclidean groups SE(2) and SE(3) in robotics. Classically we can associate to any posterior distribution an effective generalized geometric temperature, given by an element of the dual space of the Lie algebra, relating it to the Gibbs prior distribution. Classification rules could be introduced by Gibbs measures defined on parameter sets and depending on the observed sample value. A Gibbs measure is a special kind of probability measure used in statistical mechanics to describe the state of a particle system driven by a given energy function at some given temperature. Gibbs measures will be realized as minimizers of the average loss value under entropy constraints. In this extension for Lie Groups, an important tool is the log-Laplace transform related to the Massieu Characteristic Function in Thermodynamics (a re-parameterization of the free energy by Planck temperature preserving Legendre transform with respect to Entropy). As we want to deal with Lie group data for machine learning, we will consider tools very similar to those used in statistical mechanics to describe particle systems with many degrees of freedom. Classification rules could be described by Gibbs measures defined on parameter sets and depending on the observed sample value. Comparing any posterior distribution with a Gibbs prior distribution make it possible to provide a way to build an estimator which can be proved to reach adaptively at the best possible asymptotic error rate (by temperature selection of a Gibbs posterior distribution built within a single parametric model). Estimators derived from Gibbs posteriors show excellent performance in diverse tasks, such as classification, regression and ranking. The usual recommendation is to sample from a Gibbs posterior using MCMC (Markov chain Monte Carlo). With covariant Souriau Gibbs density, it is possible to extend MCMC and Gibbs sampler approach for Lie Groups Machine Learning.

More recently, the use of perturbation techniques was proposed as an alternative to MCMC techniques for sampling. These results were extended in conditional random fields loss, proving that the maximum in expectation with low-rank perturbations, provides an upper bound on the log partition (what we call Massieu characteristic function). New lower bounds on the partition function and new unbiased sequential sampler for the Gibbs distribution based on low-rank perturbations were introduced. All these methods are based on sampling from the Gibbs distribution, upper bounding the log partition function. All these results are synthetized in [12], where a new general method is also proposed, with connections to the recently proposed Fenchel-Young losses [13], using doubly stochastic scheme for minimization of these losses, for unsupervised and supervised learning. This is a generalization to the Gibbs distribution.

Methods for learning parameters of a Gibbs disribution on data ${\left(y_{i}\right)}_{i=1,\;\ldots ,\;n}$ are based on maximization of the likelihood

$${\hat{\ell \;}}_{\;n}\left(\theta \right)=\frac{1}{n}\sum_{i=1}^{n}\log p_{\;\mathrm{Gibbs},\theta }\left(y_{i}\right)=\frac{1}{n}\sum_{i=1}^{n}\langle y_{i},\theta \rangle -\log\psi \left(\theta \right)$$

that is optimized by gradients methods using the empirical log-likelyhood, given by

$$\nabla_{\theta }{\hat{\ell \;}}_{n}={\hat{y}}_{n}-E_{\;\mathrm{Gibbs},\theta }\left(y\right).$$

For this method of moment-matching, the expectation of the Gibbs distribution is a challenge in some cases. This approach was replaced by a method called “perturb-and-MAP” to learn the parameters in this model as a proxy for log-likelihood. This minimization is equivalent to maximizing previous equation by substituting the log-partition logψ(θ) with

$$F_{\epsilon }\left(\theta \right)=E\left[F(\theta +\epsilon V)\right]=E\left[\max_{y\in C}\langle y,\theta +\epsilon V\rangle \right]$$

with a random noise vector ϵV, ϵ>0. This approach could be linked with the use of Fenchel-Young losses [13]. In the perturbed model, the Fenchel-Young loss is given by:

$$L_{\epsilon }\left(\theta ;y\right)=F_{\epsilon }\left(\theta \right)+\epsilon \Omega \left(y\right)-\langle \theta ,y\rangle =D_{\epsilon \Omega }\left(y,{\hat{y}}_{\epsilon }^{*}\left(\theta \right)\right)$$

with loss gradient $\nabla_{\theta }L_{\epsilon }\left(\theta ;y\right)=\nabla_{\theta }F_{\epsilon }\left(\theta \right)-y=y_{\epsilon }^{*}\left(\theta \right)-y$, where

$$y_{\epsilon }^{*}\left(\theta \right)=E_{p_{\theta }\left(y\right)}\left[y\right]=E\left[\arg\max_{y\in C}\langle y,\theta +\epsilon V\rangle \right]$$

and $D_{\epsilon \Omega }\left(y,{\hat{y}}_{\epsilon }^{*}\left(\theta \right)\right)$ is the Bregman divergence associated with ϵΩ. As $F_{\epsilon }$ generalizes the log-sum-exp function on the simplex, its dual Ω is a generalization of the negative entropy (which is the Fenchel dual of log-sum-exp). These connections were studied in [14].

In this paper, we describe a geometric framework for the study of Gibbs probability densities in statistical mechanics and information geometry, as well as the associated concepts of thermodynamic heat, entropy, and Fisher metric, inspired by Souriau’s symplectic model of statistical mechanics. This geometric framework unifies several earlier works on the subject, including Souriau’s symplectic model of statistical mechanics, its polysymplectic extension, Koszul model, and approaches developed in quantum information geometry. This approach helps to identify the common geometric structures appearing in various examples and provides a body of geometric tools for information geometry and Lie group machine learning. The emphasis is put on the role of the equivariance with respect to Lie group actions. For instance, we discuss the expression of the Fisher metric in presence of equivariance, we consider the associated Lie-Poisson equations with cocycle (also called affine Lie-Poisson equations) as well as their field theoretic versions, and we exploit the property of the entropy of the Souriau model as a Casimir function, to apply a geometric model for energy preserving entropy production on Lie algebras. In our developments, we make heavily use of several concepts from geometric mechanics, such as momentum maps, Casimir functions, coadjoint orbits, and Lie-Poisson brackets, as unifying concepts appearing in various applications of this framework to information geometry and machine learning. We consider in detail the Koszul model, the polysymplectic extension of the Souriau model, the case of the multivariate Gaussian probability densities, models of information geometry for quantum systems. We exploit the geometric framework to build geometric numerical integrator schemes for some of the equations associated with Souriau’s model and its polysymplectic extension. This is achieved by identifying the variational principles underlying these equations and by discretizing these principles, following the techniques of variational discretization, which result in schemes that preserve coadjoint orbits, (multi)symplectic structures, and discrete versions of Noether theorems.

The content of the paper is as follows. In Section 2.1 we present the general geometric framework for Gibbs probability densities that will be used in the paper. In particular, we review the definition of the Massieu potential, the thermodynamic heat, the entropy, the identification of the Fisher metric with the Hessian of the Massieu potential, and the maximum entropy principle. These results are independent of the existence of Lie group symmetries of the theory. The implications of such symmetries are studied in detail Section 2.2 where we present a Lie group equivariant setting that includes as special cases the Souriau model, its polysymplectic extension, and the case of multivariate Gaussian probability densities. The Souriau model is reviewed in Section 2.3 where we show that the associated entropy is a Casimir function for the Lie-Poisson bracket with cocycle and, motivated by an approach developed in quantum information geometry, we take advantage of this property to formulate a geometric model for entropy production. We also present the stochastic Hamiltonian equations associated with the Lie-Poisson bracket with cocycle. The polysymplectic model is reviewed in Section 2.4, where we show that the entropy also satisfies a natural extension of the Casimir property and we formulate a polysymplectic extension of the Lie-Poisson equations with cocycle. Finally, in Section 2.5 we give a general expression of the Fisher metric on orbits when equivariance is assumed. In Section 3 we apply the framework considered in Section 2 to various examples and identify common underlying geometric structures. We start in Section 3.1 with the case of multivariate Gaussian probability densities as an illustration of the general framework for which a cocycle is needed and which does not fall into the setting of the Souriau model. We apply Noether theorem to derive invariant quantities for geodesics of the Fisher metric. We then enlighten in Section 3.2 the strong analogies with quantum information geometry by considering Lie algebras with unitary representation and show that the Fisher metric as defined from the generalized heat capacity in Section 2.1, coincides with the Bogoliubov-Kubo-Mori metric. In this particular case the equation with Casimir dissipation/production reproduces a dissipative model used in quantum information geometry. Finally, in Section 3.3 we consider in detail the case of the Euclidean group of the plane SE(2), the associated Fisher metric, Lie-Poisson equations with cocycle and entropy production equations. In Section 4, we make use of this geometric setting to propose geometric integrators for some of the equations associated with the Souriau model and its polysymplectic extension. We first review some facts on variational integrators on Lie groups in Section 4.1 and about central extensions of Lie groups and the associated Euler-Poincaré equations in Section 4.2. This allows obtaining a variational formulation for the Lie-Poisson equations with cocycle. Based on this, we present a symplectic integrator for the Lie-Poisson equation with cocycle in Section 4.3 and a multisymplectic integrator for the Lie-Poisson field equations with cocycle in Section 4.4.

## 2. A General Framework for Lie Group Statistical Mechanics and Symmetries

### 2.1. A Class of Generalized Gibbs Probability Densities, Its Associated Entropy and Fisher Metric

In this section, we present a general framework for Gibbs probability densities in statistical mechanics and information geometry, that includes the classes considered for instance in the Koszul and Souriau models, as well as multivariate exponential families. In particular, we review the importance of the logarithm of the characteristic function, identified as the Massieu potential, from which the entropy arises as its Legendre transform and the Fisher information metric as its Hessian. We also discuss the relation of these Gibbs sates with the maximum entropy principle. While the concepts manipulated here are standard, our aim is to organize them in a general setting that is appropriate for the developments made in this paper.

The results described in this paragraph are independent of possible Lie group symmetries of the theory whose implications will be discussed in Section 2.2.

Let *E* be a vector space, whose elements will be denoted β since they are generalisations of the inverse temperature. The duality pairing between elements ν of the dual space $E^{*}$ and elements β∈E is denoted as $\langle \nu ,\beta \rangle$. Besides the vector space *E*, the setting also involves a manifold *M*, endowed with a volume form dμ.

Let $U:M\to E^{*}$ be a smooth function defined on *M* with values in $E^{*}$. Denote by Ω⊂E the largest open set such that for all β∈Ω the two integrals

(1) $$\int_{M}e^{-\langle U(m),\beta \rangle }d\mu \in R\;\mathrm{and}\;\int_{M}U\left(m\right)e^{-\langle U(m),\beta \rangle }d\mu \in E^{*}$$

converge. We denote by ψ:Ω→R the *partition function* (or *characteristic function*), given by

(2) $$\psi \left(\beta \right)=\int_{M}e^{-\langle U(m),\beta \rangle }d\mu .$$

For all β∈Ω, we consider the *generalized Gibbs probability densities*

(3) $$p_{\beta }\left(m\right)=\frac{1}{\psi (\beta )}e^{-\langle U(m),\beta \rangle }.$$

For application in information geometry it is required that $\beta \mapsto p_{\beta }$ is injective. It is important to note that the Gibbs densities are not defined on the whole vector space *E* but only on the open subset Ω. An element β∈Ω is called a *geometric temperature*. From now on we assume that Ω is not empty.

The *Massieu potential* is the function Φ:Ω→R defined by

(4) Φ(β)=−log(ψ(β))

from which we can write the generalized Gibbs probability densities as

$$p_{\beta }\left(m\right)=e^{\Phi \left(\beta \right)-\langle U(m),\beta \rangle },\;\forall \;\beta \in \Omega .$$

The *thermodynamic heat* $Q:\Omega \to E^{*}$ is the first derivative of the Massieu potential, i.e.,

(5) $$Q\left(\beta \right):=D\Phi \left(\beta \right)=\int_{M}U\left(m\right)p_{\beta }\left(m\right)d\mu =E_{\beta }\left[U\right]\in E^{*},$$

where $E_{\beta }$ denotes the expectation with respect to $p_{\beta }$.

We denote by $\Omega^{*}$ the image of the function Ω by *Q* and assume that $Q=D\Phi :\Omega \to \Omega^{*}$ is a diffeomorphism. In this case, we can define the *entropy*$s:\Omega^{*}\to R$ as the Legendre transform of the Massieu potential Φ:Ω→R, namely

(6) $$s\left(\nu \right):=\langle \nu ,\beta \rangle -\Phi \left(\beta \right),$$

where $\beta =Q^{-1}\left(\nu \right)$. In other words, β∈Ω in (6) is such that

DΦ(β)=ν.

The name entropy for this Legendre transform is justified by the following result.

**Lemma** **1.** *For every* β∈Ω *, we have the equality*

$$s\left(Q\left(\beta \right)\right)=S\left(p_{\beta }\right),$$

*where* Q(β) *is the thermodynamic heat and*

$$S\left(p\right)=-\int_{M}p\log p\;d\mu$$

*is the entropy of the probability density p.*

**Proof.**  On one hand, using the definition of *s* in Equation (6) and Φ in Equation (4), we have

$$s\left(Q\left(\beta \right)\right)=\langle Q(\beta ),\beta \rangle -\Phi \left(\beta \right)=\int_{M}\langle U(m),\beta \rangle p_{\beta }\left(m\right)d\mu +\log\left(\psi \left(\beta \right)\right).$$

On the other hand, we compute

$$\begin{array}{cc} S(p_{\beta }) & =-\int_{M}p_{\beta }\left(m\right)\log\left(p_{\beta }\left(m\right)\right)d\mu =-\int_{M}p_{\beta }\left(m\right)\left(-\log\left(\psi \left(\beta \right)\right)-\langle U(m),\beta \rangle \right)d\mu \\ \; & =\log\left(\psi \left(\beta \right)\right)\int_{M}p_{\beta }\left(m\right)d\mu +\int_{M}\langle U(m),\beta \rangle p_{\beta }\left(m\right)d\mu \\ \; & =\log\left(\psi \left(\beta \right)\right)+\int_{M}\langle U(m),\beta \rangle p_{\beta }\left(m\right)d\mu . \end{array}$$

These expressions are equal. ☐

Equation (6) is referred to as the *Clairaut equation*, see [15].

The *generalized heat capacity* is the symmetric tensor field K:Ω→sym(E), defined as minus the Hessian matrix of the Massieu potential, i.e.,

$$K\left(\beta \right):=-D^{2}\Phi \left(\beta \right)=D^{2}\log\psi \left(\beta \right):E\times E\to R.$$

A direct computation gives, for all vectors $\delta \beta_{1},\delta \beta_{2}\in E$,

$$\begin{array}{cc} K\left(\beta \right)\left(\delta \beta_{1},\delta \beta_{2}\right) & =-D^{2}\Phi \left(\beta \right)\left(\delta \beta_{1},\delta \beta_{2}\right)=-{\left.\frac{d}{d\varepsilon }\right|}_{\varepsilon =0}D\Phi \left(\beta +\varepsilon \delta \beta_{1}\right)\cdot \delta \beta_{2}\\ \; & =-{\left.\frac{d}{d\varepsilon }\right|}_{\varepsilon =0}\int_{M}\frac{\langle U\left(m\right),\delta \beta_{2}\rangle }{\psi (\beta +\varepsilon \delta \beta_{1})}e^{-\langle U\left(m\right),\beta +\varepsilon \delta \beta_{1}\rangle }d\mu \\ \; & =E_{\beta }\left[\langle U,\delta \beta_{1}\rangle \langle U,\delta \beta_{2}\rangle \right]-E_{\beta }\left[\langle U,\delta \beta_{1}\rangle \right]E_{\beta }\left[\langle U,\delta \beta_{2}\rangle \right] \end{array}$$

hence the generalized heat capacity is

$$\begin{array}{cc} K(\beta ) & =E_{\beta }\left[\left(U-E_{\beta }\left(U\right)\right)⊗\left(U-E_{\beta }\left(U\right)\right)\right]\\ \; & =E_{\beta }\left[(U-Q(\beta ))⊗(U-Q(\beta ))\right]. \end{array}$$

As a consequence, K(β) is positive semidefinite for all β∈Ω. Being the derivative of $Q:\Omega \to \Omega^{*}$, it is positive definite if *Q* is a diffeomorphism.

Recall that in information geometry, the *Fisher metric* associated with the family $p_{\beta }$, β∈Ω, of probability densities is the symmetric tensor field K:Ω→sym(E) defined by

$$I\left(\beta \right)=-E_{\beta }\left[D^{2}\log p_{\beta }\right].$$

In our setting we have the following identification.

**Proposition** **2.** *The generalized heat capacity of* $p_{\beta }$ *cocincides with the Fisher metric of* $p_{\beta }$ *:*

I(β)=K(β).

*In other words, the Fisher metric is the Hessian of the characteristic function logarithm* $K\left(\beta \right)=D^{2}\log\psi \left(\beta \right)$ *.*

**Proof.**  From Equation (3), we have

$$\log\left(p_{\beta }\left(m\right)\right)=-\log\psi \left(\beta \right)-\langle U(m),\beta \rangle$$

hence, taking the second derivative with respect to β we get

$$-D^{2}\log p_{\beta }=D^{2}\log\psi \left(\beta \right)=-D^{2}\Phi \left(\beta \right).$$

Please note that this equality does not depend on *m*, which proves that

$$I\left(\beta \right)=-E_{\beta }\left[D^{2}\log p_{\beta }\right]=-E_{\beta }\left(D^{2}\Phi \left(\beta \right)\right)=-D^{2}\Phi \left(\beta \right)=K\left(\beta \right).$$

Hence the result is proved. ☐

**Proposition** **3.** *Let us assume that*$Q:\Omega \to \Omega^{*}$*is a diffeomorphism. The inverse of the Fisher metric, i.e., the cometric on*$\Omega^{*}$*induced from the Fisher metric on* Ω*, is given by minus the Hessian of the entropy:*

$$-D^{2}s\left(\nu \right):E^{*}\times E^{*}\to R,\;\forall \;\nu \in \Omega^{*}.$$

**Proof.**  From the definition of the thermodynamic heat, we have $D\Phi \left(Q^{-1}\left(\nu \right)\right)\cdot \delta \beta =\langle \nu ,\delta \beta \rangle$, for every $\nu \in \Omega^{*}$ and δβ∈Ω. Taking the derivative with respect to ν, we get

(7) $$D^{2}\Phi \left(Q^{-1}\left(\nu \right)\right)\left(DQ^{-1}\left(\nu^{-1}\right)\cdot \delta \nu ,\delta \beta \right)=\langle \delta \nu ,\delta \beta \rangle .$$

Taking now the derivative of Equation (6), we get $Ds\left(\nu \right)=Q^{-1}\left(\nu \right)$ hence $D^{2}s\left(\nu \right)=DQ^{-1}\left(\nu \right)$. This can be used in Equation (7) and shows that $D^{2}\Phi \left(\beta \right)\cdot D^{2}s\left(\nu \right)=id_{E^{*}}$, where ν=Q(β). The result follows then from Proposition 2. ☐

The following result shows that the generalized Gibbs probability densities satisfy the maximum entropy principle [16].

**Proposition** **4** (Maximum entropy principle). *Let* $U:M\to E^{*}$ *be a smooth function and* $\nu \in \Omega^{*}\subset E^{*}$ *a given element. The generalized Gibbs probability density* $p_{\beta }$ *in Equation (3) with* $\beta =Q^{-1}\left(\nu \right)$ *is a solution of the maximum entropy principle:*

$$\max_{q}\left[-\int_{M}q\log q\;d\mu \right]\;such\;that\;\left\{\begin{array}{c} \;\int_{M}q\;d\mu =1\\ \int_{M}Uq\;d\mu =\nu . \end{array}\right.$$

**Proof.**  Given a probability density *q*, we have

$$\begin{array}{cc} -\int_{M}\left(q\log q-q\log p_{\beta }\right)d\mu & =-\int_{M}q\log\frac{q}{p_{\beta }}d\mu \leq -\int_{M}q\left(1-\frac{p_{\beta }}{q}\right)d\mu \\ \; & =-\int_{M}\left(q-p_{\beta }\right)d\mu =0. \end{array}$$

Hence, if *q* satisfies the constraints we get

$$\begin{array}{cc} -\int_{M}q\log qd\mu & \leq -\int_{M}q\log p_{\beta }d\mu =\int_{M}q\left(\log\psi \left(\beta \right)+\langle U(m),\beta \rangle \right)d\mu \\ \; & =\log\psi \left(\beta \right)+\langle \int_{M}Uqd\mu ,\beta \rangle =-\Phi \left(\beta \right)+\langle \nu ,\beta \rangle \\ \; & =\langle \nu ,Q^{-1}\left(\beta \right)\rangle -\Phi \left(Q^{-1}\left(\nu \right)\right)=S\left(\nu \right)=s\left(p_{\beta }\right). \end{array}$$

In the fourth equality we used $\beta =Q^{-1}\left(\nu \right)$, in the fifth equality we used definition Equation (6), and in the last equality we used Lemma 1. ☐

**Koszul-Vinberg characteristic function.** We now quickly describe a particular case of the above setting, which is related to Hessian geometry and in which the characteristic function Equation (2) recovers the Koszul-Vinberg characteirstic function, see [17,18,19,20,21] and the references in [3]. In this case, the Fisher information metric of information geometry coincides with the canonical Koszul Hessian metric given by Koszul forms. Analogies between Koszul-Vinberg model and Souriau symplectic model of statistical mechanics were enlightened in [3]. Here we will show how these two models precisely arise as special cases of the general setting presented in Section 2.1.

Let *E* be a vector space and Ω⊂E an open convex cone in *E*. The cone Ω is assumed to be regular, i.e., Ω contains no straight line, which is equivalent to the condition $\bar{\Omega }\cap \left(-\bar{\Omega }\right)=\left\{0\right\}$. We chose $M=\Omega^{*}\subset E^{*}$ as the dual cone defined by

$$\Omega^{*}:=\left\{\xi \in E^{*}|\langle \xi ,\beta \rangle >0,\;\forall \;\beta \in \bar{\Omega }-\left\{0\right\}\right\},$$

and we choose the function $U:M=\Omega^{*}\to E^{*}$ as the identity function on $\Omega^{*}$. We take the volume form as the Lebesgue measure dξ. The generalized Gibbs probability densities defined in Equation (3) are

(8) $$p_{\beta }\left(\xi \right)=\frac{1}{\psi (\beta )}e^{-\langle \xi ,\beta \rangle },$$

with characteristic function Equation (2) given by

(9) $$\psi \left(\beta \right)=\int_{\Omega^{*}}e^{-\langle \xi ,\beta \rangle }d\xi .$$

This expression recovers the Koszul-Vinberg characteristic function of the cone Ω, see [17,18,19,20,21,22,23]. We call Equation (8) the *Koszul density of the cone* Ω.

The *Koszul 1-form*, [18], defined as the differential of −logψ(β) coincides with the thermodynamic heat $Q:\Omega \to \Omega^{*}$ of the general setting above. It reads

$$Q\left(\beta \right)=\int_{\Omega^{*}}\xi p_{\beta }\left(\xi \right)d\xi =E_{\beta }\left(\xi \right).$$

The *Koszul metric* defined as the second derivative of logψ(β) coincides with the Fisher metric of information geometry from Proposition 2. It reads

$$\begin{array}{cc} I\left(\beta \right)\left(\delta \beta_{1},\delta \beta_{2}\right) & =\int_{\Omega^{*}}\langle \xi ,\delta \beta_{1}\rangle \langle \xi ,\delta \beta_{2}\rangle p_{\beta }\left(\xi \right)d\xi -\int_{\Omega^{*}}\langle \xi ,\delta \beta_{1}\rangle p_{\beta }\left(\xi \right)d\xi \int_{\Omega^{*}}\langle \xi ,\delta \beta_{2}\rangle p_{\beta }\left(\xi \right)d\xi . \end{array}$$

From Proposition 4, given $\nu \in \Omega^{*}$, the Koszul density of the cone Ω with $\beta =Q^{-1}\left(\nu \right)$, satisfies the maximum entropy principle

$$\max_{q}\left[-\int_{\Omega^{*}}q\log q\;d\xi \right]\;\mathrm{such}\;\mathrm{that}\;\left\{\begin{array}{c} \;\int_{\Omega^{*}}qd\xi =1\\ \int_{\Omega^{*}}\xi q\;d\xi =\nu , \end{array}\right.$$

see [3] for a direct proof.

An important example is $\Omega :={\mathrm{sym}}^{+}\left(n\right)\subset E=\mathrm{sym}\left(n\right)$, the cone of symmetric positive definite n×n matrices. The dual space is chosen as $E^{*}=\mathrm{sym}\left(n\right)$ with duality pairing $\langle \nu ,\beta \rangle =\mathrm{Tr}\left(\nu^{T}\beta \right)$. In this case, it is well-known that $\Omega^{*}=\Omega$. The generalized Gibbs probability densities are

$$p_{\beta }:{\mathrm{sym}}^{+}\left(n\right)\to R,\;p_{\beta }\left(\xi \right)=\frac{1}{\psi (\beta )}e^{-\langle \xi ,\beta \rangle },$$

where the Koszul-Vinberg characteristic function can be explicitly computed as

$$\psi \left(\beta \right)=\int_{{\mathrm{sym}}^{+}\left(n\right)}e^{-\langle \xi ,\beta \rangle }d\xi =\det{\left(\beta \right)}^{-\frac{n+1}{2}}\psi \left(I_{n}\right).$$

The Massieu potential is deduced as

(10) $$\Phi \left(\beta \right)=-\log\left(\psi \left(\beta \right)\right)=\frac{n+1}{2}\log\left(\det\left(\beta \right)\right)-\log\left(\psi \left(I_{n}\right)\right)$$

and the thermodynamic heat and entropy are

$$Q\left(\beta \right)=D\Phi \left(\beta \right)=\frac{n+1}{2}\beta^{-1}=E_{\beta }\left(\xi \right)$$

$$s\left(\nu \right)=\frac{n+1}{2}\log\left(\det\left(\nu \right)\right)+\frac{n(n+1)}{2}\left(1-\log\frac{n+1}{2}\right)+\log\left(\psi \left(I_{n}\right)\right).$$

We can thus write the generalized Gibbs probability densities as

$$\begin{array}{cc} p_{\beta }\left(\xi \right) & =\frac{1}{\psi (\beta )}e^{-\langle \xi ,\beta \rangle }=\det{\left(\beta \right)}^{\frac{n+1}{2}}\frac{1}{\psi (I_{n})}e^{-\langle \xi ,\beta \rangle }. \end{array}$$

Finally, the expression of the Fisher metric on Ω is found by using Equation (10) as

(11) $$I\left(\beta \right)\left(\delta \beta_{1},\delta \beta_{2}\right)=-D^{2}\Phi \left(\beta \right)\left(\delta \beta_{1},\delta \beta_{2}\right)=\frac{n+1}{2}\mathrm{Tr}\left(\beta^{-1}\delta \beta_{1}\beta^{-1}\delta \beta_{2}\right),$$

for every $\delta \beta_{1},\delta \beta_{2}\in E$.

### 2.2. Equivariance with Respect to Lie Group Actions

In this section, we study the consequences of the equivariance of the function *U* appearing in the generalized Gibbs probability densities. More precisely, given a Lie group *G*, we assume that $U:M\to E^{*}$ is *G*-equivariant with respect to an action of the Lie group on *M* and an affine action of the Lie group on $E^{*}$. This setting includes as special cases the Souriau symplectic model of statistical mechanics [24], its polysymplectic extension [5], the case of multivariate Gaussian densities, as treated for instance in [4], and approaches developed in quantum information geometry [25], for which the Fisher metric will be shown to coincide with the Bogoliubov-Kubo-Mori metric in Section 3.2.

Let *G* be a Lie group, and let

$$\phi :G\times M\to M,\;\left(g,m\right)\mapsto \phi_{g}\left(m\right)$$

be a left action of *G* on *M*, i.e., ϕ satisfies

$$\phi_{e}=id_{M}\;\mathrm{and}\;\phi_{g}\circ \phi_{h}=\phi_{gh},$$

for every g,h∈G, with $id_{M}$ the identity on *M*. We denote by g the Lie algebra of *G*. The infinitesimal generator of the action corresponding to ξ∈g is the vector field $\xi_{M}$ on *M* defined by

(12) $$\xi_{M}\left(m\right)={\left.\frac{d}{d\varepsilon }\right|}_{\varepsilon =0}\phi_{\exp(\varepsilon \xi )}\left(m\right),$$

for every m∈M, where exp:g→G is the Lie group exponential map.

We also consider a left linear action

$$\rho :G\times E\to E,\;\left(g,\beta \right)\mapsto \rho_{g}\left(\beta \right)$$

of *G* on the vector space *E*, $\rho_{g}\in L\left(E,E\right)$. We denote by $\rho^{*}:G\times E^{*}\to E^{*}$ the linear right action of *G* induced on the dual space $E^{*}$

(13) $$\langle \rho_{g}^{*}\left(\nu \right),\beta \rangle =\langle \nu ,\rho_{g}\left(\beta \right)\rangle ,\;\forall \;\beta \in E,\;\nu \in E^{*},\;g\in G.$$

We recall that a *group one-cocycle with respect to $\rho^{*}$* is a map $\theta \in C^{\infty }\left(G,E^{*}\right)$ such that

(14) $$\theta \left(gh\right)=\theta \left(g\right)+\rho_{g^{-1}}^{*}\left(\theta \left(h\right)\right),$$

for every g,h∈G. Equivalently, a group one-cocycle $\theta \in C^{\infty }\left(G,E^{*}\right)$ with respect to $\rho^{*}$ is such that $A:G\times E^{*}\to E^{*}$ defined by

(15) $$A_{g}\left(\nu \right)=\rho_{g^{-1}}^{*}\left(\nu \right)+\theta \left(g\right)$$

is an affine left action of *G* on $E^{*}$.

Finally, we recall that the Jacobian of the action $\phi_{g}:M\to M$ relative to the volume form dμ is the function $J\phi_{g}:M\to R$ defined by $\phi_{g}^{*}d\mu =J\phi_{g}d\mu$, where $\phi_{g}^{*}$ denotes the pull-back of the *n*-form dμ by the diffeomorphism $\phi_{g}$. We will be interested in actions which satisfy $J\phi_{g}=c\left(g\right)$ is a constant on *M*. Please note that c(gh)=c(g)c(h), for every g,h∈G. The particular case c(g)=1 corresponds to volume preserving diffeomorphisms.

**Proposition** **5.** *Assume that the action ϕ of G on M satisfies* $\phi_{g}^{*}\mu =c\left(g\right)\mu$ *and the function U is G-equivariant:*

(16) $$U\left(\phi_{g}\left(m\right)\right)=\rho_{g^{-1}}^{*}\left(U\left(m\right)\right)+\theta \left(g\right),$$

*for all* g∈G *and* m∈M *, where* $\theta \in C^{\infty }\left(G,E^{*}\right)$ *is a group one-cocycle. Then the open subset* Ω⊂E *is invariant under the action of G on E, the partition function ψ satisfies*

$$\psi \left(\rho_{g}\left(\beta \right)\right)=\psi \left(\beta \right)c\left(g\right)e^{\langle \theta (g^{-1}),\beta \rangle }$$

*for every* g∈G *, and the probability density* $p_{\beta }$ *satisfies*

$$\phi_{g}^{*}p_{\beta }=p_{\rho_{g}^{-1}\left(\beta \right)},$$

*for every* g∈G *, where* $\phi_{g}^{*}p_{\beta }=\left(p_{\beta }\circ \phi_{g}\right)J\phi_{g}=\left(p_{\beta }\circ \phi_{g}\right)c\left(g\right)$ *is the pull-back of a density.*

*As a consequence, the Massieu potential* Φ(β) *, the thermodynamic heat* Q(β) *, the entropy* s(ν) *, and the heat capacity* K(β) *satisfy the following equivariance properties*

(17) $$\begin{array}{cc} \; & \Phi \left(\rho_{g}\left(\beta \right)\right)=\Phi \left(\beta \right)-\log\left(c\left(g\right)\right)-\langle \theta (g^{-1}),\beta \rangle \end{array}$$

(18) $$\begin{array}{cc} \; & Q\left(\rho_{g}\left(\beta \right)\right)=\rho_{g^{-1}}^{*}\left(Q\left(\beta \right)\right)+\theta \left(g\right) \end{array}$$

(19) $$\begin{array}{cc} \; & s\left(\rho_{g^{-1}}^{*}\left(\nu \right)+\theta \left(g\right)\right)=s\left(\nu \right)+\log\left(c\left(g\right)\right) \end{array}$$

(20) $$\begin{array}{cc} \; & K\left(\rho_{g}\left(\beta \right)\right)\left(\rho_{g}\left(\delta \beta_{1}\right),\rho_{g}\left(\delta \beta_{2}\right)\right)=K\left(\beta \right)\left(\delta \beta_{1},\delta \beta_{2}\right), \end{array}$$

*for every* g∈G *.*

**Proof.**  Using Equation (16) and a change of variables, we have

$$\begin{array}{cc} \psi (\rho_{g}\left(\beta \right)) & =\int_{M}e^{-\langle U\left(m\right),\rho_{g}\left(\beta \right)\rangle }d\mu =\int_{M}e^{-\langle \rho_{g}^{*}\left(U\left(m\right)\right),\beta \rangle }d\mu \\ \; & =\int_{M}e^{-\langle U\left(\phi_{g^{-1}}\left(m\right)\right)-\theta \left(g^{-1}\right),\beta \rangle }d\mu =\int_{M}e^{-\langle U(\phi_{g^{-1}}\left(m\right)),\beta \rangle }d\mu \;e^{\langle \theta (g^{-1}),\beta \rangle }\\ \; & =\int_{M}e^{-\langle U(m),\beta \rangle }J\phi_{g}d\mu \;e^{\langle \theta (g^{-1}),\beta \rangle }=\psi \left(\beta \right)c\left(g\right)e^{\langle \theta (g^{-1}),\beta \rangle }. \end{array}$$

The other statement are checked in a similar way, by using Equations (13)–(16). ☐

This proposition unifies in a single statement, several Lie group equivariance properties observed in several models for information geometry and Lie group machine learning, see, e.g., [3,4,5,7,8]. Before discussing the symplectic and polysymplectic models we illustrate below these equivariance properties for the Koszul model recalled above.

**Equivariance in the Koszul model.** For the Koszul model recalled above, see [3] and references therein, G=Aut(Ω) is the group of linear isomorphism that preserves Ω⊂E. Given g∈Aut(Ω), we have $\rho_{g}:\Omega \to \Omega$ and it is clear that the dual action $\rho_{g}^{*}$ preserves the dual cone $\Omega^{*}$. In this very special case, $M=\Omega^{*}$ and the *G* action on *M* is chosen as $\phi_{g}:=\rho_{g^{-1}}^{*}$. Since $U:\Omega^{*}\to E^{*}$ is the identity, there is no cocycle. However, we have $c\left(g\right)=J\phi_{g}$ which is not equal to one in general and, for instance, the transformation Equation (17) of the Massieu potential reads

$$\Phi \left(\rho_{g}\left(\beta \right)\right)=\Phi \left(\beta \right)-\log\left(c\left(g\right)\right).$$

Let us consider as special case the cone of symmetric positive definite matrices $\Omega ={\mathrm{sym}}^{+}\left(n\right)\subset E=\mathrm{sym}\left(n\right)$. The dual space is chosen as $E^{*}=\mathrm{sym}\left(n\right)$ with duality pairing $\langle \nu ,\beta \rangle =\mathrm{Tr}\left(\nu^{T}\beta \right)$ and we have $\Omega^{*}=\Omega$.

We consider the left action of GL(n) on E=sym(n) given by

(21) $$\rho_{A}\left(\beta \right)=A^{-T}\beta A^{-1}.$$

Therefore, we have

(22) $$\rho_{A}^{*}\left(\nu \right)=A^{-1}\nu A^{-T}\;\mathrm{and}\;\phi_{A}\left(\xi \right)=\rho_{A^{-1}}^{*}\left(\xi \right)=A\xi A^{T}.$$

Proposition 5 directly yields the following equivariance properties

$$\begin{array}{cc} \; & \psi \left(A^{-T}\beta A^{-1}\right)=\psi \left(\beta \right)c\left(A\right)\\ \; & p_{\beta }\left(A\xi A^{T}\right)c\left(A\right)=p_{A^{T}\beta A}\left(\xi \right)\\ \; & \Phi \left(A^{-T}\beta A^{-1}\right)=\Phi \left(\beta \right)-\log c\left(A\right)\\ \; & Q\left(A^{-T}\beta A^{-1}\right)=AQ\left(\beta \right)A^{T}\\ \; & s\left(A\nu A^{T}\right)=s\left(\nu \right)+\log c\left(A\right), \end{array}$$

for all A∈GL(n), where $c\left(A\right)={\left(\det A\right)}^{n+1}$.

### 2.3. Souriau Symplectic Model of Statistical Mechanics

In this section, we show that the Souriau symplectic model of statistical mechanics [24] arises as a special case of the preceding setting, by considering (M,ω) a symplectic manifold and dμ the Liouville form associated with ω.

We then exploit this setting to show that the entropy in the Souriau model is a *Casimir function* of the Lie-Poisson bracket with Lie algebra cocycle associated with the nonequivariance cocycle of the momentum map, i.e., it Poisson commutes with every functions. Based on this we formulate a dynamical geometric model for dissipation/production of this Casimir, following the Lie algebraic setting proposed in [26,27]. This allows us to clarify the link between the geometry underlying Souriau symplectic models and that underlying models proposed in [25] in the framework of quantum physics by information geometry for some Lie algebras, see also [28]. Details will be given in Section 3.2. Finally, we present a stochastic perturbation of the Lie-Poisson equations with cocycle within the setting of stochastic Hamiltonian dynamics.

To present the Souriau model, we first quickly recall below the notion of momentum map and nonequivariance cocycle for symplectic manifolds, see, e.g., [29,30,31]. Consider a symplectic manifold (M,ω), i.e., a manifold *M* endowed with a closed non-degenerate two form ω. The associated Liouville form is $d\mu =\frac{{\left(-1\right)}^{n(n-1)/2}}{n!}\omega \wedge \ldots \wedge \omega$ (*n* times), where 2n=dimM. Given a function h:M→R, the Hamiltonian vector field associated with *H* is the vector field $X_{h}$ defined by

(23) $$i_{X_{h}}\omega =dh.$$

Recall that the symplectic form ω defines the Poisson bracket (see Remark 7)

(24) $$\left\{f,g\right\}=\omega \left(X_{f},X_{g}\right)$$

on functions $f,g\in C^{\infty }\left(M\right)$.

A Lie group action ϕ:G×M→M of *G* on *M* is symplectic, if it preserves the symplectic form, i.e., $\phi_{g}^{*}\omega =\omega$, for every g∈G. Taking the derivative of this identity with respect to *g* at g=e, we get ${£}_{\xi_{M}}\omega =0$, for every ξ∈g, where $\xi_{M}$ is the infinitesimal generator associated with the Lie algebra element ξ∈g, see Equation (12), and *£* is the Lie derivative. Equivalently, we have

$$d(i_{\xi_{M}}\omega )=0,$$

for every ξ∈g, i.e., the one-form $i_{\xi_{M}}\omega$ is locally exact. If it is globally exact, i.e., if $\xi_{M}$ is a Hamiltonian vector field for every ξ∈g, then the action is called *Hamiltonian* and admits a *momentum map*$J:M\to g^{*}$, which satisfies

$$i_{\xi_{M}}\omega =dJ_{\xi },$$

where $J_{\xi }:M\to R$ is defined by $J_{\xi }\left(m\right):=\langle J(m),\xi \rangle$, for every ξ∈g.

When *M* is connected, there is a well-defined group one-cocycle $\theta :G\to g^{*}$, called the *nonequivariance cocycle*, given by

$$\theta \left(g\right)=J\left(\Phi_{g}\left(m\right)\right)-{\mathrm{Ad}}_{g^{-1}}^{*}\left(J\left(m\right)\right),$$

where m∈M can be arbitrarily chosen. It characterizes the nonequivariance of the momentum map with respect to the action of *G* on *M* and the coadjoint action of *G* on $g^{*}$. The group one-cocycle property is

$$\theta \left(gh\right)=\theta \left(g\right)+{\mathrm{Ad}}_{g^{-1}}^{*}\left(\theta \left(h\right)\right),$$

for every g,h,∈G. We consider its differential $\Theta :=T_{e}\theta$ seen as a map Θ:g×g→R, i.e.,

(25) $$\Theta \left(\xi ,\eta \right)=\langle T_{e}\theta \left(\xi \right),\eta \rangle ={\left.\frac{d}{d\varepsilon }\right|}_{\varepsilon =0}\langle \theta (\exp(\varepsilon \xi )),\eta \rangle .$$

Taking the derivative of the relation above, we get

(26) $$\Theta \left(\xi ,\eta \right)=J_{\left[\xi ,\eta \right]}-\left\{J_{\xi },J_{\eta }\right\},$$

where the last term uses the Poisson bracket Equation (24). The map Θ:g×g→R is bilinear, skew-symmetric, and, as can be readily verified, satisfies the *Lie algebra two-cocycle identity*

(27) Θ([ξ,η],ζ)+Θ([η,ζ],ξ)+Θ([ζ,ξ],η)=0.

We refer to [29,30,31] for detailed introductions to these concepts.

**Remark** **6** (Lie group and Lie algebra cohomology). A group one-cocycle $\theta \in C^{\infty }\left(G,g^{*}\right)$ is called a *group one-coboundary* if there is a $\lambda \in g^{*}$ such that

$$\theta \left(g\right)=\lambda -{\mathrm{Ad}}_{g^{-1}}^{*}\lambda$$

for every g∈G. The quotient space of one-cocycles modulo one-coboundaries is called the *first group cohomology of G* and is denoted by $H^{1}\left(G,g^{*}\right)$. These definitions extend to arbitrary representation of *G* on a vector space, as in Equation (14).

A Lie algebra two-cocycle Θ is called a *Lie algebra two-coboundary* if there is $\lambda \in g^{*}$ such that

$$\Theta \left(\xi ,\eta \right)=\langle \lambda ,[\xi ,\eta ]\rangle ,$$

for all ξ,η∈g. The quotient space of Lie algebra two-cocycles by Lie algebra two-coboundaries is called the *second Lie algebra cohomology of g* and is denoted by $H^{2}\left(g,R\right)$.

#### 2.3.1. Souriau Symplectic Model of Satistical Mechanics

The *Souriau symplectic model of statistical mechanics* is obtained by considering the following specific situation in the setting described in Section 2.2:

$$\begin{array}{cc} M: & \;a\;\mathrm{symplectic}\;\mathrm{manifold}\\ d\mu : & \;\mathrm{the}\;\mathrm{Liouville}\;\mathrm{volume}\\ \phi_{g}: & \;a\;\mathrm{Hamiltonian}\;\mathrm{action}\\ E=g: & \;\mathrm{the}\;\mathrm{Lie}\;\mathrm{algebra}\;\mathrm{of}\;G\\ \rho_{g}={\mathrm{Ad}}_{g}: & \;\mathrm{the}\;\mathrm{adjoint}\;\mathrm{action}\;\mathrm{of}\;G\;\mathrm{on}\;g\\ U=J:M\to g^{*}: & \;a\;\mathrm{momentum}\;\mathrm{map}. \end{array}$$

In particular, the thermodynamic heat becomes $Q\left(\beta \right)=E_{\beta }\left(J\right)$ and the Fisher metric on Ω⊂g is

$$I\left(\beta \right)=E_{\beta }\left(\left(J-E_{\beta }\left(J\right)\right)⊗\left(J-E_{\beta }\left(J\right)\right)\right)\in \mathrm{sym}\left(g\right).$$

Proposition 5 directly yields the following equivariance properties

$${\mathrm{Ad}}_{g}\Omega =\Omega ,\;\psi \left({\mathrm{Ad}}_{g}\beta \right)=\psi \left(\beta \right)e^{\langle \theta (g^{-1}),\beta \rangle },\;p_{\beta }\circ \phi_{g}=p_{{\mathrm{Ad}}_{g^{-1}}\beta }$$

and

(28) $$\begin{array}{cc} \; & \Phi \left({\mathrm{Ad}}_{g}\beta \right)=\Phi \left(\beta \right)-\langle \theta (g^{-1}),\beta \rangle \end{array}$$

(29) $$\begin{array}{cc} \; & Q\left({\mathrm{Ad}}_{g}\beta \right)={\mathrm{Ad}}_{g^{-1}}^{*}\left(Q\left(\beta \right)\right)+\theta \left(g\right) \end{array}$$

(30) $$\begin{array}{cc} \; & s({\mathrm{Ad}}_{g^{-1}}^{*}\nu +\theta \left(g\right))=s\left(\nu \right) \end{array}$$

(31) $$\begin{array}{cc} \; & K\left({\mathrm{Ad}}_{g}\beta \right)\left({\mathrm{Ad}}_{g}\delta \beta_{1},{\mathrm{Ad}}_{g}\delta \beta_{2}\right)=K\left(\beta \right)\left(\delta \beta_{1},\delta \beta_{2}\right), \end{array}$$

for every g∈G. Note also that $\Omega^{*}$ is invariant under the affine action $\nu \mapsto {\mathrm{Ad}}_{g^{-1}}^{*}\nu +\theta \left(g\right)$.

From Proposition 4, given $\nu \in \Omega^{*}\subset g^{*}$, the generalized Gibbs probability density

$$p_{\beta }\left(m\right)=\frac{1}{\psi (\beta )}e^{-\langle J(m),\beta \rangle },$$

with $\beta =Q^{-1}\left(\nu \right)$, satisfies the maximum entropy principle

$$\max_{q}\left[-\int_{M}q\log q\;d\mu \right]\;\mathrm{such}\;\mathrm{that}\;\left\{\begin{array}{c} \;\int_{M}q\;d\mu =1\\ \int_{M}Jq\;d\mu =\nu . \end{array}\right.$$

We refer to [2] for a detailed presentation of Souriau’s model. We refer to [32] for recent developments exploiting Souriau’s model. As mentioned earlier in the general case, it is important to note that the generalized Gibbs densities are not defined on the whole Lie algebra g but only on the open subset Ω⊂g of geometric temperatures. As already observed by Souriau the set Ω can be empty in some examples, such as the case of the action of the Galilean group. In this case, Souriau’s method considers Gibbs densities associated with one-parameter subgroups of the acting Lie group.

#### 2.3.2. Lie-Poisson Equations with Cocycle and Property of the Entropy in Souriau’s Model

From Equation (30), we note that the entropy *s* is constant on the affine coadjoint orbits defined by

(32) $$O=\{{\mathrm{Ad}}_{g^{-1}}^{*}\mu_{0}+\theta \left(g\right)|g\in G\},$$

for $\mu_{0}\in g^{*}$. It is well-known that affine coadjoint orbits are symplectic manifolds, with symplectic form given by

(33) $$\omega_{O}\left(\mu \right)\left({\mathrm{ad}}_{\xi }^{*}\mu -\Theta \left(\xi ,\cdot \right),{\mathrm{ad}}_{\eta }^{*}\mu -\Theta \left(\eta ,\cdot \right)\right)=\langle \mu ,[\xi ,\eta ]\rangle -\Theta \left(\xi ,\eta \right),$$

for μ∈O, ξ,η∈g. This is an extension to the affine case of the well-known *Kirilov-Kostant-Souriau symplectic form* on coadjoint orbits. The connected components of the affine coadjoint orbits Equation (32) are the symplectic leaves in the Poisson manifold $\left(g^{*},{\left\{\;,\right\}}_{\Theta }\right)$, where ${\left\{\;,\right\}}_{\Theta }$ is the *Lie-Poisson bracket with cocycle* (or *affine Lie-Poisson bracket*)

(34) $${\left\{f,g\right\}}_{\Theta }\left(\mu \right)=\langle \mu ,\left[\frac{\delta f}{\delta \mu },\frac{\delta g}{\delta \mu }\right]\rangle -\Theta \left(\frac{\delta f}{\delta \mu },\frac{\delta g}{\delta \mu }\right),\;f,g:g^{*}\to R,$$

see, e.g., [29].

The Hamiltonian system (see Remark 7) associated with the Lie-Poisson bracket with cocycle Equation (34) and to a given Hamiltonian function $h:g^{*}\to R$ is given by the *Lie-Poisson equations with cocycle* (or *affine Lie-Poisson equations*)

(35) $$\frac{d}{dt}f={\left\{f,h\right\}}_{\Theta },\;\forall \;f:g^{*}\to R,$$

which yield the dynamical system

(36) $$\frac{d}{dt}\mu +{\mathrm{ad}}_{\frac{\delta h}{\delta \mu }}^{*}\mu =\Theta \left(\frac{\delta h}{\delta \mu },\cdot \right),$$

for a curve $\mu \left(t\right)\in g^{*}$. This dynamical system preserves each affine coadjoint orbit Equation (32) and defines on each of them a Hamiltonian system with respect to the symplectic form Equation (33). The Lie-Poisson equations with cocycle have important applications, in particular they appear in the geometric formulation of complex fluids, see [33,34,35], and geometrically exact (Cosserat) rods, see [36,37]. See [38] for another point of view on Lie-Poisson equations with cocycle. These equations are also referred to as *affine Lie-Poisson equations* or *Lie-Poisson equations with non-zero cohomology*.

**Remark** **7** (Poisson brackets and reduction, see [31]). Recall that a Poisson bracket on a manifold *M* is a Lie algebra structure {·,·} on $C^{\infty }\left(M\right)$ which is a derivation in each factor: {fg,h}=f{g,h}+{f,h}g. For instance, a symplectic structure ω on *M* defines the Poisson bracket $\left\{f,g\right\}=\omega \left(X_{f},X_{g}\right)$ for $f,g\in C^{\infty }\left(M\right)$. Another example is the Lie-Poisson bracket

(37) $$\left\{f,g\right\}\left(\mu \right)=\pm \langle \mu ,\left[\frac{\delta f}{\delta \mu },\frac{\delta g}{\delta \mu }\right]\rangle \;f,g\in C^{\infty }\left(g^{*}\right)$$

on the dual of any Lie algebra g, as well as its affine modified version Equation (34) by a two-cocycle Θ. The Hamiltonian system associated with a Poisson bracket and a given Hamiltonian $h\in C^{\infty }\left(M\right)$ is the dynamical system characterized by the condition

$$\frac{d}{dt}f=\left\{f,h\right\}$$

for every functions $f\in C^{\infty }\left(M\right)$, see for instance Equations (35) and (36).

An important point for applications in mechanics is the understanding of such Poisson structures *as being induced from a canonical symplectic form* (or, equivalently, from the associated canonical Poisson bracket) on a cotangent bundle, via reduction by symmetry relative to a Lie group action. This is the case for the Lie-Poisson bracket Equation (37) which is induced by the canonical symplectic form on $T^{*}G$ and the action of *G* on $T^{*}G$ given by the cotangent lifted action of right or left translation. The Lie-Poisson bracket with cocycle Equation (34) is induced by the canonical symplectic form on $T^{*}G$ and an affine modified cotangent lifted action of right or left translation ([33]).

**Corollary** **8.** *The entropy s of the Souriau model is a Casimir function for the Lie-Poisson bracket with cocycle Equation (36), i.e., it satisfies*

$${\left\{s,f\right\}}_{\Theta }=0,$$

*for every smooth functions* $f:g^{*}\to R$ *.*

**Proof.**  From Equation (30), we have

$$\langle \frac{\delta s}{\delta \mu },-{\mathrm{ad}}_{\xi }^{*}\mu +\Theta \left(\xi ,\cdot \right)\rangle =0$$

for all ξ∈g. This is equivalent to ${\mathrm{ad}}_{\frac{\delta s}{\delta \mu }}^{*}\mu -\Theta \left(\frac{\delta s}{\delta \mu },\cdot \right)=0$, which shows that ${\left\{s,f\right\}}_{\Theta }=0$, for all *f*. ☐

As a consequence of the above, the information manifold foliates into level sets of the entropy, containing a family of coadjoint orbits, that could be interpreted in Thermodynamics: motion remaining on theses level sets is non-dissipative, whereas motion transversal to these level sets is dissipative. The affine Kirillov-Kostant-Souriau form makes each orbit into a homogeneous symplectic manifold. Hamiltonian motion on these affine coadjoint orbits is given by the solutions of the Lie-Poisson equations with cocycle Equation (36). We shall present below a geometric way to introduce dissipation and hence, motion through affine coadjoint orbits.

**Elementary examples.** A particularly simple case of Souriau symplectic model is when the symplectic manifold is a cotangent bundle $M=T^{*}Q$ endowed with the canonical symplectic form. Let *G* be a Lie group acting on the left on *Q*. Then its cotangent lifted action on $T^{*}Q$ is symplectic and admits the momentum map $J:T^{*}Q\to g^{*}$ given by

(38) $$\langle J(\alpha_{q}),\xi \rangle =\langle \alpha_{q},\xi_{Q}\left(q\right)\rangle .$$

In this case, there is no cocycle, which yields obvious simplifications in the properties Equations (28)–(30).

Another case without cocycle is when *M* is an affine coadjoint orbit $M=O=\{{\mathrm{Ad}}_{g^{-1}}^{*}\mu +\theta \left(g\right)|g\in G\}$ endowed with the symplectic form Equation (33). In this case, the momentum map is simply the inclusion $J:O\to g^{*}$ of the affine coadjoint orbit in the dual of the Lie algebra $g^{*}$, [29]. While this example is simple, it plays an important role in the applications, e.g., [8,39]. An example with nonequivariance cocycle will be treated in detail in Section 3.3 for the special Euclidean group of the plane.

#### 2.3.3. Dynamics with Casimir Dissipation/Production

We take advantage of the Casimir function *s* associated with the Souriau model, to formulate a dynamical geometric model for dissipation/production of this Casimir. This allows us to clarify the link between Souriau symplectic models and models proposed in [25] in the framework of quantum physics by information geometry for some Lie algebras, see also [28].

We follow the general Lie algebraic approach developed in [26,27] for Casimir dissipation, slightly extended here to take into account of a cocycle, and to a wider class of dissipation.

Given a symmetric positive bilinear form γ:g×g→R, a Hamiltonian $h:g^{*}\to R$, a parameter Λ≠0, and a function $k:g^{*}\to R$ such that

(39) $$\left[\frac{\delta h}{\delta \mu },\frac{\delta k}{\delta \mu }\right]=0,$$

we consider the modification of the Lie-Poisson equations with cocycle Equation (35) given by

(40) $$\frac{d}{dt}f={\left\{f,h\right\}}_{\Theta }-\Lambda \;\gamma \left(\left[\frac{\delta f}{\delta \mu },\frac{\delta k}{\delta \mu }\right],\left[\frac{\delta s}{\delta \mu },\frac{\delta k}{\delta \mu }\right]\right)$$

for every *f*. We denote by $♭:g\mapsto g^{*}$ the flat operator associated with γ. That is, the linear form $\xi^{♭}\in g^{*}$ is given by $\xi^{♭}\left(\eta \right)=\gamma \left(\xi ,\eta \right)$, for all ξ,η∈g. Please note that the flat operator need not be either injective or surjective. Using the equality

$$\begin{array}{cc} -\gamma \left(\left[\frac{\delta f}{\delta \mu },\frac{\delta k}{\delta \mu }\right],\left[\frac{\delta s}{\delta \mu },\frac{\delta k}{\delta \mu }\right]\right) & =-\langle {\left[\frac{\delta s}{\delta \mu },\frac{\delta k}{\delta \mu }\right]}^{♭},\left[\frac{\delta f}{\delta \mu },\frac{\delta k}{\delta \mu }\right]\rangle =\langle {\mathrm{ad}}_{\frac{\delta k}{\delta \mu }}^{*}{\left[\frac{\delta s}{\delta \mu },\frac{\delta k}{\delta \mu }\right]}^{♭},\frac{\delta f}{\delta \mu }\rangle , \end{array}$$

Equation (40) yields the dynamical system

(41) $$\frac{d}{dt}\mu +{\mathrm{ad}}_{\frac{\delta h}{\delta \mu }}^{*}\mu =\Theta \left(\frac{\delta h}{\delta \mu }\right)+\Lambda {\mathrm{ad}}_{\frac{\delta k}{\delta \mu }}^{*}{\left[\frac{\delta s}{\delta \mu },\frac{\delta k}{\delta \mu }\right]}^{♭}.$$

For Θ=0 and h=k, this is the model proposed in [26,27] and applied there in the infinite dimensional setting, with applications to geophysical fluids and magnetohydrodynamics.

The main properties of system Equation (41) are the following.

(i) *Energy conservation*: taking f=h in Equation (40), we obtain
  $$\frac{d}{dt}h={\left\{h,h\right\}}_{\Theta }-\Lambda \;\gamma \left(\left[\frac{\delta h}{\delta \mu },\frac{\delta k}{\delta \mu }\right],\left[\frac{\delta s}{\delta \mu },\frac{\delta k}{\delta \mu }\right]\right)=0$$
  because of Equation (39) and since ${\left\{h,h\right\}}_{\Theta }=0$. Hence the total energy *h* is preserved.
(ii) *Casimir dissipation (*Λ>0*) or production (*Λ<0*)*: taking f=s in Equation (40), and using ${\left\{s,f\right\}}_{\Theta }=0$, we obtain
  $$\frac{d}{dt}s={\left\{s,h\right\}}_{\Theta }-\Lambda \;\gamma \left(\left[\frac{\delta s}{\delta \mu },\frac{\delta h}{\delta \mu }\right],\left[\frac{\delta s}{\delta \mu },\frac{\delta k}{\delta \mu }\right]\right)=-\Lambda {∥\left[\frac{\delta s}{\delta \mu },\frac{\delta k}{\delta \mu }\right]∥}^{2}\leq 0\;\;/\;\geq 0,$$
  where ${∥\xi ∥}^{2}=\gamma \left(\xi ,\xi \right)$.

We will explain in Section 3.2 how system Equation (41) recovers the model proposed in [25] in the context of information geometry for quantum systems for Lie algebras with unitary representation.

#### 2.3.4. Stochastic Hamiltonian Dynamics

We shall briefly discuss here a stochastic perturbation of the Lie-Poisson equation with cocycle Equation (35) within the setting of *stochastic Hamiltonian dynamics*, see [40,41,42], which preserves the affine coadjoint orbits. This theory was recently extended for stochastic geometric modeling in fluid dynamics via variational principles in [43], see also [44,45].

In the context of this paper, this stochastic extension is motivated in geometric statistical mechanics to model Gibbs density in the case of centrifuge with random vibration along the axis (that is an open problem for industrial centrifuge, because for large equipment it is difficult to reduce vibration of this axis). In statistical machine learning, the problem is motivated for small data analytics, where the Gibbs density as maximum entropy of first order is an approximation. In this case, there is some fluctuations in estimation of mean momentum map due to the fact that the true Gibbs density is a density of higher order. This approximation could be modeled by an additional noise on the moment map.

In the setting of the Lie-Poisson equations with cocycle given in Equation (35), we consider the stochastic Hamiltonian dynamics given by

(42) $$df={\left\{f,h\right\}}_{\Theta }dt+\sum_{i=1}^{N}{\left\{f,h_{i}\right\}}_{\Theta }\circ dW_{i}\left(t\right),$$

where $h_{i}:g^{*}\to R$, i=1,…,N are given Hamiltonians and $W_{i}\left(t\right)$, i=1,…,N are independent Brownian motions introduced in the Stratonovich sense, as indicated by the symbol ∘. Please note that the contribution of each Hamiltonian is inserted via the Lie-Poisson bracket with cocycle ${\left\{\cdot ,\cdot \right\}}_{\Theta }$ given in Equation (34). This results in the following Stratonovich differential equation for the stochastic process $\mu \left(t\right)\in g^{*}$

(43) $$d\mu +\left[{\mathrm{ad}}_{\frac{\delta h}{\delta \mu }}^{*}\mu -\Theta \left(\frac{\delta h}{\delta \mu },\cdot \right)\right]dt+\sum_{i=1}^{N}\left[{\mathrm{ad}}_{\frac{\delta h_{i}}{\delta \mu }}^{*}\mu -\Theta \left(\frac{\delta h_{i}}{\delta \mu },\cdot \right)\right]\circ dW_{i}\left(t\right)=0.$$

The Itô form of Equation (43) can be obtained by the usual conversion formula. It can be expressed in a concise and general way as

$$df=\left({\left\{f,h\right\}}_{\Theta }-\frac{1}{2}{\left\{h_{i},{\left\{h_{i},f\right\}}_{\Theta }\right\}}_{\Theta }\right)dt+\sum_{i=1}^{N}{\left\{f,h_{i}\right\}}_{\Theta }dW_{i}\left(t\right).$$

In a similar way with its deterministic version in Equation (36) the system Equation (43) restricts to a stochastic Hamiltonian system on each affine coadjoint orbits Equation (32) with respect to the Kirillov-Kostant-Souriau symplectic structure with cocycle Equation (33). From Corollary 8 it follows that the entropy *s* of the Souriau model is preserved by the stochastic dynamics Equation (43).

In absence of the cocycle, Equation (43) can be formally obtained from the variational principle

(44) $$\delta \int_{0}^{T}\left[\langle \mu ,dgg^{-1}\rangle -h\left(\mu \right)dt-\sum_{i=1}^{N}h_{i}\left(\mu \right)\circ dW_{i}\left(t\right)\right]=0,$$

for variations δg and δμ of $\left(g,\mu \right)\in G\times g^{*}$. More precisely, the variations δg and δμ give the two conditions

$$d\mu +{\mathrm{ad}}_{dgg^{-1}}^{*}\mu =0\;\mathrm{and}\;dgg^{-1}=\frac{\delta h}{\delta \mu }dt+\sum_{i=1}^{N}\frac{\delta h_{i}}{\delta \mu }\circ dW_{i}\left(t\right)$$

which yield Equation (43) in the special case Θ=0. Such variational principles play an essential role in stochastic geometric modelling [43,44,45], where the emphasis is made on the Lagrangian side. For instance in [44] the Lagrangian version of Equation (44) given by

(45) $$\delta \int_{0}^{T}\left[\ell \left(\xi \right)dt+\langle \mu ,dgg^{-1}-\xi dt\rangle -\sum_{i=1}^{N}h_{i}\left(\mu \right)\circ dW_{i}\left(t\right)\right]=0,$$

is used, for variations δg, δξ, δμ of $\left(g,\xi ,\mu \right)\in G\times g\times g^{*}$, where ℓ:g→R is the Lagrangian associated with $h:g^{*}\to R$ in the hyperregular case. Variations δg, δξ, and δμ give the three conditions

$$d\mu +{\mathrm{ad}}_{dgg^{-1}}^{*}\mu =0,\;\frac{\delta \ell }{\delta \xi }=\mu ,\;\mathrm{and}\;dgg^{-1}=\xi dt+\sum_{i=1}^{N}\frac{\delta h_{i}}{\delta \mu }\circ dW_{i}\left(t\right)$$

which yield equivalent equations to Equation (43) with Θ=0.

To extend Equation (44) to cover the case of a Lie algebra two-cocycle Θ≠0 we shall formulate the variational principle on the central extension $\hat{G}=G\times R$ of the Lie group *G* with respect to a group two-cocyce B:G×G→R that integrates Θ:g×g→R. We refer to Section 4.2 below for a quick review of the main formulas for central extensions and their use in connection with the Lie-Poisson equations with cocycle. For the application to Equation (43) here, we just need to recall the expression of the group multiplication (g,α)(h,β)=(gh,α+β+B(g,h)) for $\left(g,\alpha \right),\left(h,\beta \right)\in \hat{G}$ where B:G×G→R is the group two-cocycle, and the relation

(46) $$\Theta \left(\xi ,\eta \right)={\left.\frac{d}{ds}\right|}_{s=0}{\left.\frac{d}{dt}\right|}_{t=0}\left(B\left(\exp{\left(t\xi \right)}^{-1},\exp\left(s\eta \right)\right)-B\left(\exp\left(s\eta \right),\exp{\left(t\xi \right)}^{-1}\right)\right)$$

between Θ and *B*. Based on this, we consider the variational principle

$$\delta \int_{0}^{T}\left[\langle \left(\mu ,a\right),\left(dg,d\alpha \right){\left(g,\alpha \right)}^{-1}\rangle -h\left(\mu \right)dt-\sum_{i=1}^{N}h_{i}\left(\mu \right)\circ dW_{i}\left(t\right)\right]=0,$$

for variations δg, δα, δμ, and δa of $\left(g,\alpha \right)\in \hat{G}=G\times R$ and $\left(\mu ,a\right)\in {\hat{g}}^{*}=g^{*}\times R$. In the first term, the operations are associated with the tangent lift of the multiplication on the central extension $\hat{G}$ and the pairing is $\langle (\mu ,a),(\xi ,u)\rangle =\langle \mu ,\xi \rangle +au$, so that we have $\langle \left(\mu ,a\right),\left(dg,d\alpha \right){\left(g,\alpha \right)}^{-1}\rangle =\langle \mu ,dgg^{-1}\rangle +a\left(d\alpha +D_{1}B\left(g,g^{-1}\right)\cdot dg\right)$. A computation, using the fact that *B* integrates Θ, i.e., Equation (46), shows that the variations δg, δα, δμ, and δa give the four conditions

$$\begin{array}{cccc} \; & d\mu +{\mathrm{ad}}_{dgg^{-1}}^{*}\mu -a\Theta \left(dgg^{-1},\cdot \right)=0, & \; & da=0,\\ \; & dgg^{-1}=\frac{\delta h}{\delta \mu }dt+\sum_{i=1}^{N}\frac{\delta h_{i}}{\delta \mu }\circ dW_{i}\left(t\right), & \; & d\alpha +D_{1}B\left(g,g^{-1}\right)\cdot dg=0. \end{array}$$

One notes that taking the initial condition a=1, we get the stochastic Lie-Poisson system with cocycle Equation (43) as desired. This approach using central extension can be easily used on the Lagrangian side too and yields the appropriate extension of Equation (45) to handle a cocycle Θ≠0.

We refer to [43,44,45,46], for various finite and infinite dimensional applications of the stochastic Lie-Poisson Hamiltonian system Equation (42) in the case Θ=0.

### 2.4. Polysymplectic Model of Statistical Mechanics

Polysymplectic geometry, as developed in [47], arises as a special case of multisymplectic geometry which is the natural geometric setting of classical field theories, see, e.g., [48]. When used in conjunction with the general setting developed in Section 2.1 and Section 2.2, the polysymplectic setting furnishes a natural generalisation of the Souriau symplectic model, to which many properties extend. This extension was proposed in [5]. Here we emphazise this model as a specific case of the general framework described in Section 2.1 and Section 2.2. This allows transposing immediately all the properties of this framework to the polysymplectic model. In particular, we will see that the entropy of the polysymplectic model enjoys a natural extension of the Casimir property observed in Section 2.3.2. The relevant equation is here an *Lie-Poisson field equation with cocycle* that we will describe in detail below.

This model is motivated by higher-order model of statistical physics. For instance, for small data analytics (rarified gases, sparse statistical surveys, …), the density of maximum entropy should consider higher order moments constraints, so that the Gibbs density is not only defined by first moment but fluctuations request 2nd order and higher moments, as introduced in [49,50,51,52,53,54].

**Polysymplectic manifolds.** We only need a restricted amount of notions from polysymplectic geometry which are straighforward extensions of those recalled above in the symplectic context. We refer to [47] for more information. A polysymplectic manifold (M,ω) is a manifold *M* endowed with a closed nondegenerate $R^{n}$-valued 2-form. We can identify ω with a collection $\left(\omega^{1},\ldots ,\omega^{n}\right)$, of closed 2-forms with ${⋂}_{i=1}^{n}\ker\omega^{i}=\left\{0\right\}$.

A Lie group action ϕ:G×M→M of *G* on *M* is polysymplectic, if $\Phi_{g}^{*}\omega^{i}=\omega^{i}$, for every g∈G and i=1,…,n. Similarly as before, this implies that $i_{\xi_{M}}\omega$ is a closed $R^{n}$-valued one-form on *M*. If this form is exact, then the action is called *Hamiltonian* and admits a *polysymplectic momentum map* $J:M\to L(g,R^{n})$, which satisfies

$$i_{\xi_{M}}\omega =dJ_{\xi },$$

where $J_{\xi }:M\to R^{n}$ is defined by $J_{\xi }\left(m\right)=J\left(m\right)\cdot \xi$. In a similar way with the symplectic case, if *M* is connected, there is group one-cocycle $\theta \in C^{\infty }\left(G,L\left(g,R^{n}\right)\right)$, $\theta =(\theta^{1},\ldots ,\theta^{n})$, defined by

$$\theta^{i}\left(g\right)=J^{i}\left(\Phi_{g}\left(m\right)\right)-{\mathrm{Ad}}_{g^{-1}}^{*}\left(J^{i}\left(m\right)\right).$$

and one defines the map $\Theta :g\times g\to R^{n}$ by

(47) $$\Theta \left(\xi ,\eta \right):={\left.\frac{d}{d\varepsilon }\right|}_{\varepsilon =0}\theta \left(\exp\left(\varepsilon \xi \right)\right)\left(\eta \right)\in R^{n}.$$

Taking the derivative of the relation above, we get

(48) $$\Theta {\left(\xi ,\eta \right)}^{i}=J_{\left[\xi ,\eta \right]}^{i}-\omega^{i}\left(\xi_{M},\eta_{M}\right).$$

As a consequence Θ is skew-symmetric, and satisfies the two-cocycle identity

(49) Θ([ξ,η],ζ)+Θ([η,ζ],ξ)+Θ([ζ,ξ],η)=0,

see [47].

**Polysymplectic model.** The *polysymplectic model* of statistical mechanics is obtained by considering the following specific situation in the equivariant setting described in Section 2.2:

$$\begin{array}{cc} M & :\;a\;\mathrm{polysymplectic}\;\mathrm{manifold}\\ d\mu & :\;a\;\mathrm{volume}\;\mathrm{form}\\ \phi_{g} & :\;a\;\mathrm{volume}\;\mathrm{preserving}\;\mathrm{Hamiltonian}\;\mathrm{action}\\ E=L(R^{n},g) & :\;\mathrm{the}\;\mathrm{linear}\;\mathrm{maps}\;\mathrm{from}\;R^{n}\;\mathrm{to}\;\mathrm{the}\;\mathrm{Lie}\;\mathrm{algebra}\;\mathrm{of}\;G\\ \rho_{g}={\left({\mathrm{Ad}}_{g}\right)}^{n} & :\;\mathrm{the}\;\mathrm{action}\;\mathrm{induced}\;\mathrm{on}\;L(R^{n},g)\;\mathrm{by}\;\mathrm{the}\;\mathrm{adjoint}\\ \; & \;\mathrm{action}\;\mathrm{of}\;G\;\mathrm{on}\;g\\ U=J:M\to E^{*}=L\left(g,R^{n}\right) & :\;a\;\mathrm{polysymplectic}\;\mathrm{momentum}\;\mathrm{map}. \end{array}$$

Here the space $E=L(R^{n},g)$ of linear maps is identified with the Cartesian product $g^{n}=g\times \ldots \times g$ and ${\left({\mathrm{Ad}}_{g}\right)}^{n}$ acts on β∈E as

$${\left({\mathrm{Ad}}_{g}\right)}^{n}\left(\beta_{1},\ldots ,\beta_{n}\right)=\left({\mathrm{Ad}}_{g}\beta_{1},\ldots ,{\mathrm{Ad}}_{b}\beta_{n}\right).$$

The thermodynamic heat becomes a map $Q:\Omega \subset L\left(R^{n},g\right)\to \Omega^{*}\subset L\left(g,R^{n}\right)$ with $Q\left(\beta \right)=E_{\beta }\left(J\right)\in \Omega^{*}\subset L\left(g,R^{n}\right)$ and the Fisher metric on $\Omega \subset L(R^{n},g)$ is

$$I\left(\beta \right)=E_{\beta }\left(\left(J-E_{\beta }\left(J\right)\right)⊗\left(J-E_{\beta }\left(J\right)\right)\right)\in \mathrm{sym}\left(L(R^{n},g)\right).$$

Proposition 5 directly yields the following equivariance properties

$${\left({\mathrm{Ad}}_{g}\right)}^{n}\Omega =\Omega ,\;\psi \left({\left({\mathrm{Ad}}_{g}\right)}^{n}\beta \right)=\psi \left(\beta \right)e^{\langle \theta (g^{-1}),\beta \rangle },\;p_{\beta }\circ \phi_{g}=p_{{\left({\mathrm{Ad}}_{g^{-1}}\right)}^{n}\beta }$$

and

(50) $$\begin{array}{cc} \; & \Phi \left({\left({\mathrm{Ad}}_{g}\right)}^{n}\beta \right)=\Phi \left(\beta \right)-\langle \theta (g^{-1}),\beta \rangle \end{array}$$

(51) $$\begin{array}{cc} \; & Q\left({\left({\mathrm{Ad}}_{g}\right)}^{n}\beta \right)={\left({\mathrm{Ad}}_{g^{-1}}^{*}\right)}^{n}\left(Q\left(\beta \right)\right)+\theta \left(g\right) \end{array}$$

(52) $$\begin{array}{cc} \; & s\left({\left({\mathrm{Ad}}_{g^{-1}}^{*}\right)}^{n}\nu +\theta \left(g\right)\right)=s\left(\nu \right) \end{array}$$

(53) $$\begin{array}{cc} \; & K\left({\left({\mathrm{Ad}}_{g}\right)}^{n}\beta \right)\left({\left({\mathrm{Ad}}_{g}\right)}^{n}\delta \beta_{1},{\left({\mathrm{Ad}}_{g}\right)}^{n}\delta \beta_{2}\right)=K\left(\beta \right)\left(\delta \beta_{1},\delta \beta_{2}\right), \end{array}$$

for every g∈G. Note also that $\Omega^{*}$ is invariant under the affine action $\nu \in L\left(g,R^{n}\right)\mapsto {\left({\mathrm{Ad}}_{g^{-1}}^{*}\right)}^{n}\nu +\theta \left(g\right)\in L\left(g,R^{n}\right)$.

From Proposition 4, given $\nu \in \Omega^{*}\subset L\left(g,R^{n}\right)$, the generalized Gibbs probability density

$$p_{\beta }\left(m\right)=\frac{1}{\psi (\beta )}e^{-\langle J(m),\beta \rangle },$$

with $\beta =Q^{-1}\left(\nu \right)$, satisfies the maximum entropy principle

$$\max_{q}\left[-\int_{M}q\log q\;d\mu \right]\;\mathrm{such}\;\mathrm{that}\;\left\{\begin{array}{c} \;\int_{M}q\;d\mu =1\\ \int_{M}J\;q\;d\mu =\nu . \end{array}\right.$$

**Particular cases.** A particularly simple case of polysymplectic Souriau model is given by the manifold $M=T^{*}Q⊕\ldots ⊕T^{*}Q$ (Whitney sum with *n* factors) endowed with the polysymplectic form $\Omega =(\Omega^{1},\ldots ,\Omega^{n})$, with $\Omega^{k}={\left(\pi^{k}\right)}^{*}\Omega_{\mathrm{can}}$. Here $\pi^{k}:T^{*}Q⊕\ldots ⊕T^{*}Q\to T^{*}Q$ is the projection onto the $k^{th}$ factor of the sum and $\Omega_{\mathrm{can}}$ is the canonical symplectic form on $T^{*}Q$. Let *G* be a Lie group acting on the left on *Q*. Then its naturally induced action on $T^{*}Q⊕\ldots ⊕T^{*}Q$ is polysymplectic and admits the polysymplectic momentum map $J:T^{*}Q⊕\ldots ⊕T^{*}Q\to L\left(g,R^{n}\right)$ given by

$$J\left(\alpha_{q}^{1},\ldots ,\alpha_{q}^{n}\right)=\left(J\left(\alpha_{q}^{1}\right),\ldots ,J\left(\alpha_{q}^{n}\right)\right),$$

where $J:T^{*}Q\to g^{*}$ is the momentum map associated with the cotangent lifted action of *G* on $T^{*}Q$ given in Equation (38). In this case, there is no cocycle.

Another case without cocycle in the polysymplectic momentum map is when *M* is chosen as an orbit $M=O=\{{\left({\mathrm{Ad}}_{g^{-1}}^{*}\right)}^{n}\mu +\theta \left(g\right)|g\in G\}$, $\mu \in L(g,R^{n})$, of the affine left action of *G* on $L(g,R^{n})$ given by $\mu \mapsto {\left({\mathrm{Ad}}_{g^{-1}}^{*}\right)}^{n}\mu +\theta \left(g\right)$, with $\theta \in C^{\infty }\left(G,L\left(g,R^{n}\right)\right)$ a group one-cocycle. This orbit *M* is endowed with a natural polysymplectic form $\omega =(\omega^{1},\ldots ,\omega^{n})$ with $\omega^{i}$ defined by

$$\omega^{i}\left(\mu \right)\left({\mathrm{ad}}_{\xi }^{*}\mu -\Theta \left(\xi ,\cdot \right),{\mathrm{ad}}_{\eta }^{*}\mu -\Theta \left(\eta ,\cdot \right)\right)=\langle \mu^{i},\left[\xi ,\eta \right]\rangle -\Theta^{i}\left(\xi ,\eta \right)$$

where Θ is given in Equation (47), which is the polysymplectic version of Equation (33). In this case, the polysymplectic momentum map is simply the inclusion $J:O\to L(g,R^{n})$ of the orbit in $L(g,R^{n})$.

**Property of the entropy and polysymplectic Lie-Poisson equations with cocycle.** In the context of the polysymplectic model, a natural generalisation of the Lie-Poisson equations with cocycle Equation (36) are

(54) $$\sum_{k=1}^{n}\frac{\partial }{\partial x^{k}}\mu^{k}+\sum_{k=1}^{n}{\mathrm{ad}}_{\frac{\delta h}{\delta \mu^{k}}}^{*}\mu^{k}=\sum_{k=1}^{n}\Theta^{k}\left(\frac{\delta h}{\delta \mu^{k}},\cdot \right),$$

for a map $\mu :x=\left(x^{1},\ldots ,x^{n}\right)\in U\subset R^{n}\mapsto \mu \left(x\right)=\left(\mu^{1}\left(x\right),\ldots ,\mu^{n}\left(x\right)\right)\in L\left(g,R^{n}\right)$, with $h:L(g,R^{n})\to R$ a given Hamiltonian. In absence of the cocycle, such a field theoretic version of the Lie-Poisson equation appears, for instance, for the spacetime Lagrangian and Hamiltonian theoretic description of Cosserat rods and molecular strands, see [36,37].

From the invariance property Equation (52), we have

$$\sum_{k=1}^{n}\langle \frac{\delta s}{\delta \mu^{k}},-{\mathrm{ad}}_{\xi }^{*}\mu^{k}+\Theta^{k}\left(\xi ,\cdot \right)\rangle =0$$

for all ξ∈g. This is equivalent to

$$\sum_{k=1}^{n}{\mathrm{ad}}_{\frac{\delta s}{\delta \mu^{k}}}^{*}\mu^{k}-\sum_{k=1}^{n}\Theta^{i}\left(\frac{\delta s}{\delta \mu^{k}},\cdot \right)=0.$$

For h=s, Equation (54) thus reduce to $\sum_{k=1}^{n}\frac{\partial }{\partial x^{k}}\mu^{k}=0$. This is the natural extension of the Casimir property of *s* observed in the Souriau model in Section 2.3.2, given there by the condition ${\mathrm{ad}}_{\frac{\delta s}{\delta \mu }}^{*}\mu -\Theta \left(\frac{\delta s}{\delta \mu },\cdot \right)=0$, giving $\frac{d}{dt}\mu =0$.

### 2.5. The Fisher Metric on Orbits and Equivariance

We give here a general expression of the Fisher metric on orbits of the action ρ:G×E→E, in the general setting described in Section 2.1 and Section 2.2. This clarifies the link between the Fisher metric and the metric on adjoint orbits considered by Souriau, as enlightened in [4].

As in Section 2.1 we consider a manifold *M*, a vector space *E*, a function $U:M\to E^{*}$, and the class of generalized Gibbs probability densities

$$p_{\beta }\left(m\right)=\frac{1}{\psi (\beta )}e^{-\langle U(m),\beta \rangle },\;\beta \in \Omega .$$

As in Section 2.2 given a Lie group *G* we consider an action ϕ:G×M→M and a representation ρ:G×E→E. We denote by

$$\xi_{E}\left(\beta \right):={\left.\frac{d}{d\varepsilon }\right|}_{\varepsilon =0}\rho_{\exp(\epsilon \xi )}\left(\beta \right)\;\mathrm{and}\;\xi_{E^{*}}\left(\nu \right):={\left.\frac{d}{d\varepsilon }\right|}_{\varepsilon =0}\rho_{\exp(\varepsilon \xi )}^{*}\left(\nu \right),$$

β∈E, $\nu \in E^{*}$ the infinitesimal generators of the representations $\rho_{g}$ and $\rho_{g}^{*}$ associated with ξ∈g. We will use the equality $\langle \xi_{E^{*}}\left(\nu \right),\beta \rangle =\langle \nu ,\xi_{E}\left(\beta \right)\rangle$. Given the group one-cocycle $\theta \in C^{\infty }\left(G,E^{*}\right)$ associated with the function *U*, see Equation (16), we define $\Theta \in C^{\infty }\left(g,E^{*}\right)$ by

(55) $$\langle \Theta (\xi ),\beta \rangle ={\left.\frac{d}{d\varepsilon }\right|}_{\varepsilon =0}\langle \theta (\exp(\varepsilon \xi )),\beta \rangle ,$$

for ξ∈g and β∈E. Recall that the Fisher metric is $I\left(\beta \right)=-E_{\beta }\left[D^{2}\log p_{\beta }\right]$ and coincides with the generalized heat capacity, see Proposition 2.

**Proposition** **9.** *On the G-orbit through*β∈Ω*, the Fisher metric is written in terms of* Θ *and Q as follows*

(56) $$I\left(\beta \right)\left(\xi_{E}\left(\beta \right),\zeta_{E}\left(\beta \right)\right)=-\langle \Theta \left(\xi \right),\zeta_{E}\left(\beta \right)\rangle +\langle \xi_{E^{*}}\left(Q\left(\beta \right)\right),\zeta_{E}\left(\beta \right)\rangle .$$

**Proof.**  Taking the derivative with respect to *g* at *e* of the equality Equation (18) given by

$$\langle Q(\rho_{g}\left(\beta \right)),\gamma \rangle =\langle \rho_{g^{-1}}^{*}\left(Q\left(\beta \right)\right),\gamma \rangle +\langle \theta (g),\gamma \rangle$$

for every γ∈E, we get

$$\langle DQ\left(\beta \right)\cdot \xi_{E}\left(\beta \right),\gamma \rangle =\langle \Theta (\xi ),\gamma \rangle -\langle \xi_{E^{*}}\left(Q\left(\beta \right)\right),\gamma \rangle ,$$

for every ξ∈g. For $\gamma =\zeta_{E}\left(\beta \right)\in T_{\beta }O$, we get

$$\langle DQ\left(\beta \right)\cdot \xi_{E}\left(\beta \right),\zeta_{E}\left(\beta \right)\rangle =\langle \Theta \left(\xi \right),\zeta_{E}\left(\beta \right)\rangle -\langle \xi_{E^{*}}\left(Q\left(\beta \right)\right),\zeta_{E}\left(\beta \right)\rangle .$$

Therefore, from Proposition 2, we can write

$$I\left(\beta \right)\left(\xi_{E}\left(\beta \right),\zeta_{E}\left(\beta \right)\right)=-D^{2}\Phi \left(\beta \right)\left(\xi_{E}\left(\beta \right),\zeta_{E}\left(\beta \right)\right)=-\langle \Theta \left(\xi \right),\zeta_{E}\left(\beta \right)\rangle +\langle \xi_{E^{*}}\left(Q\left(\beta \right)\right),\zeta_{E}\left(\beta \right)\rangle ,$$

which proves the result. ☐

We illustrate this result for the Souriau model, its polysymplectic extension, and the Koszul model.

**Souriau Lie group statistical model.** In this case, *M* is endowed with a symplectic structure ω, we take E=g and $U=J:M\to g^{*}$ with nonequivariance cocycle $\theta \in C^{\infty }\left(G,g^{*}\right)$, i.e., $\theta \left(g\right)=J\left(\phi_{g}\left(m\right)\right)-{\mathrm{Ad}}_{g^{-1}}^{*}J\left(m\right)\in g^{*}$. The map Θ defined in Equation (55) becomes here a two cocycle Θ:g×g→R, see Equations (25)–(27), via the relation $\langle \Theta (\xi ),\eta \rangle =\Theta \left(\xi ,\eta \right)$. Proposition 9 immediately yields the following result as a corollary, which is obtained by noting that $\xi_{E}\left(\beta \right)={\mathrm{ad}}_{\xi }\beta$ and $\xi_{E^{*}}\left(\nu \right)={\mathrm{ad}}_{\xi }^{*}\nu$ and is a consequence of Equation (29).

**Corollary** **10.** *On the adjoint orbit through β in* g *, the Fisher metric is written as follows*

(57) $$I\left(\beta \right)\left({\mathrm{ad}}_{\xi }\beta ,{\mathrm{ad}}_{\zeta }\beta \right)=-\Theta \left(\xi ,{\mathrm{ad}}_{\zeta }\beta \right)+\langle {\mathrm{ad}}_{\xi }^{*}Q\left(\beta \right),{\mathrm{ad}}_{\zeta }\beta \rangle .$$

Please note that Equation (57) can be written as

$$I\left(\beta \right)\left({\mathrm{ad}}_{\xi }\beta ,{\mathrm{ad}}_{\zeta }\beta \right)=-\Theta_{\beta }\left(\xi ,{\mathrm{ad}}_{\zeta }\beta \right),$$

where $\Theta_{\beta }\left(\xi ,\eta \right):=\Theta \left(\xi ,\eta \right)-\langle {\mathrm{ad}}_{\xi }^{*}Q\left(\beta \right),\eta \rangle =\Theta \left(\xi ,\eta \right)-\langle Q(\beta ),[\xi ,\eta ]\rangle$ is a two-cocycle. In particular, the last term is a coboundary. We refer to [2] for more information.

**Polysymplectic Lie group statistical model.** In this case, *M* is endowed with a polysymplectic structure $\omega =(\omega^{1},\ldots ,\omega^{n})$, we take $E=L(R^{n},g)$ and $U=J:M\to L(g,R^{n})$ with nonequivariance cocycle $\theta \in C^{\infty }\left(G,L\left(g,R^{n}\right)\right)$:

$$\theta \left(g\right)=J\left(\phi_{g}\left(m\right)\right)-{\left({\mathrm{Ad}}_{g^{-1}}^{*}\right)}^{n}J\left(m\right)\in L\left(g,R^{n}\right).$$

The map $\Theta \in C^{\infty }\left(g,L\left(g,R^{n}\right)\right)$ defined in Equation (55) is identified here with the map $\Theta :g\times g\to R^{n}$ defined in Equation (47), with the properties Equations (48) and (49). The identification being Θ(ξ)(η)=Θ(ξ,η), where $\Theta \left(\xi \right)\in L\left(g,R^{n}\right)$ is applied to η∈g. We note the equality $\langle \Theta \left(\xi \right),\left(\eta_{1},\ldots ,\eta_{n}\right)\rangle =\sum_{i=1}^{n}\Theta^{i}\left(\xi ,\eta_{i}\right)$, for $\left(\eta_{1},\ldots ,\eta_{n}\right)\in g^{n}$ identified with $L(R^{n},g)$, where $\langle \;,\rangle$ on the left hand side is the duality pairing between $L(R^{n},g)$ and $L(g,R^{n})$.

We now apply Proposition 9, which follows here from Equation (51). We have the infinitesimal generators $\xi_{E}\left(\beta \right)=\left({\mathrm{ad}}_{\xi }\beta_{1},\ldots ,{\mathrm{ad}}_{\xi }\beta_{n}\right)$ and $\xi_{E^{*}}\left(\nu \right)=\left({\mathrm{ad}}_{\xi }^{*}\nu^{1},\ldots ,{\mathrm{ad}}_{\xi }^{*}\nu^{n}\right)$, and we get

$$\langle \Theta \left(\xi \right),\zeta_{E}\left(\beta_{1},\ldots ,\beta_{n}\right)\rangle =\langle \Theta \left(\xi \right),\left({\mathrm{ad}}_{\zeta }\beta_{1},\ldots ,{\mathrm{ad}}_{\zeta }\beta_{n}\right)\rangle =\langle \Theta^{i}\left(\xi ,{\mathrm{ad}}_{\zeta }\beta_{i}\right)\rangle$$

and

$$\langle \xi_{E^{*}}Q\left(\beta \right),\zeta_{E}\left(\beta \right)\rangle =\sum_{i}\langle {\mathrm{ad}}_{\xi }^{*}Q{\left(\beta \right)}^{i},{\mathrm{ad}}_{\zeta }\beta_{i}\rangle .$$

Therefore, the following result is obtained.

**Corollary** **11.** *On the adjoint orbit through β in* $L(R^{n},g)$ *, the Fisher metric is written as follows*

(58) $$\begin{array}{c} I\left(\beta \right)\left(\left({\mathrm{ad}}_{\xi }\beta_{1},\ldots ,{\mathrm{ad}}_{\xi }\beta_{n}\right),\left({\mathrm{ad}}_{\zeta }\beta_{1},\ldots ,{\mathrm{ad}}_{\zeta }\beta_{n}\right)\right)=-\sum_{i}\Theta^{i}\left(\xi ,{\mathrm{ad}}_{\zeta }\beta_{i}\right)+\sum_{i}\langle {\mathrm{ad}}_{\xi }^{*}Q{\left(\beta \right)}^{i},{\mathrm{ad}}_{\zeta }\beta_{i}\rangle . \end{array}$$

**Koszul model.** For the Koszul model with $\Omega =\mathrm{sym}{\left(n\right)}^{+}$ the cone of positive definite matrices and the Lie group G=GL(n), the actions Equations (21) and (22) have the associated infinitesimal generators

$$\xi_{E}\left(\beta \right)=-\xi^{T}\beta -\beta \xi \;\mathrm{and}\;\xi_{E^{*}}\left(\nu \right)=-\xi \nu -\nu \xi^{T}.$$

In this case, Θ=0 and Proposition 9 is satisfied by noting the equalities

$$I\left(\beta \right)\left(\xi_{E}\left(\beta \right),\zeta_{E}\left(\beta \right)\right)=\left(n+1\right)\mathrm{Tr}\left(\xi \zeta +\beta^{-1}\xi^{T}\beta \zeta \right)=\langle \xi_{E^{*}}\left(Q\left(\beta \right)\right),\zeta_{E}\left(\beta \right)\rangle .$$

## 3. Applications

In this section, we show how the framework considered in Section 2 applies to various examples and helps identifying common underlying geometric structures. We start with the case of multivariate Gaussian probability densities as an illustration of the general framework, for which a cocycle is needed and which does not fall into the setting of the Souriau model. We then enlighten the strong analogies with quantum information geometry by considering Lie algebras with unitary representation and show that the Fisher metric as defined from the generalized heat capacity in Section 2.1, coincides with the Bogoliubov-Kubo-Mori metric. In this particular case the equation with Casimir dissipation/production considered in Section 2.3.3 reproduces a dissipative model of [25]. Finally, we consider in detail the case of the Euclidean group of the plane SE(2) since it allows explicit and relatively easy computations while exhibiting the interesting feature of having cocycle. This example fits into the setting of the Souriau symplectic model.

### 3.1. Multivariate Gaussian Probability Densities

In this paragraph we study in detail the case of multivariate Gaussian densities, following the approach developed in Section 2.1 and Section 2.2. A first treatment in this spirit was given in [4], Section 8. Here we clarify several steps in this approach by following systematically the general setting presented in Section 2.1 and Section 2.2, while we note that this example is not a particular case of the Souriau model. We present explicitly the cocycle, which is here defined on the general affine group, with values in the Cartesian product of symmetric matrices and the Euclidean space.

**Gaussian probability densities in generalized Gibbs form.** Consider a multivariate Gaussian density with symmetric and positive definite covariance matrix $R\in {\mathrm{sym}}^{+}\left(n\right)$ and mean $m\in R^{n}$. The Gaussian probability density is written in the generalized Gibbs form $p_{\beta }$ discussed above in Section 2.1 as follows:

$$\begin{array}{cc} p_{\left(R,m\right)}\left(z\right) & =\frac{1}{{\left(2\pi \right)}^{n/2}\det{\left(R\right)}^{1/2}}e^{-\frac{1}{2}{\left(z-m\right)}^{T}R^{-1}\left(z-m\right)}\\ \; & =\frac{1}{{\left(2\pi \right)}^{n/2}\det{\left(R\right)}^{1/2}e^{\frac{1}{2}m^{T}R^{-1}m}}e^{-\left(\frac{1}{2}z^{T}R^{-1}z-m^{T}R^{-1}z\right)}\\ \; & =\frac{1}{{\left(2\pi \right)}^{n/2}\det{\left(R\right)}^{1/2}e^{\frac{1}{2}m^{T}R^{-1}m}}e^{-\langle \left(zz^{T},z\right),\left(\frac{1}{2}R^{-1},-R^{-1}m\right)\rangle }\\ \; & =:\frac{1}{\psi (\beta )}e^{-\langle U(z),\beta \rangle }=:p_{\beta }\left(z\right), \end{array}$$

for every $z\in R^{n}$. In the last equality above, we have defined the energy function

$$U:R^{n}\to \mathrm{sym}\left(n\right)\times R^{n},\;U\left(z\right)=\left(zz^{T},z\right),$$

the vector $\beta \in {\mathrm{sym}}^{+}\left(n\right)\times R^{n}$ in terms of (R,m) as

(59) $$\beta =\left(\beta_{1},\beta_{2}\right):=\left(\frac{1}{2}R^{-1},-R^{-1}m\right)\;\Leftrightarrow \;R=\frac{1}{2}\beta_{1}^{-1},\;m=-\frac{1}{2}\beta_{1}^{-1}\beta_{2},$$

and the partition function

$$\begin{array}{cc} \psi (\beta ) & ={\left(2\pi \right)}^{n/2}\det{\left(R\right)}^{1/2}e^{\frac{1}{2}m^{T}R^{-1}m}=\pi^{n/2}\det{\left(\beta_{1}\right)}^{-1/2}e^{\frac{1}{4}\mathrm{Tr}\left(\beta_{2}^{T}\beta_{1}^{-1}\beta_{2}\right)}. \end{array}$$

The general theory of Section 2.1 will be applied here with the manifold $M=R^{n}$, the vector space $E=\mathrm{sym}\left(n\right)\times R^{n}$, and the open subset $\Omega ={\mathrm{sym}}^{+}\left(n\right)\times R^{n}$. It is important to note that the element β of the general theory is not given by the couple (R,m), but related to (R,m) via Equation (59). This plays a main role in the understanding of the equivariance properties below.

**Characteristic function, thermodynamic heat, and entropy.** The Massieu potential is computed in terms of β∈Ω as

$$\begin{array}{cc} \Phi (\beta ) & =-\log\left(\psi \left(\beta \right)\right)=-\frac{n}{2}\log\left(2\pi \right)-\frac{1}{2}\log\left(\det\left(R\right)\right)-\frac{1}{2}m^{T}R^{-1}m\\ \; & =K+\frac{1}{2}\log\left(\det\left(\beta_{1}\right)\right)-\frac{1}{4}\beta_{2}^{T}\beta_{1}^{-1}\beta_{2}, \end{array}$$

where we defined the constant $K=-\frac{n}{2}\log\left(\pi \right)$. To compute the derivative, we consider the dual space $E^{*}=\mathrm{sym}\left(n\right)\times R^{n}$, with duality pairing

$$\langle \left(\nu_{1},\nu_{2}\right),\left(\beta_{1},\beta_{2}\right)\rangle =\mathrm{Tr}\left(\nu_{1}\beta_{1}\right)+\nu_{2}\cdot \beta_{2},$$

$\left(\nu_{1},\nu_{2}\right)\in E^{*}$, $\left(\beta_{1},\beta_{2}\right)\in E^{*}$. With respect to this duality pairing we have

$$\frac{\delta \Phi }{\delta \beta_{1}}=\frac{1}{2}\beta_{1}^{-1}+\frac{1}{4}\beta_{1}^{-1}\beta_{2}{\left(\beta_{1}^{-1}\beta_{2}\right)}^{T}=R+mm^{T},\;\frac{\delta \Phi }{\delta \beta_{2}}=-\frac{1}{2}\beta_{1}^{-1}\beta_{2}=m,$$

so we get the thermodynamic heat $Q:\Omega \subset E\to \Omega^{*}\subset E^{*}$ as

$$\beta =\left(\beta_{1},\beta_{2}\right)\mapsto Q\left(\beta_{1},\beta_{2}\right)=\left(\frac{1}{2}\beta_{1}^{-1}+\frac{1}{4}\beta_{1}^{-1}\beta_{2}{\left(\beta_{1}^{-1}\beta_{2}\right)}^{T},-\frac{1}{2}\beta_{1}^{-1}\beta_{2}\right)=\left(\nu_{1},\nu_{2}\right).$$

In terms of the covariance matrix *R* and the mean *m*, this is written as

$$\left(\frac{1}{2}R^{-1},-R^{-1}m\right)\in \Omega \subset E\mapsto \left(R+mm^{T},m\right)\in \Omega^{*}\subset E^{*}.$$

The entropy in terms of $\beta =(\beta_{1},\beta_{2})$ and (R,m) is computed by taking the Legendre transform of Φ as

$$\begin{array}{cc} s(\beta_{1},\beta_{2}) & =\frac{n}{2}\left(1+\log\pi \right)-\frac{1}{2}\log\left(\det\left(\beta_{1}\right)\right)\\ s(R,m) & =\frac{n}{2}\left(1+\log\left(2\pi \right)\right)+\frac{1}{2}\log\left(\det\left(R\right)\right). \end{array}$$

Its expression $s:\Omega^{*}\to R$ in terms of $\left(\nu_{1},\nu_{2}\right)$ is found by using

$$Q^{-1}\left(\nu_{1},\nu_{2}\right)=\left(\frac{1}{2}{\left(\nu_{1}-\nu_{2}\nu_{2}^{T}\right)}^{-1},-{\left(\nu_{1}-\nu_{2}\nu_{2}^{T}\right)}^{-1}\nu_{2}\right).$$

**Fisher information metric.** We compute the generalized heat capacity $K\left(\beta \right):=-D^{2}\Phi \left(\beta \right)$ as follows, see Section 2.1:

$$\begin{array}{cc} K(\beta ) & =-D^{2}\Phi \left(\beta_{1},\beta_{2}\right)\left(\left(\delta \beta_{1},\delta \beta_{2}\right),\left(\Delta \beta_{1},\Delta \beta_{2}\right)\right)\\ \; & =-{\left.\frac{d}{d\varepsilon }\right|}_{\varepsilon =0}D\Phi \left(\beta_{1}+\varepsilon \Delta \beta_{1},\beta_{2}+\varepsilon \Delta \beta_{2}\right)\left(\delta \beta_{1},\delta \beta_{2}\right)\\ \; & =-{\left.\frac{d}{d\varepsilon }\right|}_{\varepsilon =0}\frac{1}{2}\mathrm{Tr}\left(\beta_{1}^{-1}\delta \beta_{1}\right)-\frac{1}{4}\mathrm{Tr}\left(\beta_{1}^{-1}\beta_{2}\beta_{2}^{T}\beta_{1}^{-1}\delta \beta_{1}\right)+\frac{1}{2}\mathrm{Tr}\left(\beta_{2}^{T}\beta_{1}^{-1}\delta \beta_{2}\right)\\ \; & =\frac{1}{2}\mathrm{Tr}\left(\beta_{1}^{-1}\Delta \beta_{1}\beta_{1}^{-1}\delta \beta_{1}\right)+\frac{1}{2}\mathrm{Tr}\left(\beta_{1}^{-1}\Delta \beta_{1}\beta_{1}^{-1}\beta_{2}\beta_{2}^{T}\beta_{1}^{-1}\delta \beta_{1}\right)\\ \; & \;-\frac{1}{2}\mathrm{Tr}\left(\beta_{1}^{-1}\Delta \beta_{2}\beta_{2}^{T}\beta_{1}^{-1}\delta \beta_{1}\right)-\frac{1}{2}\mathrm{Tr}\left(\beta_{1}^{-1}\delta \beta_{2}\beta_{2}^{T}\beta_{1}^{-1}\Delta \beta_{1}\right)\\ \; & \;+\frac{1}{2}\mathrm{Tr}\left(\Delta \beta_{2}^{T}\beta_{1}^{-1}\delta \beta_{2}\right). \end{array}$$

From Proposition 2 this coincides with the Fisher metric. Let us verify that this is the case by rewriting these five terms in terms of the mean and covariance matrix (m,R). The above expression equals

$$\begin{array}{cc} \; & =\frac{1}{2}\mathrm{Tr}\left(\Delta RR^{-1}\delta RR^{-1}\right)\\ \; & \;+\mathrm{Tr}(R^{-1}\delta RR^{-1}mm^{T}R^{-1}\Delta R)\\ \; & \;-\mathrm{Tr}\left(R^{-1}\delta RR^{-1}mm^{T}R^{-1}\Delta R\right)+\mathrm{Tr}\left(\Delta mm^{T}R^{-1}\delta RR^{-1}\right)\\ \; & \;-\mathrm{Tr}\left(R^{-1}\Delta RR^{-1}mm^{T}R^{-1}\delta R\right)+\mathrm{Tr}\left(\delta mm^{T}R^{-1}\Delta RR^{-1}\right)\\ \; & \;+\mathrm{Tr}\left(R^{-1}\Delta RR^{-1}mm^{T}R^{-1}\delta R\right)-\mathrm{Tr}\left(\delta mm^{T}R^{-1}\Delta RR^{-1}\right)\\ \; & \;\;-\mathrm{Tr}\left(\Delta m^{T}R^{-1}\delta RR^{-1}m\right)+\mathrm{Tr}\left(\Delta m^{T}R^{-1}\delta m\right)\\ \; & =\frac{1}{2}\mathrm{Tr}\left(\Delta RR^{-1}\delta RR^{-1}\right)+\mathrm{Tr}\left(\Delta m^{T}R^{-1}\delta m\right), \end{array}$$

which gives the Fisher metric I(R,m) for multivariate Gaussian densities.

**Equivariance with respect to the general affine group.** We consider the general affine group

$$GA\left(n\right)=GL\left(n\right)\;Ⓢ\;R^{n}$$

defined as the semidirect product of the general linear group and $R^{n}$. The group multiplication is

(A,a)(B,b)=(AB,Ab+a)

and the inverse of an element is ${\left(A,a\right)}^{-1}=\left(A^{-1},-A^{-1}a\right)$. The Lie algebra is the semidirect product Lie algebra $\mathrm{ga}\left(n\right)=\mathrm{gl}\left(n\right)\;Ⓢ\;R^{n}$ with Lie brackets [(U,u),(V,v)]=(UV−VU,Uv−Vu).

The group GA(n) acts on the left on the covariance matrix and the mean $\left(R,m\right)\in {\mathrm{sym}}^{+}\left(n\right)\times R^{n}$ as follows:

(60) $$\Psi_{\left(A,a\right)}\left(R,m\right)=\left(ARA^{T},Am+a\right).$$

We consider the left action of GA(n) on $R^{n}$ given by

$$\phi_{\left(A,a\right)}\left(z\right)=Az+a.$$

We note that $J\phi_{\left(A,a\right)}=\det\left(A\right)$, a constant function on $R^{n}$, hence ϕ satisfies the hypothesis of Lemma 5.

It is instructive to observe that the expression

$$\Phi (\Psi_{\left(A,a\right)}\left(R,m\right))-\Phi \left(R,m\right)$$

is not linear in (R,m), compare with Equation (17). However, such a statement is true when it is expressed in terms of the variables $\left(\beta_{1},\beta_{2}\right)$. We first need the expression of the action of GL(n) on $\left(\beta_{1},\beta_{2}\right)$. This is done in the next lemma.

**Lemma** **12.** *The left action of*GA(n)*induced on*$\left(\beta_{1},\beta_{2}\right)\in \mathrm{sym}\left(n\right)\times R^{n}$*by the action* Ψ *bin Equation (60) is given by*

$$\rho_{\left(A,a\right)}\left(\beta_{1},\beta_{2}\right)=\left(A^{-T}\beta_{1}A^{-1},A^{-T}\beta_{2}-2A^{-T}\beta_{1}A^{-1}a\right).$$

*Its dual left action is*

$$\rho_{{\left(A,a\right)}^{-1}}^{*}\left(\nu_{1},\nu_{2}\right)=\left(A\nu_{1}A^{T}+{\left[2A\nu_{2}a^{T}\right]}^{\mathrm{sym}},A\nu_{2}\right)$$

**Proof.**  This is a direct computation using Equation (59). ☐

The situation is illustrated in the following commuting diagram.

$$\begin{array}{ccc} \left(R,m\right)\in \mathrm{sym}\left(n\right)\times R^{n} & \overset{\Psi_{\left(A,a\right)}}{⟶} & \mathrm{sym}\left(n\right)\times R^{n}\\ ↓ & & ↓\\ \left(\beta_{1},\beta_{2}\right)\in \mathrm{sym}\left(n\right)\times R^{n} & \overset{\rho_{\left(A,a\right)}}{⟶} & \mathrm{sym}\left(n\right)\times R^{n} \end{array}$$

The following result shows that the equivariant setting developed in Section 2.2 applies here with the action $\phi_{\left(A,a\right)}$ and the representation $\rho_{\left(A,a\right)}$ (not $\Psi_{\left(A,a\right)}$).

**Lemma** **13.** *The energy function* $U\left(z\right)=\left(zz^{T},z\right)$ *satisfies the relation*

(61) $$U\left(\phi_{\left(A,a\right)}\left(z\right)\right)=\rho_{{\left(A,a\right)}^{-1}}^{*}\left(U\left(z\right)\right)+\theta \left(A,a\right)$$

*for the group one-cocycle* $\theta :GA\left(n\right)\to \mathrm{sym}\left(n\right)\times R^{n}$ *given by*

$$\theta \left(A,a\right)=\left(aa^{T},a\right).$$

*The Massieu potential, the thermodynamic heat, and the entropy satisfy the equivariance properties*

$$\begin{array}{cc} \; & \Phi \left(\rho_{\left(A,a\right)}\left(\beta_{1},\beta_{2}\right)\right)-\Phi \left(\beta_{1},\beta_{2}\right)=-\log\left(\det\left(A\right)\right)+\langle \theta \left({\left(A,a\right)}^{-1}\right),\left(\beta_{1},\beta_{2}\right)\rangle \\ \; & Q\left(\rho_{\left(A,a\right)}\left(\beta_{1},\beta_{2}\right)\right)=\rho_{{\left(A,a\right)}^{-1}}^{*}\left(Q\left(\beta_{1},\beta_{2}\right)\right)+\theta \left(A,a\right)\\ \; & s\left(\rho_{{\left(A,a\right)}^{-1}}^{*}\left(\nu_{1},\nu_{2}\right)+\theta \left(A,a\right)\right)=s\left(\nu_{1},\nu_{2}\right)+\log\left(\det\left(A\right)\right). \end{array}$$

**Proof.**  To prove Equation (61) we note that

$$\begin{array}{cc} \; & U\left(\phi_{\left(A,a\right)}\left(z\right)\right)-\rho_{{\left(A,a\right)}^{-1}}^{*}\left(U\left(z\right)\right)=U\left(Az+a\right)-\rho_{{\left(A,a\right)}^{-1}}^{*}\left(zz^{T},z\right)\\ \; & \;=\left(\left(Az+a\right){\left(Az+a\right)}^{T},Az+a\right)-\left(Azz^{T}A^{T}+{\left[2Aza^{T}\right]}^{\mathrm{sym}},Az\right)=\left(aa^{T},a\right). \end{array}$$

The other results follow from Proposition 5 and from $J\phi_{\left(A,a\right)}=\det\left(A\right)$. Alternatively, we can compute explicitly

$$\begin{array}{cc} \; & \Phi \left(\psi_{\left(A,a\right)}\left(\beta_{1},\beta_{2}\right)\right)=\Phi \left(A^{-T}\beta_{1}A^{-1},A^{-T}\beta_{2}-2A^{-T}\beta_{1}A^{-1}a\right)\\ \; & =K+\frac{1}{2}\log\left(\det\left(A^{-T}\beta_{1}A^{-1}\right)\right)\\ \; & \;-\frac{1}{4}\mathrm{Tr}\left({\left(A^{-T}\beta_{1}A^{-1}\right)}^{-1}\left(A^{-T}\beta_{2}-2A^{-T}\beta_{1}A^{-1}a\right){\left(A^{-T}\beta_{2}-2A^{-T}\beta_{1}A^{-1}a\right)}^{T}\right)\\ \; & =K+\frac{1}{2}\log\left(\det{\left(A\right)}^{-2}\beta_{1}\right)\\ \; & \;-\frac{1}{4}\mathrm{Tr}\left(\beta_{1}^{-1}\left(\beta_{2}-2\beta_{1}A^{-1}a\right)\left(\beta_{2}^{T}-2a^{T}A^{-T}\beta_{1}\right)\right)\\ \; & =K-\log\left(\det\left(A\right)\right)+\frac{1}{2}\log\left(\det\left(\beta_{1}\right)\right)\\ \; & \;-\frac{1}{4}\mathrm{Tr}\left(\beta_{1}^{-1}\beta_{2}\beta_{2}^{T}\right)+\frac{1}{2}\mathrm{Tr}\left(\beta_{2}a^{T}A^{-T}\right)+\frac{1}{2}\mathrm{Tr}\left(A^{-1}a\beta_{2}^{T}\right)-\mathrm{Tr}\left(A^{-1}aa^{T}A^{-T}\beta_{1}\right)\\ \; & =\Phi \left(\beta_{1},\beta_{2}\right)-\log\left(\det\left(A\right)\right)+\langle \left(-A^{-1}aa^{T}A^{-T},A^{-1}a\right),\left(\beta_{1},\beta_{2}\right)\rangle \end{array}$$

which shows the result since $\left(-A^{-1}aa^{T}A^{-T},A^{-1}a\right)=\theta \left({\left(A,a\right)}^{-1}\right)$. ☐

The identity relating the Fisher information metric, the cocycle, and the thermodynamic heat follows from the general Equation (56) as

$$I\left(\beta \right)\left(\xi_{E}\left(\beta \right),\zeta_{E}\left(\beta \right)\right)=-\langle \Theta \left(\xi \right),\zeta_{E}\left(\beta \right)\rangle +\langle \xi_{E^{*}}\left(Q\left(\beta \right)\right),\zeta_{E}\left(\beta \right)\rangle ,$$

where $\left(\beta_{1},\beta_{2}\right)\in {\mathrm{sym}}^{+}\left(n\right)\times R^{n}$, $\xi =\left(\xi_{1},\xi_{2}\right),\zeta =\left(\zeta_{1},\zeta_{2}\right)\in \mathrm{ga}\left(n\right)$, $\Theta \left(\xi_{1},\xi_{2}\right)=\left(0,\xi_{2}\right)$ and the infinitesimal generators are

$$\begin{array}{cc} \xi_{E}\left(\beta \right) & =\left(-\xi_{1}^{T}\beta_{1}-\beta_{1}\xi_{1},-\xi_{1}^{T}\beta_{2}-2\beta_{1}\xi_{2}\right)\\ \xi_{E^{*}}\left(\nu \right) & =\left(-\xi_{1}\nu_{1}-\nu_{1}\xi_{1}^{T}-2{\left[\nu_{2}\xi_{2}^{T}\right]}^{\mathrm{sym}},-\xi_{1}\nu_{2}\right). \end{array}$$

**Geodesics on multivariate Gaussian densities and Noether theorem.** Let us consider the Lagrangian L:TΩ=Ω×E→R given by the kinetic energy of the Fisher metric

(62) $$L\left(R,\dot{R},m,\dot{m}\right)=\frac{1}{2}\mathrm{Tr}\left({\left(R^{-1}\dot{R}\right)}^{2}\right)+{\dot{m}}^{T}R^{-1}\dot{m}.$$

The associated Euler-Lagrange equations are

(63) $$\left\{\begin{array}{c} \ddot{R}+\dot{m}{\dot{m}}^{T}-\dot{R}R^{-1}\dot{R}=0\\ \ddot{m}-\dot{R}R^{-1}\dot{m}=0. \end{array}\right.$$

In accordance with Proposition 5, see Equation (20), the Fisher metric is invariant with respect to the action of GA(n) on (R,m)∈Ω given in Equation (60). As a consequence, the Lagrangian is invariant under the tangent lifted action of GA(n) on TΩ given by

$$\Phi_{\left(A,a\right)}^{T}\left(R,\dot{R},m,\dot{m}\right)=\left(ARA^{T},A\dot{R}A^{T},Am+a,A\dot{m}\right).$$

From Noether theorem, the corresponding momentum map is conserved. The momentum map $J^{L}:T\Omega \to \mathrm{ga}{\left(n\right)}^{*}$ associated with this Lagrangian and this action is given by

$$J^{L}\left(R,\dot{R},m,\dot{m}\right)=J\left(R,\frac{\partial L}{\partial \dot{R}},m,\frac{\partial L}{\partial \dot{m}}\right)=J\left(R,R^{-1}\dot{R}R^{-1},m,2R^{-1}\dot{m}\right)$$

with $J:T^{*}\Omega \to \mathrm{ga}{\left(n\right)}^{*}$ the momentum map of the cotangent lifted action of GA(n) relative to the canonical symplectic form, see Equation (38). Using the expression of the infinitesimal generator of Ψ given by

$${\left(U,u\right)}_{\Omega }\left(R,m\right)=\left(R,UR+RU^{T},m,Um+u\right),$$

for (U,u)∈ga(n), we get $J\left(R,m,p_{R},p_{m}\right)=\left(2p_{R}R+p_{m}m^{T},p_{m}\right)$, so that

$$J^{L}\left(R,\dot{R},m,\dot{m}\right)=\left(2R^{-1}\dot{R}+2R^{-1}\dot{m}m^{T},2R^{-1}\dot{m}\right).$$

From Noether theorem, we have the conservation laws

$$\left\{\begin{array}{c} R^{-1}\dot{R}+R^{-1}\dot{m}m^{T}=cste\\ R^{-1}\dot{m}=cste. \end{array}\right.$$

We also refer to [55,56].

### 3.2. Unitary Representations and Quantum Fisher Metric

In this paragraph, we highlight the strong analogies between the equivariant setting considered in this paper, and techniques in quantum information geometry, as developed in [25], see also [28]. In particular, when this setting is considered in the quantum context, the Fisher metric, as defined from the derivative of the generalized heat capacity, coincides with the Bogoliubov-Kubo-Mori metric. We also illustrate how the general equations with Casimir dissipation/production considered above reproduce the dissipative model proposed in [25].

In [25] information geometry was studied for some Lie algebras where for certain unitary representations, the statistical manifold of states was defined as convex cone for which the partition function is finite, making reference to Bogoliubov-Kubo-Mori metric. Please note that only the case with zero cohomology for the Lie algebras g=so(3) and g=sl(2,R) was studied.

Let *G* be a Lie group, acting on a complex Hilbert space by a unitary left representation, $U_{g}:H\to H$. We denote by $\beta_{H}$ the associated infinitesimal generator, giving the Lie algebra representation, and consider the self-adjoint operator $i\beta_{H}$. We assume dimH<∞. The following class of density matrices is considered

(64) $$\rho_{\beta }=\frac{1}{\psi (\beta )}\exp\left(-i\beta_{H}\right),$$

for β∈g, with partition function $\psi \left(\beta \right)=\mathrm{Tr}(\exp\left(-i\beta_{H}\right))$. We adopted in Equation (64) a general form for the class of density matrices, which includes the class considered in [25] and reference therein. Please note that Equation (64) is a model of faithful quantum states. Note also that the map $\beta \mapsto \rho_{\beta }$ is not necessarily injective in general. We do not assume this hypothesis for the development below, but it is required in quantum information geometry.

As in Section 2.1, we adopt the following definitions

$$\Phi \left(\beta \right)=-\log\left(\psi \left(\beta \right)\right),\;Q\left(\beta \right)=D\Phi \left(\beta \right),\;K\left(\beta \right)=-D^{2}\Phi \left(\beta \right)$$

corresponding to the Massieu potential, the thermodynamic heat, and the generalized heat capacity. We note that

$$\langle Q(\beta ),\delta \beta \rangle =\mathrm{Tr}\left(\rho_{\beta }i\delta \beta_{H}\right)={\langle i\delta \beta_{H}\rangle }_{\rho_{\beta }},$$

for all δβ∈g, which gives the expectation value of the observable $i\delta \beta_{H}$ in the quantum state $\rho_{\beta }$. A result analogue to Equation (5) in the classical case. The generalized heat capacity is computed as

$$\begin{array}{cc} K\left(\beta \right)\left(\delta \beta_{1},\delta \beta_{2}\right) & =-D^{2}\Phi \left(\beta \right)\left(\delta \beta_{1},\delta \beta_{2}\right)\\ \; & =\mathrm{Tr}\left(\rho_{\beta }i{\left(\delta \beta_{1}\right)}_{H}i{\left(\delta \beta_{2}\right)}_{H}\right)-\mathrm{Tr}\left(\rho_{\beta }i{\left(\delta \beta_{1}\right)}_{H}\right)\mathrm{Tr}\left(\rho_{\beta }i{\left(\delta \beta_{2}\right)}_{H}\right), \end{array}$$

thereby giving the covariance of the observables $i{\left(\delta \beta_{1}\right)}_{H}$ and $i{\left(\delta \beta_{2}\right)}_{H}$ in the quantum state $\rho_{\beta }$. In [25], *K* is called the *Bogoliubov-Kubo-Mori metric* and chosen as the quantum version to the Fisher metric. Such a choice is geometrically natural in view of the result of Proposition 2 which identifies *K* with the Fisher metric in the classical case.

The von Neumann entropy of the density matrix can be expressed in terms of Φ and *Q* as $-\mathrm{Tr}\left(\rho_{\beta }\log\rho_{\beta }\right)=\mathrm{Tr}\left(\rho_{\beta }i\beta_{H}\right)+\log\psi \left(\beta \right)=\langle Q(\beta ),\beta \rangle -\Phi \left(\beta \right)=s\left(\nu \right)$, for $\nu =Q\left(\beta \right)={\langle i{\left(\cdot \right)}_{H}\rangle }_{\beta }\in g^{*}$. This is analogue to the result of Lemma 1 giving the entropy as the Legendre transform of Φ(β), thus giving a quantum version of the Clairaut equation. See also [57] for a link with the Fisher metric.

Using ${\left({\mathrm{Ad}}_{g}\beta \right)}_{H}=U_{g}\beta_{H}U_{g^{-1}}$, we have the following equivariance properties, which are obtained as in Proposition 5,

$$\begin{array}{cc} \psi ({\mathrm{Ad}}_{g}\beta ) & =\psi (\beta )\\ \Phi ({\mathrm{Ad}}_{g}\beta ) & =\Phi (\beta )\\ \rho_{{\mathrm{Ad}}_{g}\beta } & =U_{g}\circ \rho_{\beta }\circ U_{g^{-1}}\\ Q({\mathrm{Ad}}_{g}\beta ) & ={\mathrm{Ad}}_{g^{-1}}^{*}\left(Q\left(\beta \right)\right)\\ s({\mathrm{Ad}}_{g^{-1}}^{*}\nu ) & =s(\nu )\\ K\left({\mathrm{Ad}}_{g}\beta \right)\left({\mathrm{Ad}}_{g}\delta \beta_{1},{\mathrm{Ad}}_{g}\delta \beta_{2}\right) & =K\left(\beta \right)\left(\delta \beta_{1},\delta \beta_{2}\right), \end{array}$$

for every g∈G. In particular, ψ and Φ are constant on adjoint orbits and *s* is a Casimir for the Lie-Poisson bracket on $g^{*}$

(65) $$\left\{f,g\right\}\left(\mu \right)=\langle \mu ,\left[\frac{\delta f}{\delta \mu },\frac{\delta g}{\delta \mu }\right]\rangle ,$$

where we identify $g^{*}$ with g using the duality pairing $\langle \nu ,\beta \rangle =\mathrm{Tr}\left(\nu^{*}\beta \right)$ and view g as a Lie subalgebra of u(H). Please note that with this pairing, we have ${\mathrm{Ad}}_{g^{-1}}^{*}={\mathrm{Ad}}_{g}$ and ${\mathrm{ad}}_{\beta }^{*}\mu =\left[\mu ,\beta \right]$, so adjoint and coadjoint orbits are identified, and the Kirillov-Kostant-Souriau symplectic form on coadjoint orbits becomes

$$\omega_{O}\left(\mu \right)\left({\mathrm{ad}}_{\xi }\mu ,{\mathrm{ad}}_{\eta }\mu \right)=\langle \mu ,[\xi ,\eta ]\rangle .$$

Relation Equation (56) and ${\mathrm{ad}}_{\xi }^{*}\mu =\left[\mu ,\xi \right]$ gives here the following expression of the Bogoliubov-Kubo-Mori metric on (co)adjoint orbits

$$K\left(\mu \right)\left({\mathrm{ad}}_{\xi }\mu ,{\mathrm{ad}}_{\eta }\mu \right)=\langle {\mathrm{ad}}_{\xi }^{*}Q\left(\mu \right),{\mathrm{ad}}_{\eta }\mu \rangle =\langle Q(\mu ),[\xi ,[\eta ,\mu ]]\rangle =\langle \mu ,[[Q(\mu ),\xi ],\eta ]\rangle .$$

**Casimir dissipation/production.** The general equations for Casimir dissipation/production Equation (40) applied here with g⊂u(H), $g^{*}=g$, and $\gamma \left(\nu ,\beta \right)=\langle \nu ,\beta \rangle =\mathrm{Tr}\left(\nu^{*}\beta \right)$, become

(66) $$\frac{d}{dt}f=\left\{f,h\right\}-\Lambda \;\langle \left[\frac{\delta f}{\delta \mu },\frac{\delta k}{\delta \mu }\right],\left[\frac{\delta s}{\delta \mu },\frac{\delta k}{\delta \mu }\right]\rangle$$

for every *f*, with {f,g} the Lie-Poisson bracket Equation (65). Since ${\mathrm{ad}}_{\xi }^{*}\mu =\left[\mu ,\xi \right]$, Equation (41) yield

(67) $$\frac{d}{dt}\mu +\left[\mu ,\frac{\delta h}{\delta \mu }\right]=\Lambda \left[\left[\frac{\delta s}{\delta \mu },\frac{\delta k}{\delta \mu }\right],\frac{\delta k}{\delta \mu }\right].$$

Such equations where proposed in [25] with $h\left(\mu \right)=\langle iH,\mu \rangle$, $k\left(\mu \right)=\langle iT,\mu \rangle$, $s\left(\mu \right)=\frac{1}{2}\langle \mu ,\mu \rangle$, with *T* and *H* two commuting self-adjoint operator [T,H]=0, thereby yielding the system

(68) $$\frac{d}{dt}\mu +i\left[\mu ,H\right]=-\Lambda \left[\left[\mu ,T\right],T\right],$$

with energy conservation and entropy production (Λ<0)

$$\frac{d}{dt}h=0\;\mathrm{and}\;\frac{d}{dt}s=-\Lambda ∥\left[\mu ,T\right]{∥}^{2}.$$

### 3.3. Souriau Symplectic Model for SE(2), Lie-Poisson Equations with Cocycle, and Casimir Dissipation

In this paragraph, we illustrate many aspects of the geometric setting by considering the special Euclidean group of the plane, as it allows explicit and relatively easy computations while having a nonequivariant momentum map. We present the Lie-Poisson equations with cocycle (affine Lie-Poisson equations) with Casimir dissipation/production associated with the entropy of the Souriau symplectic model.

**Momentum map and cocycle.** Consider the special Euclidean group of the plane $SE\left(2\right)=SO\left(2\right)\;Ⓢ\;R^{2}$ with semidirect product group multiplication

$$\left(R_{\varphi },a\right)\left(R_{\psi },b\right)=\left(R_{\varphi }R_{\psi },R_{\varphi }b+a\right),$$

where $R_{\varphi }$ is a rotation of angle φ. It acts on the plane $R^{2}$ as

(69) $$\phi_{\left(\theta ,a\right)}\left(x\right)=R_{\theta }x+a$$

with infinitesimal generator

$${\left(\lambda ,u\right)}_{R^{2}}\left(x\right)=-\lambda Jx+u$$

for $\left(\lambda ,u\right)\in \mathrm{se}\left(2\right)=\mathrm{so}\left(2\right)\;Ⓢ\;R^{2}$, where we identify so(2) with R and with

$$J=\left[\begin{array}{cc} 0 & 1\\ -1 & 0 \end{array}\right].$$

We consider on $R^{2}$ the symplectic form ω(x,y)=x·Jy. It is easy to see that the action (69) is symplectic and admits the momentum map

$$J\left(x\right)=\left(-\frac{1}{2}{\left|x\right|}^{2},Jx\right).$$

This momentum map is not equivariant, with nonequivariance cocycle given by

(70) $$\theta \left(R_{\varphi },a\right)=\left(-\frac{1}{2}{\left|a\right|}^{2},Ja\right).$$

**Gibbs densities, entropy, and Fisher metric.** The generalized Gibbs probability densities are here given on $M=R^{2}$ by

$$p_{\beta }\left(x\right)=\frac{1}{\psi (\beta )}e^{-\langle J(x),\beta \rangle }=\frac{1}{\psi (\beta )}e^{\frac{1}{2}\lambda {\left|x\right|}^{2}-u\cdot Jx},$$

where β=(λ,u)∈Ω⊂se(2), with $\Omega =\left(-\infty ,0\right)\times R^{2}$ and the partition function and Massieu potential are computed to be

$$\psi \left(\beta \right)=-\frac{2\pi }{\lambda }e^{-\frac{1}{2\lambda }{\left|u\right|}^{2}},\;\Phi \left(\beta \right)=-\log\left(2\pi \right)+\log\left(-\lambda \right)+\frac{1}{2\lambda }{\left|u\right|}^{2},\;\beta =\left(\lambda ,u\right)\in \Omega .$$

From this, we get the thermodynamic heat $Q:\Omega \subset \mathrm{se}\left(2\right)\to \Omega^{*}\subset \mathrm{se}{\left(2\right)}^{*}$ as

$$Q\left(\lambda ,u\right)=D\Phi \left(\lambda ,u\right)=\left(\frac{1}{\lambda }-\frac{{\left|u\right|}^{2}}{2\lambda^{2}},\frac{1}{\lambda }u\right)$$

and we note that $\Omega^{*}=\left\{\left(\mu ,m\right)\in \mathrm{se}{\left(2\right)}^{*}|\mu +\frac{{\left|m\right|}^{2}}{2}<0\right\}$. The entropy $s:\Omega^{*}\to R$ is obtained as the Legendre transform of Φ:Ω→R as

(71) $$s\left(\mu ,m\right)=1+\log\left(2\pi \right)+\log\left(-\mu -\frac{{\left|m\right|}^{2}}{2}\right).$$

We note the relation

$$\frac{\delta s}{\delta m}=\frac{\delta s}{\delta \mu }m,\;\frac{\delta s}{\delta \mu }={\left(\mu +\frac{{\left|m\right|}^{2}}{2}\right)}^{-1},$$

between the partial derivatives of *s*. From Proposition 2, the Fisher metric is found as

$$\begin{array}{cc} I\left(\beta \right)\left(\delta \beta_{1},\delta \beta_{2}\right) & =-D^{2}\Phi \left(\beta \right)\left(\delta \beta_{1},\delta \beta_{2}\right)\\ \; & =\frac{1}{\lambda^{2}}\left(1-\frac{{\left|u\right|}^{2}}{\lambda }\right)\delta \lambda_{1}\delta \lambda_{2}+\frac{1}{\lambda^{2}}\left(u\cdot \delta u_{1}\delta \lambda_{2}+u\cdot \delta u_{2}\delta \lambda_{1}\right)-\frac{1}{\lambda }\delta u_{1}\cdot \delta u_{2}, \end{array}$$

for every β=(λ,u)∈Ω⊂se(2), i.e.,

$$I\left(\beta \right)=\frac{1}{\lambda^{2}}\left[\begin{array}{cc} 1-\frac{{\left|u\right|}^{2}}{\lambda } & u^{T}\\ u & \lambda I_{2} \end{array}\right].$$

**Affine Lie-Poisson equations and Casimir dissipation.** The affine coadjoint action associated with Equation (70) is found as

$${\mathrm{Ad}}_{{\left(\varphi ,a\right)}^{-1}}^{*}\left(\mu ,m\right)+\theta \left(\varphi ,a\right)=\left(\mu -R_{\varphi }m\cdot Ja-\frac{1}{2}{\left|a\right|}^{2},R_{\varphi }m+Ja\right),$$

from which we directly observe that the entropy Equation (71) is constant on affine coadjoint orbits $O^{\Theta }\subset \mathrm{se}{\left(2\right)}^{*}$ and hence is a Casimir of the Lie-Poisson bracket with cocycle on $\mathrm{se}{\left(2\right)}^{*}$.

Using the expression Θ((λ,u),(γ,v))=−ω(u,v) of the associated two cocycle, we get the Lie-Poisson bracket with cocycle

$$\begin{array}{cc} {\left\{f,g\right\}}_{\Theta }\left(\mu ,m\right) & =\langle \left(\mu ,m\right),\left[\left(\frac{\delta f}{\delta \mu },\frac{\delta f}{\delta m}\right),\left(\frac{\delta g}{\delta \mu },\frac{\delta g}{\delta m}\right)\right]\rangle -\Theta \left(\left(\frac{\delta f}{\delta \mu },\frac{\delta f}{\delta m}\right),\left(\frac{\delta g}{\delta \mu },\frac{\delta g}{\delta m}\right)\right)\\ \; & =\frac{\delta g}{\delta \mu }m\cdot J\frac{\delta f}{\delta m}-\frac{\delta f}{\delta \mu }m\cdot J\frac{\delta g}{\delta m}+\frac{\delta f}{\delta m}\cdot J\frac{\delta g}{\delta m}\\ \; & =\frac{\delta g}{\delta \mu }\omega \left(m,\frac{\delta f}{\delta m}\right)-\frac{\delta f}{\delta \mu }\omega \left(m,\frac{\delta g}{\delta m}\right)+\omega \left(\frac{\delta f}{\delta m},\frac{\delta g}{\delta m}\right). \end{array}$$

Given a Hamiltonian $h:\mathrm{se}{\left(2\right)}^{*}\to R$, one gets the Lie-Poisson equations with cocycle as the following system of ODEs

(72) $$\dot{f}={\left\{f,h\right\}}_{\Theta }\;⟺\;\left\{\begin{array}{c} \;\frac{d}{dt}\mu +m\cdot J\frac{\delta h}{\delta m}=0\\ \frac{d}{dt}m+\frac{\delta h}{\delta \mu }Jm=J\frac{\delta h}{\delta m}. \end{array}\right.$$

These equations determine Hamiltonian dynamics on affine coadjoint orbits that are the level sets of the entropy. From the point of view of thermodynamics, motion remaining on these surfaces is non-dissipative, whereas motion transversal to these surfaces is dissipative. We apply below the geometric approach to include dissipation and hence, motion through affine coadjoint orbits, as considered in general in Section 2.3.3.

Given the entropy Equation (71) and a function $k:\mathrm{se}{\left(2\right)}^{*}\to R$ which commutes with the Hamiltonian, i.e.,

$$\frac{\delta k}{\delta \mu }J\frac{\delta h}{\delta m}=\frac{\delta h}{\delta \mu }J\frac{\delta k}{\delta m}\;⟺\;\frac{\delta k}{\delta \mu }\frac{\delta h}{\delta m}=\frac{\delta h}{\delta \mu }\frac{\delta k}{\delta m}$$

(for instance k=h), the Casimir dissipative/production Equation (40) gives here

$$\begin{array}{cc} \frac{d}{dt}f & ={\left\{f,h\right\}}_{\Theta }-\Lambda \left(\frac{\delta k}{\delta \mu }J\frac{\delta f}{\delta m}-\frac{\delta f}{\delta \mu }J\frac{\delta k}{\delta m}\right)\cdot \left(\frac{\delta k}{\delta \mu }J\frac{\delta s}{\delta m}-\frac{\delta s}{\delta \mu }J\frac{\delta k}{\delta m}\right)\\ \; & ={\left\{f,h\right\}}_{\Theta }-\Lambda \left(\frac{\delta k}{\delta \mu }\frac{\delta f}{\delta m}-\frac{\delta f}{\delta \mu }\frac{\delta k}{\delta m}\right)\cdot \left(\frac{\delta k}{\delta \mu }\frac{\delta s}{\delta m}-\frac{\delta s}{\delta \mu }\frac{\delta k}{\delta m}\right) \end{array}$$

for every *f*, therefore, the following equations emerge

(73) $$\left\{\begin{array}{c} \frac{d}{dt}\mu +m\cdot J\frac{\delta h}{\delta m}=\Lambda \frac{\delta s}{\delta \mu }\left(\frac{\delta k}{\delta \mu }m-\frac{\delta k}{\delta m}\right)\cdot \frac{\delta k}{\delta m}\\ \frac{d}{dt}m+\frac{\delta h}{\delta \mu }Jm=J\frac{\delta h}{\delta m}-\Lambda \frac{\delta s}{\delta \mu }\left(\frac{\delta k}{\delta \mu }m-\frac{\delta k}{\delta m}\right)\frac{\delta k}{\delta \mu }, \end{array}\right.$$

which have the property of preserving the Hamiltonian while dissipating/producing entropy as

$$\begin{array}{cc} \frac{d}{dt}s & =-\Lambda |\frac{\delta k}{\delta \mu }\frac{\delta s}{\delta m}-\frac{\delta s}{\delta \mu }\frac{\delta k}{\delta m}{|}^{2}=-\Lambda {\frac{\delta s}{\delta \mu }}^{2}|\frac{\delta k}{\delta \mu }m-\frac{\delta k}{\delta m}{|}^{2}\\ \; & =-\frac{\Lambda }{{\left(\mu +\frac{{\left|m\right|}^{2}}{2}\right)}^{2}}{\left|\frac{\delta k}{\delta \mu }m-\frac{\delta k}{\delta m}\right|}^{2}. \end{array}$$

They are the SE(2) version of the Equation (67) proposed in the quantum context.

## 4. Variational Principles and (Multi)Symplectic Integrators

In this section, we make use of the geometric setting presented above to propose geometric integrators for some of the equations described earlier. Geometric integrators are numerical schemes designed with the aim to preserve as much possible the geometric structures underlying the equations they discretize [58]. It turns out that the preservation of geometric structures not only produces an improved qualitative behaviour, but also allows for a more accurate long-time integration. One efficient way to derive geometric integrators is to exploit the variational formulation of the continuous equations and to mimic this formulation at the spatial and/or temporal discrete level. For instance, for the ODEs of classical mechanics, a time discretization of the Lagrangian variational formulation permits the derivation of numerical schemes, called *variational integrators*, that are symplectic, exhibit good energy behavior, and inherit a discrete version of Noether’s theorem which guarantees the exact preservation of momenta arising from symmetries, see [59]. These methods are especially well-suited for systems on Lie group [60].

Variational integrators were extended to PDEs in various ways, one way being given by multisymplectic variational integrators ([61,62,63,64]) in which the starting point is a spacetime discretization of the Hamilton principle. Here also, a discrete version of Noether’s theorem for field theories is available in presence of symmetries. We refer to [64,65,66] for recent applications of multisymplectic variational discretizations.

In this section, we will present a geometric discretization of the Lie-Poisson equations with cocycle, see Section 2.3, that is symplectic and preserves the affine coadjoint orbits. We will then extend this approach to treat the case of the polysymplectic version of these Lie-Poisson equations with cocycle, see Section 2.4, by constructing a multisymplectic integrator. To achieve these goals, we will first present the variational principles attached to these equations, by looking at them from the Lagrangian side. Then these variational principles will be discretized in time or in space and time.

### 4.1. Preliminaries on Variational Lie Group Integrators

We very briefly recall the broad idea of variational integrators and refer to [59] for the detailed description. They are based on a discrete version of the Hamilton principle given, for a Lagrangian L:TQ→R, as

(74) $$\delta \int_{0}^{T}L\left(q\left(t\right),\dot{q}\left(t\right)\right)dt=0,$$

for arbitrary variations of the curve q(t) with fixed extremities at t=0,T.

**Euler-Poincaré and Lie-Poisson equations.** We will be especially interested in the case where the configuration manifold is a Lie group, Q=G, and the Lagrangian L:TG→R is right *G*-invariant. In this case, *L* induces a reduced Lagrangian *ℓ* on the quotient space (TG)/G identified with the Lie algebra g, i.e., we get ℓ:g→R defined by the relation $L\left(g,\dot{g}\right)=\ell \left(\dot{g}g^{-1}\right)$. The Euler-Lagrange equations for *L* are equivalent to equations on g written in terms of the reduced Lagrangian ℓ:g→R, called the *Euler-Poincaré equations*. They are obtained by computing the variational principle for *ℓ* induced by the Hamilton principle Equation (74). It is given by

(75) $$\delta \int_{0}^{T}\ell \left(\xi \left(t\right)\right)dt=0,\;\mathrm{for}\;\delta \xi =\partial_{t}\eta +\left[\eta ,\xi \right]$$

and yields the Euler-Poincaré equations

(76) $$\frac{d}{dt}\frac{\delta \ell }{\delta \xi }+{\mathrm{ad}}_{\xi }^{*}\frac{\delta \ell }{\delta \xi }=0$$

for the curve ξ(t)∈g. In Equation (75), η(t) is an arbitrary curve in g vanishing at the extremities. If the Lagrangian is hyperregular, one can rewrite the Euler-Lagrange equations and the Euler-Poincaré equations in terms of the Hamiltonian associated with *L* or *ℓ*. In terms of the Hamiltonian $h:g^{*}\to R$ obtained by the Legendre transform of *ℓ*, Equation (76) become the *Lie-Poisson equations*

(77) $$\frac{d}{dt}\mu +{\mathrm{ad}}_{\frac{\delta h}{\delta \mu }}^{*}\mu =0,$$

see [31].

**Variational integrators.** Let *Q* be a configuration manifold and let L:TQ→R be a Lagrangian. Suppose that a time step Δt was fixed, denote by $\left\{t_{k}=k\Delta t|k=0,\ldots ,N\right\}$ the sequence of time, and by $q_{d}:{\left\{t_{k}\right\}}_{k=0}^{N}\to Q$, $q_{d}\left(t_{k}\right)=q_{k}$ a discrete curve. A *discrete Lagrangian* is a map $L_{d}:Q\times Q\to R$, $L_{d}=L_{d}\left(q_{k},q_{k+1}\right)$ that approximates the action integral of *L* along the curve segment between $q_{k}$ and $q_{k+1}$, that is, we have

$$L_{d}\left(q_{k},q_{k+1}\right)\approx \int_{t_{k}}^{t_{k+1}}L\left(q\left(t\right),\dot{q}\left(t\right)\right)dt,$$

where $q\left(t_{k}\right)=q_{k}$ and $q\left(t_{k+1}\right)=q_{k+1}$. Usually this approximation is related to some numerical quadrature rule of the integral above. The discrete analogue of Hamilton’s principle Equation (74) reads

(78) $$\delta \sum_{k=0}^{N-1}L_{d}\left(q_{k},q_{k+1}\right)=0$$

for all variations $\delta q_{d}$ of $q_{d}$ with vanishing endpoints. After taking variations and applying a discrete integration by parts formula (change of indices), we obtain the *discrete Euler-Lagrange* equations:

(79) $$D_{2}L_{d}\left(q_{k-1},q_{k}\right)+D_{1}L_{d}\left(q_{k},q_{k+1}\right)=0,\;\forall \;k\in \left\{1,\cdots ,N-1\right\}.$$

These equations define, under appropriate conditions, an algorithm which solves for $q_{k+1}$ knowing the two previous configuration variables $q_{k}$ and $q_{k-1}$.

To define the discrete momentum maps, one first needs to consider the discrete Legendre transforms defined by

(80) $$\begin{array}{cc} F^{+}L_{d}\left(q_{k},q_{k+1}\right) & :=D_{2}L_{d}\left(q_{k},q_{k+1}\right)\in T_{q_{k+1}}^{*}Q\\ F^{-}L_{d}\left(q_{k},q_{k+1}\right) & :=-D_{1}L_{d}\left(q_{k},q_{k+1}\right)\in T_{q_{k}}^{*}Q. \end{array}$$

Then, given a Lie group action Φ:G×Q→Q, the *discrete Lagrangian momentum maps* $J_{L_{d}}^{+},J_{L_{d}}^{-}:Q\times Q\to g^{*}$ are defined by

(81) $$\begin{array}{cc} \langle J_{L_{d}}^{+}\left(q_{k},q_{k+1}\right),\xi \rangle & =\langle D_{2}L_{d}\left(q_{k},q_{k+1}\right),\xi_{Q}\left(q_{k+1}\right)\rangle \\ \langle J_{L_{d}}^{-}\left(q_{k},q_{k+1}\right),\xi \rangle & =\langle -D_{1}L_{d}\left(q_{k},q_{k+1}\right),\xi_{Q}\left(q_{k}\right)\rangle . \end{array}$$

If the discrete curve ${\left\{q^{j}\right\}}_{j=0}^{N}$ satisfies the discrete Euler-Lagrange equations then we have the equality

(82) $$J_{L_{d}}^{+}\left(q_{k-1},q_{k}\right)=J_{L_{d}}^{-}\left(q_{k},q_{k+1}\right),\;\mathrm{for}\;\mathrm{all}\;j=1,\ldots ,N-1.$$

If the discrete Lagrangian $L_{d}$ is *G*-invariant under the diagonal action of *G* induced by Φ on Q×Q, then the two discrete momentum maps coincide, $J_{L_{d}}^{-}=J_{L_{d}}^{+}=:J_{L_{d}}$, therefore from Equation (82), we obtain that $J_{L_{d}}$ is a conserved quantity along the discrete curve solution of Equation (79), that is,

(83) $$J_{L_{d}}\left(q_{k},q_{k+1}\right)=J_{L_{d}}\left(q_{k-1},q_{k}\right),\;\mathrm{for}\;\mathrm{all}\;j=1,\ldots ,N-1.$$

This result is referred to as the *discrete Noether’s theorem*.

The symplectic character of the integrator is obtained by showing that the scheme $\left(q_{k-1},q_{k}\right)\mapsto \left(q_{k},q_{k+1}\right)$ preserves the discrete symplectic two-forms $\Omega_{L_{d}}^{\pm }:={\left(F^{\pm }L_{d}\right)}^{*}\Omega_{\mathrm{can}}$ on Q×Q, where $\Omega_{\mathrm{can}}$ is the canonical symplectic two-form on $T^{*}Q$, see [59].

**Discrete Euler-Poincaré equations.** For Lie groups, variational discretization and the associated discrete Lagrangian reductions, was started in [60,67], and referred to as *Lie group variational integrators*. The essential idea behind such integrators is to discretize Hamilton’s principle and to update group elements using group operations. For the case of invariant systems on Lie group, one chooses a discrete Lagrangian that inherits the invariance of the continuous Lagrangian, i.e., $L_{d}:G\times G\to R$ satisfies $L_{d}\left(g_{k}h,g_{k+1}h\right)=L_{d}\left(g_{k},g_{k+1}\right)$, for all h∈G.

From this invariance, one defines the reduced discrete Lagrangian $L_{d}$ on the associated quotient space (G×G)/G identified with *G* with quotient map $\left(g_{k},g_{k+1}\right)\in G\times G\mapsto g_{k+1}g_{k}^{-1}\in G$, i.e., the two discrete Lagrangians are related as $L_{d}\left(g_{k},g_{k+1}\right)=L_{d}\left(g_{k+1}g_{k}^{-1}\right)$, this is the point of view developed in [60]. The discrete Hamilton principle Equation (78) for $L_{d}$ induces a discrete Euler-Poincaré variational principle for $L_{d}$ that yields the discrete Euler-Poincaré equations on *G*. Numerically speaking it is desirable to obtain the algorithm on a vector space rather than on a Lie group. For this aim, a local diffeomorphism τ:g→G with τ(0)=e is introduced to express small discrete changes in the group configuration through unique Lie algebra elements. Such a map is referred to as a *retraction map* ([68,69]). The discrete reduced Lagrangian is transported into a discrete Lagrangian $\ell_{d}$ defined on a neighborhood of 0 in g via the relation

(84) $$\ell_{g}\left(\xi_{k}\right)=L_{d}\left(g_{k+1}g_{k}^{-1}\right),\;\mathrm{with}\;\tau \left(\Delta t\xi_{k}\right)=g_{k+1}g_{k}^{-1}.$$

The relation on the right in Equation (84) is thought of as a discrete version of $\xi =\dot{g}g^{-1}$.

The discrete Euler-Poincaré equations for $\ell_{d}$ are obtained by computing the discrete variational principle induced on the discrete action $\sum_{k=0}^{N-1}\ell_{d}\left(\xi_{k}\right)$ from the discrete Hamilton principle $\delta \sum_{k=0}^{N-1}L\left(g_{k},g_{k+1}\right)=0$ recalled above in Equation (78). The main step in this process is to compute the variations $\delta \xi_{k}$ of $\xi_{k}=\frac{1}{\Delta t}\tau^{-1}\left(g_{k+1}g_{k}^{-1}\right)$ induced by arbitrary variations $\delta g_{k}$. One finds the expression

(85) $$\delta \xi_{k}=\frac{1}{\Delta t}d^{L}\tau^{-1}\left(\Delta t\xi_{k}\right)\cdot \left({\mathrm{Ad}}_{\tau {\left(\Delta t\xi_{k}\right)}^{-1}}\eta_{k+1}-\eta_{k}\right),$$

where $\eta_{k}=\delta g_{k}g_{k}^{-1}$ and $d^{L}\tau^{-1}\left(\xi \right):g\to g$ is the inverse to the left trivialized derivative of τ, $d^{L}\tau \left(\xi \right):g\to g$ defined by

(86) $$d^{L}\tau \left(\xi \right)\cdot \eta =\tau {\left(\xi \right)}^{-1}D\tau \left(\xi \right)\cdot \eta .$$

The *discrete Euler-Poincaré variational principle* thus reads

(87) $$\delta \sum_{k=0}^{N-1}\ell_{d}\left(\xi_{k}\right)=0,$$

with respect to variations $\delta \xi_{k}$ of the form Equation (85) with $\eta_{k}$ vanishing at the endpoints. It yields the *discrete Euler-Poincaré equations*.

(88) $${\mathrm{Ad}}_{\tau {\left(\Delta t\xi_{k-1}\right)}^{-1}}^{*}\mu_{k-1}-\mu_{k}=0,\;\mu_{k}:=d^{L}\tau^{-1}{\left(\Delta t\xi_{k}\right)}^{*}\frac{\delta \ell }{\delta \xi_{k}}.$$

Here $d^{L}\tau^{-1}{\left(\xi \right)}^{*}:g^{*}\to g^{*}$ denotes the dual map to $d^{L}\tau^{-1}\left(\xi \right):g\to g$. We refer to [60,67,69,70] for the discrete Euler-Poincaré equations.

Being equivalent to the discrete Euler-Lagrange equations on the Lie group, this scheme is equivalent to a symplectic scheme $\left(g_{k-1},g_{k}\right)\mapsto \left(g_{k},g_{k+1}\right)$ on G×G. From the discrete Noether theorem, the scheme also preserves the discrete momentum map and the coadjoint orbits $O\subset g^{*}$. Moreover, the scheme $\mu_{k-1}\in O\mapsto \mu_{k}\in O$ is symplectic on coadjoint orbits with respect to the Kirillov-Kostant-Souriau symplectic form, see [60]. Please note that the discrete momentum map is computed as

$$J_{L_{d}}\left(g_{k},g_{k+1}\right)=\frac{1}{\Delta t}{\mathrm{Ad}}_{g_{k}}^{*}\left(d^{L}\tau^{-1}{\left(\Delta t\xi_{k}\right)}^{*}\frac{\delta \ell }{\delta \xi_{k}}\right)=\frac{1}{\Delta t}{\mathrm{Ad}}_{g_{k}}^{*}\mu_{k},$$

which is readily seen to be preserved, $J_{L_{d}}\left(g_{k-1},g_{k}\right)=J_{L_{d}}\left(g_{k},g_{k+1}\right)$, along the solutions of Equation (88)

### 4.2. Central Extensions and Variational Principle for the Lie-Poisson Equations with Cocycle

We considered in Equation (36) the Lie-Poisson equations with cocycle given by

(89) $$\frac{d}{dt}\mu +{\mathrm{ad}}_{\frac{\delta h}{\delta \mu }}^{*}\mu =\Theta \left(\frac{\delta h}{\delta \mu },\cdot \right),$$

associated with the Souriau symplectic model. Our aim is to derive a geometric integrator for this system that is symplectic and preserves the affine coadjoint orbits for general Hamiltonian. One systematic step is to look at Equation (89) from the Lagrangian side, as it was done for the ordinary Lie-Poisson equations above. Assuming that *h* is hyperregular, we can take the associated Lagrangian ℓ:g→R and rewrite the equations as

(90) $$\frac{d}{dt}\frac{\delta \ell }{\delta \xi }+{\mathrm{ad}}_{\xi }^{*}\frac{\delta \ell }{\delta \xi }=\Theta \left(\xi ,\cdot \right),$$

for a curve ξ(t)∈g. However, in general (i.e., for arbitrary *ℓ*, arbitrary g, and arbitrary Θ) there is no natural variational principle for these equations, in the sense of a variational principle induced from the ordinary Hamilton principle for a Lagrangian L:TG→R.

Nevertheless, there is a way to interpret the system Equation (90) as being induced by an ordinary Euler-Poincaré equations on a central extension of the Lie group *G*, integrating the Lie algebra cocycle Θ. This is related to a well-known fact that affine coadjoint orbits can be seen as ordinary coadjoint orbits of a central extension. We recall this fact below.

**Lie group operations on central extensions.** We shall focus on topologically trivial central extensions of finite dimensional Lie groups by R. The central extended group is thus of the form $\hat{G}=G\times R$ with group multiplication

(g,α)(h,β)=(gh,α+β+B(g,h))

where B:G×G→R is a group two-cocycle, i.e., it satisfies

B(f,g)+B(fg,h)=B(f,gh)+B(g,h)

for all f,g,h∈G. It can always been chosen such that B(e,g)=B(g,e)=0, in which case we have $B\left(g,g^{-1}\right)=B\left(g^{-1},g\right)$ and ${\left(g,\alpha \right)}^{-1}=\left(g^{-1},-\alpha -B\left(g^{-1},g\right)\right)$. One obtains from this the expression of the adjoint and coadjoint actions as

(91) $$\begin{array}{cc} {\mathrm{Ad}}_{\left(g,\alpha \right)}\left(\eta ,v\right) & =({\mathrm{Ad}}_{g}\eta ,v+\langle \theta (g^{-1}),\eta \rangle ) \end{array}$$

(92) $$\begin{array}{cc} {\mathrm{Ad}}_{\left(g,\alpha \right)}^{*}\left(\mu ,a\right) & =({\mathrm{Ad}}_{g}^{*}\mu +a\theta \left(g^{-1}\right),a) \end{array}$$

where the group one-cocycle $\theta \in C^{\infty }\left(G,g^{*}\right)$ is defined by

(93) $$\langle \theta (g),\eta \rangle =D_{2}B\left(g^{-1},g\right)\cdot \eta g-D_{1}B\left(g,g^{-1}\right)\cdot \eta g.$$

Equation (92) shows that the ordinary coadjoint orbits of $\hat{G}$ through (μ,1) are affine coadjoint orbits of *G*. We have the corresponding formulas

(94) $$\begin{array}{cc} {\mathrm{ad}}_{\left(\xi ,u\right)}\left(\eta ,v\right) & =\left([\xi ,\eta ],-\Theta (\xi ,\eta )\right) \end{array}$$

(95) $$\begin{array}{cc} {\mathrm{ad}}_{\left(\xi ,u\right)}^{*}\left(\mu ,a\right) & =({\mathrm{ad}}_{\xi }^{*}\mu -a\Theta \left(\xi ,\cdot \right),0). \end{array}$$

**Euler-Poincaré and Lie-Poisson equations on central extensions.** From Equation (95), the Euler-Poincaré equations for a reduced Lagrangian $\hat{\ell }:\hat{g}=g\times R\to R$ take the form

(96) $$\left\{\begin{array}{c} \frac{d}{dt}\frac{\delta \hat{\ell }}{\delta \xi }+{\mathrm{ad}}_{\xi }^{*}\frac{\delta \hat{\ell }}{\delta \xi }=\frac{\delta \hat{\ell }}{\delta u}\Theta \left(\xi ,\cdot \right)\\ \frac{d}{dt}\frac{\delta \hat{\ell }}{\delta u}=0. \end{array}\right.$$

They are the critical conditions for the Euler-Poincaré variational principle

(97) $$\delta \int_{0}^{T}\hat{\ell }\left(\xi \left(t\right),u\left(t\right)\right)dt=0,\;\mathrm{for}\;\delta \xi =\partial_{t}\eta +\left[\eta ,\xi \right],\;\delta u=\partial_{t}v-\Theta \left(\eta ,\xi \right),$$

which is just a special instance of Equation (75) applied to central extensions. In Equation (97) η(t)∈g and v(t)∈R are arbitrary curves vanishing at the extremities.

Given a Lagrangian ℓ:g→R, one can then define the Lagrangian

(98) $$\hat{\ell }\left(\xi ,u\right)=\ell \left(\xi \right)+\frac{1}{2}u^{2}$$

on $\hat{g}$ for which Equation (96) does reduce to Equation (90) if the initial condition for the curve u(t) is u(0)=1. This means that Equation (90) have a natural Euler-Poincaré variational formulation, if one interprets them as an invariant subsystem of an Euler-Poincaré equation on a central extension of *G* via a group two-cocycle *B* that integrates the one-cocycle θ as in Equation (93).

The same reasoning also directly applies on the Hamiltonian side, in which case the Lie-Poisson equation with cocycle Equation (89) is an invariant subsystem of an ordinary Lie-Poisson equation associated with a central extension of *G*.

All these considerations are standard, see, e.g., [29,31].

### 4.3. Variational Symplectic Integrators for the Lie-Poisson Equations with Cocycle

Here we shall present a geometric symplectic Lie group integrator for Lie-Poisson equations with cocycle Equation (36) that preserves the affine coadjoint orbits for general Hamiltonian. In particular, the scheme preserves the affine Kirillov-Kostant-Souriau symplectic form on these affine coadjoint orbits. We shall use the Euler-Poincaré variational formulation on central extensions presented in Section 4.2.

**Some useful identities.** Given a central extension $\hat{G}=G\times R$, we shall consider the retraction map $\tau :\hat{g}\to \hat{G}$ defined by

(99) $$\tau \left(\xi ,u\right)=(\bar{\tau }\left(\xi \right),u)$$

where $\bar{\tau }:g\to G$ is a retraction map for *G*. To derive the discrete Euler-Poincaré equations we shall need several identities involving $d^{L}\tau$ and $d^{L}\bar{\tau }$, see Equation (86), that are shown in the next Lemma.

**Lemma** **14.** *For a local diffeomorphism of the form Equation (99) on central extension, we have the following identities*

(a) $d^{L}\tau \left(\xi ,u\right)\cdot \left(\eta ,v\right)=\left(d^{L}\bar{\tau }\left(\xi \right)\cdot \eta ,v-D_{2}B\left(\bar{\tau }\left(\xi \right),e\right)\cdot \left(d^{L}\bar{\tau }\left(\xi \right)\cdot \eta \right)\right)$
(b) $d^{L}\tau {\left(\xi ,u\right)}^{*}\cdot \left(\mu ,a\right)=\left(d^{L}\bar{\tau }{\left(\xi \right)}^{*}\left(\mu -aD_{2}B\left(\bar{\tau }\left(\xi \right),e\right)\right),a\right)$
(c) $d^{L}\tau^{-1}\left(\xi ,u\right)\cdot \left(\zeta ,w\right)=\left(d^{L}{\bar{\tau }}^{-1}\left(\xi \right)\cdot \zeta ,w+D_{2}B\left(\bar{\tau }\left(\xi \right),e\right)\cdot \zeta \right)$
(d) $d^{L}\tau^{-1}{\left(\xi ,u\right)}^{*}\cdot \left(\mu ,a\right)=\left(d^{L}{\bar{\tau }}^{-1}{\left(\xi \right)}^{*}\cdot \mu +aD_{2}B\left(\bar{\tau }\left(\xi \right),e\right),a\right)$ *,*

*where* B:G×G→R *is the group two-cocycle.*

**Proof.**  These identities are proven as follows.

(a) Using the definition of $d^{L}\tau$, we compute
  $$\begin{array}{cc} d^{L}\tau \left(\xi ,u\right)\cdot \left(\eta ,v\right) & =\tau {\left(\xi ,u\right)}^{-1}\left(D\tau \left(\xi ,u\right)\cdot \left(\eta ,v\right)\right)\\ \; & ={\left(\bar{\tau }\left(\xi \right),u\right)}^{-1}\left(\bar{\tau }\left(\xi \right),D\bar{\tau }\left(\xi \right)\cdot \eta ,u,v\right)\\ \; & =(\bar{\tau }{\left(\xi \right)}^{-1}D\bar{\tau }\left(\xi \right)\cdot \eta ,v+D_{2}B\left(\bar{\tau }{\left(\xi \right)}^{-1},\bar{\tau }\left(\xi \right)\right)\cdot \left(D\bar{\tau }\left(\xi \right)\cdot \eta \right))\\ \; & =(d^{L}\bar{\tau }\left(\xi \right)\cdot \eta ,v+D_{2}B\left(\bar{\tau }{\left(\xi \right)}^{-1},\bar{\tau }\left(\xi \right)\right)\cdot \left(\bar{\tau }\left(\xi \right)d^{L}\bar{\tau }\left(\xi \right)\cdot \eta \right)) \end{array}$$
  where in the third equality, we used the formula for the tangent lift of left translation on $\hat{G}$. Using the properties of the group two-cocycle *B*, we get the identity
  $$D_{2}B\left(g^{-1},g\right)\cdot \left(g\eta \right)=-D_{2}B\left(g,e\right)\cdot \eta ,$$
  for all g∈G and η∈g. Hence we get the result.
(b) Taking the dual map and using (a), we get
  $$\begin{array}{cc} \; & \langle d^{L}\tau {\left(\xi ,u\right)}^{*}\cdot \left(\mu ,a\right),\left(\eta ,v\right)\rangle \\ \; & =\langle \left(\mu ,a\right),d^{L}\tau \left(\xi ,u\right)\cdot \left(\eta ,v\right)\rangle \\ \; & =\langle \mu ,d^{L}\bar{\tau }\left(\xi \right)\cdot \eta \rangle +a\left(v-D_{2}B\left(\bar{\tau }\left(\xi \right),e\right)\cdot \left(d^{L}\bar{\tau }\left(\xi \right)\cdot \eta \right)\right)\\ \; & =\langle d^{L}\bar{\tau }{\left(\xi \right)}^{*}\cdot \mu -a\;d^{L}\bar{\tau }{\left(\xi \right)}^{*}D_{2}B\left(\bar{\tau }\left(\xi \right),e\right),\eta \rangle +av, \end{array}$$
  which proves the result.
(c) It follows by (a) and by inverting the relation $\left(\zeta ,w\right)=d^{L}\tau \left(\xi ,u\right)\cdot \left(\eta ,v\right)$
  $$\left(\zeta ,w\right)=d^{L}\tau \left(\xi ,u\right)\cdot \left(\eta ,v\right)=\left(d^{L}\bar{\tau }\left(\xi \right)\cdot \eta ,v+D_{2}B\left(\bar{\tau }{\left(\xi \right)}^{-1},\bar{\tau }\left(\xi \right)\right)\cdot \left(\bar{\tau }\left(\xi \right)d^{L}\bar{\tau }\left(\xi \right)\cdot \eta \right)\right)$$
  is equivalent to
  $$\left(\eta ,v\right)=\left(d^{L}{\bar{\tau }}^{-1}\left(\xi \right)\cdot \zeta ,w-D_{2}B\left(\bar{\tau }{\left(\xi \right)}^{-1},\bar{\tau }\left(\xi \right)\right)\cdot \left(\bar{\tau }\left(\xi \right)\zeta \right)\right)$$
(d) This follows by taking the dual map and using (c) as earlier. □

 ☐

**Variational discretization of the Lie-Poisson equations with cocycle.** With the previous result, we first give below a symplectic integrator for the Euler-Poincaré equations on central extensions. Then we will show how this provides a symplectic integrator for the Lie-Poisson equations with cocycle.

**Proposition** **15** (Discrete Euler-Poincaré equations on central extensions). *The following are equivalent:*

(a) *The discrete curve*$\left(\xi_{k},u_{k}\right)$*is critical for the discrete Euler-Poincaré variational principle*
  $$\delta \sum_{k}\hat{\ell }\left(\xi_{k},u_{k}\right)=0,$$
  *with respect to variations*
  $$\left\{\begin{array}{c} \delta \xi_{k}=\frac{1}{\Delta t}d^{L}{\bar{\tau }}^{-1}\left(\Delta t\xi_{k}\right)\cdot \left({\mathrm{Ad}}_{\tau {\left(\Delta t\xi_{k}\right)}^{-1}}\eta_{k+1}-\eta_{k}\right)\\ \delta u_{k}=\frac{1}{\Delta t}\left(v_{k+1}-v_{k}-D_{2}B\left(\bar{\tau }\left(\Delta t\xi_{k}\right),e\right)\cdot \eta_{k}+D_{1}B\left(e,\bar{\tau }\left(\Delta t\xi_{k}\right)\right)\cdot \eta_{k+1}\right) \end{array}\right.$$
  *where*$\eta_{k}\in g$ and $v_{k}\in R$ *are arbitrary discrete curves vanishing at the endpoints.*
(b) *The discrete curve* $\left(\xi_{k},u_{k}\right)$ *is a solution of the discrete Euler-Poincaré equations*
  (100) $$\left\{\begin{array}{c} {\mathrm{Ad}}_{\tau {\left(\Delta t\xi_{k-1}\right)}^{-1}}^{*}\mu_{k-1}+a_{k-1}\theta \left(\bar{\tau }\left(\Delta t\xi_{k-1}\right)\right)-\mu_{k}=0\\ a_{k-1}-a_{k}=0 \end{array}\right.$$
  *with*
  (101) $$\left\{\begin{array}{c} \mu_{k}=d^{L}{\bar{\tau }}^{-1}{\left(\Delta t\xi_{k}\right)}^{*}\frac{\delta \hat{\ell }}{\delta \xi_{k}}+\frac{\delta \hat{\ell }}{\delta u_{k}}D_{2}B\left(\bar{\tau }\left(\Delta t\xi_{k}\right),e\right)\\ a_{k}=\frac{\delta \hat{\ell }}{\delta u_{k}}. \end{array}\right.$$

**Proof.**  We use the discrete Euler-Poincaré formulation Equations (87)–(118). For (a), we use Equation (85) and Lemma 14, and we compute

$$\begin{array}{cc} \; & \delta (\xi_{k},u_{k})\\ \; & =\frac{1}{\Delta t}d^{L}\tau^{-1}\left(\Delta t\xi_{k},\Delta tu_{k}\right)\cdot \left({\mathrm{Ad}}_{\tau {\left(\Delta t\xi_{k},\Delta tu_{k}\right)}^{-1}}\left(\eta_{k+1},v_{k+1}\right)-\left(\eta_{k},v_{k}\right)\right)\\ \; & =\frac{1}{\Delta t}d^{L}\tau^{-1}\left(\Delta t\xi_{k},\Delta tu_{k}\right)\cdot \left({\mathrm{Ad}}_{\bar{\tau }{\left(\Delta t\xi_{k}\right)}^{-1}}\eta_{k+1}-\eta_{k},v_{k+1}-v_{k}+\langle \theta \left(\bar{\tau }\left(\Delta t\xi_{k}\right)\right),\eta_{k+1}\rangle \right)\\ \; & =\frac{1}{\Delta t}(d^{L}{\bar{\tau }}^{-1}\left(\Delta t\xi_{k}\right)\cdot \left({\mathrm{Ad}}_{\tau {\left(\Delta t\xi_{k}\right)}^{-1}}\eta_{k+1}-\eta_{k}\right),\\ \; & \;\;v_{k+1}-v_{k}+\langle \theta \left(\bar{\tau }\left(\Delta t\xi_{k}\right)\right),\eta_{k+1}\rangle +D_{2}B\left(\bar{\tau }\left(\Delta t\xi_{k}\right),e\right)\cdot \left({\mathrm{Ad}}_{\tau {\left(\Delta t\xi_{k}\right)}^{-1}}\eta_{k+1}-\eta_{k}\right)). \end{array}$$

Using the identity $\langle \theta (g),\xi \rangle +D_{2}B\left(g,e\right)\cdot {\mathrm{Ad}}_{g^{-1}}\xi =D_{1}B\left(e,g\right)\cdot \eta$, we get the desired result.

For (b), we use the formula for the coadjoint action on central extension to get

$$\begin{array}{cc} \; & {\mathrm{Ad}}_{\tau {\left(\Delta t\xi_{k-1},\Delta tu_{k-1}\right)}^{-1}}^{*}\left(\mu_{k-1},a_{k-1}\right)-\left(\mu_{k},a_{k}\right)\\ \; & \;=\left({\mathrm{Ad}}_{\tau {\left(\Delta t\xi_{k-1}\right)}^{-1}}^{*}\mu_{k-1}+a_{k-1}\theta \left(\bar{\tau }\left(\Delta t\xi_{k-1}\right)\right),a_{k-1}\right)-\left(\mu_{k},a_{k}\right) \end{array}$$

which proves Equation (100). Then, to get Equation (101), we note that

$$\begin{array}{cc} (\mu_{k},a_{k}): & =d^{L}\tau^{-1}{\left(\Delta t\xi_{k},\Delta tu_{k}\right)}^{*}\left(\frac{\delta \ell }{\delta \xi_{k}},\frac{\delta \ell }{\delta u_{k}}\right)\\ \; & =\left(d^{L}{\bar{\tau }}^{-1}{\left(\Delta t\xi_{k}\right)}^{*}\frac{\delta \ell }{\delta \xi_{k}}+\frac{\delta \ell }{\delta u_{k}}D_{2}B\left(\bar{\tau }\left(\Delta t\xi_{k}\right),e\right),\frac{\delta \ell }{\delta u_{k}}\right) \end{array}$$

by Lemma 14. ☐

We note that the relation with the solution $\left(g_{k},\alpha_{k}\right)$ of the discrete Euler-Lagrange on the Lie group $\hat{G}$ is given as

$$\left(\xi_{k},u_{k}\right)=\frac{1}{\Delta t}\tau^{-1}\left(\left(g_{k+1},\alpha_{k+1}\right){\left(g_{k},\alpha_{k}\right)}^{-1}\right)$$

which is explicitly given by the relations

(102) $$\begin{array}{cc} \xi_{k} & =\frac{1}{\Delta t}\tau^{-1}\left(g_{k+1}g_{k}^{-1}\right)\\ u_{k} & =\frac{1}{\Delta t}\left(\alpha_{k+1}-\alpha_{k}-B\left(g_{k},g_{k}^{-1}\right)+B\left(g_{k+1},g_{k}^{-1}\right)\right). \end{array}$$

Similarly, the variations $\eta_{k},v_{k}$ used in discrete Euler-Poincaré variational principle are related to the variations $\delta g_{k}$, $\delta \alpha_{k}$ used in the discrete Hamilton principle via the equality $\left(\eta_{k},v_{k}\right)=\left(\delta g_{k},\delta \alpha_{k}\right){\left(g_{k},\alpha_{k}\right)}^{-1}=\left(\delta g_{k}g_{k}^{-1},\delta \alpha_{k}+D_{1}B\left(g_{k},g_{k}^{-1}\right)\cdot \delta g_{k}\right)$.

The discrete momentum map $J_{L_{d}}:\hat{G}\times \hat{G}\to {\hat{g}}^{*}$ is computed as

$$J_{L_{d}}(\left(g_{k},\alpha_{k}\right),\left(g_{k+1},\alpha_{k+1}\right)=\frac{1}{\Delta t}\left({\mathrm{Ad}}_{g_{k}}^{*}\mu_{k}+a_{k}\theta \left(g_{k}^{-1}\right),a_{k}\right)$$

where $\left(\mu_{k},a_{k}\right)$ are given in Equation (101) and relation Equation (102) are assumed. It is readily seen that $J_{L_{d}}$ is preserved along the solutions of Equation (100).

The symplectic integrator for the Lie-Poisson equations with cocycle is deduced as follows.

**Proposition 16.**  (Symplectic integrator for Lie-Poisson equations with cocycle) *Let* $h:g^{*}\to R$ *be a Hamiltonian assumed to be hyperregular, with associated Lagrangian* ℓ:g→R*. Then the numerical scheme*

(103) $$\;{\mathrm{Ad}}_{\tau {\left(\Delta t\xi_{k-1}\right)}^{-1}}^{*}\mu_{k-1}+\theta \left(\bar{\tau }\left(\Delta t\xi_{k-1}\right)\right)-\mu_{k}=0$$

*with*

(104) $$\mu_{k}=d^{L}{\bar{\tau }}^{-1}{\left(\Delta t\xi_{k}\right)}^{*}\frac{\delta \ell }{\delta \xi_{k}}+D_{2}B\left(\bar{\tau }\left(\Delta t\xi_{k}\right),e\right)$$

*is a symplectic scheme for the Lie-Poisson equations with cocycle*

(105) $$\frac{d}{dt}\mu +{\mathrm{ad}}_{\frac{\delta h}{\delta \mu }}^{*}\mu =\Theta \left(\frac{\delta h}{\delta \mu },\cdot \right).$$

*It preserves the affine coadjoint orbits*

$$O=\{{\mathrm{Ad}}_{g^{-1}}^{*}\mu +\theta \left(g\right)|G\in G\}$$

*and* $\mu_{k-1}\mapsto \mu_{k}$ *is symplectic relative to the affine Kirillov-Kostant-Souriau symplectic form*

$$\omega_{O}\left(\mu \right)\left({\mathrm{ad}}_{\xi }^{*}\mu -\Theta \left(\xi ,\cdot \right),{\mathrm{ad}}_{\eta }^{*}\mu -\Theta \left(\eta ,\cdot \right)\right)=\langle \mu ,[\xi ,\eta ]\rangle -\Theta \left(\xi ,\eta \right).$$

**Proof.**  It is a direct consequence of Proposition 15, by choosing the reduced Lagrangian Equation (98), taking the initial condition $a_{0}=1$ and noting that $a_{k+1}=a_{k}=1$. ☐

It is possible to rewrite the scheme in a way that is more advantageous from the point of view of implementation. By inserting Equation (104) in Equation (103) and using the identity

$${\mathrm{Ad}}_{g^{-1}}^{*}D_{2}B\left(g,e\right)+\theta \left(g\right)=D_{1}B\left(e,g\right)$$

we get the scheme in terms of $\xi_{k}$ as

(106) $$\begin{array}{cc} \; & {\mathrm{Ad}}_{\tau {\left(\Delta t\xi_{k-1}\right)}^{-1}}^{*}d^{L}{\bar{\tau }}^{-1}{\left(\Delta t\xi_{k-1}\right)}^{*}\frac{\delta \ell }{\delta \xi_{k-1}}-d^{L}{\bar{\tau }}^{-1}{\left(\Delta t\xi_{k}\right)}^{*}\frac{\delta \ell }{\delta \xi_{k}}\\ \; & \;\;\;\;\;\;+D_{1}B\left(e,\bar{\tau }\left(\Delta t\xi_{k-1}\right)\right)-D_{2}B\left(\bar{\tau }\left(\Delta t\xi_{k}\right),e\right)=0. \end{array}$$

It is also often assumed that the retraction map τ satisfies τ(−ξ)τ(ξ)=e. In this case, we have the identity ${\mathrm{Ad}}_{\tau (\xi )}^{*}d^{L}\tau^{-1}{\left(-\xi \right)}^{*}=d^{L}\tau^{-1}\left(\xi \right)$, see [69], and the scheme Equation (106) takes the form

(107) $$\begin{array}{cc} \; & d^{L}{\bar{\tau }}^{-1}{\left(-\Delta t\xi_{k-1}\right)}^{*}\frac{\delta \ell }{\delta \xi_{k-1}}-d^{L}{\bar{\tau }}^{-1}{\left(\Delta t\xi_{k}\right)}^{*}\frac{\delta \ell }{\delta \xi_{k}}+D_{1}B\left(e,\bar{\tau }\left(\Delta t\xi_{k-1}\right)\right)-D_{2}B\left(\bar{\tau }\left(\Delta t\xi_{k}\right),e\right)=0. \end{array}$$

In absence of the last two terms, we recover the most practically used form of the ordinary discrete Euler-Poincaré equations, e.g., [69]. The last two terms correspond to a discretization of the cocycle which ensures that the resulting scheme is symplectic on each affine coadjoint orbit. It is clear that such a form is not likely to be guessed from the continuous equations without having at hands the discrete variational principle.

**Remark** **17** (Choice of retraction map). For an exposition of retraction maps, such as canonical coordinates of the first and second kind, and their applications to Lie group methods, the reader is referred to [68]. A possible choice is the exponential map exp:g→G. In this case, $d^{L}\exp\left(\xi \right)\cdot \eta$ and $d^{L}\exp^{-1}\left(\xi \right)\cdot \eta$ are given as series which are truncated in order to achieve a desired order of accuracy [58]. A standard choice is the Cayley map cay:g→G defined by $\mathrm{cay}\left(\xi \right)={\left(e-\xi /2\right)}^{-1}\left(e+\xi /2\right)$ which is valid for a general class of quadratic matrix groups (which include the groups SO(3), SE(2), and SE(3)). Based on this simple form, the derivative maps become

$$\begin{array}{cc} d^{L}\mathrm{cay}\left(\xi \right)\cdot \eta & ={\left(e+\xi /2\right)}^{-1}\eta {\left(e-\xi /2\right)}^{-1}\\ d^{L}{\mathrm{cay}}^{-1}\left(\xi \right)\cdot \eta & =\left(e+\xi /2\right)\eta \left(e-\xi /2\right), \end{array}$$

for each ξ,η∈g.

**Example** **17.** *Consider the Lie-Poisson equations with cocycle for* SE(2) *derived in Section 3.3. The central extension integrating the group one-cocycle Equation (70) is* $\hat{SE(2)}=SE\left(2\right)\times R$ *with group two-cocyle* B:SE(2)×SE(2)→R *given by*

$$B\left(\left(\varphi ,a\right),\left(\psi ,b\right)\right)=\frac{1}{2}a\cdot JR_{\varphi }b.$$

*This group is referred to as the oscillator group. To apply the scheme Equation (107) to this case, we use the identities*

$$D_{1}B\left(\left(I,0\right),\left(\varphi ,a\right)\right)=\left(0,\frac{1}{2}Ja\right)\;\mathrm{and}\;D_{2}B\left(\left(\varphi ,a\right),\left(I,0\right)\right)=\left(0,-\frac{1}{2}JR_{\varphi^{-1}}a\right)$$

*as well as the Cayley map for* SE(2) *given by*

$$\mathrm{cay}\left(\lambda ,u_{1},u_{2}\right)=\left(R\left(\lambda \right),\frac{2}{4+\lambda^{2}}\left(-\lambda u_{2}+2u_{1},\lambda u_{1}+2u_{2}\right)\right),\;R\left(\lambda \right)=\frac{1}{4+\lambda^{2}}\left[\begin{array}{cc} \lambda^{2}-4 & -4\lambda \\ 4\lambda & \lambda^{2}-4 \end{array}\right]$$

*and the expression* $d^{L}{\mathrm{cay}}^{-1}\left(\lambda ,u_{1},u_{2}\right):\mathrm{se}\left(2\right)\to \mathrm{se}\left(2\right)$ *given in matrix representation as*

$$I_{3}+\frac{1}{2}\left[\begin{array}{ccc} 0 & 0 & 0\\ u_{2} & 0 & -\lambda \\ -u_{1} & \lambda & 0 \end{array}\right]+\frac{1}{4}\left[\begin{array}{ccc} \lambda^{2} & 0 & 0\\ \lambda u_{1} & 0 & 0\\ \lambda u_{2} & 0 & 0 \end{array}\right]$$

*see [70].*

### 4.4. Multisymplectic Lie Group Variational Integrators

In this paragraph, we briefly indicate how the discrete variational setting of the previous section can be extended to variational discretization in several independent variables, i.e., when the unknown is a field rather than a curve. At the continuous setting, the underlying geometric variational setting is the *multisymplectic framework of field theories*, see, e.g., [48]. Discrete multisymplectic variational versions of this setting were developed and applied in [61,62]. Multisymplectic variational discretization on Lie groups and the discrete Euler-Poincaré field equations were carried out in [63,64].

We will focus on the special case of fields defined on an open subset *U* of $R^{n}$ with smooth boundary, with values in a configuration manifold *Q*. We also assume that the Lagrangian only depends on the values of the fields and their first derivatives, not on the parameter $x\in R^{n}$, so it is a map L:TQ⊕…⊕TQ→R. Hamilton’s principle for a field $q:U\subset R^{n}\to Q$ is

$$\delta \int_{U}L\left(q\left(x\right),\partial_{1}q\left(x\right),\ldots ,\partial_{n}q\left(x\right)\right)dx=0,$$

for arbitrary variations of the field *q* that vanish on the boundary of *U*, from which the Euler-Lagrange equations for the field q(x) are obtained.

We shall focus on the case Q=G a Lie group and for right-invariant Lagrangians, i.e.,

$$L\left(gh,v_{1}h,\ldots ,v_{n}h\right)=L\left(g,v_{1},\ldots ,v_{n}\right),$$

for every $v_{1},\ldots ,v_{n}\in T_{g}G$ and every h∈G. In this case, *L* induces the reduced Lagrangian ℓ:g⊕…⊕g→R defined by $\ell \left(v_{1}g^{-1},\ldots ,v_{n}g^{-1}\right)=L\left(g,v_{1},\ldots ,v_{n}\right)$. As in the ordinary Euler-Poincaré case recalled above, Hamilton’s principle yields the reduced variational principle

(108) $$\delta \int_{U}\ell \left(\xi_{1},\ldots ,\xi_{n}\right)dx=0,\;\delta \xi_{k}=\partial_{k}\eta +\left[\eta ,\xi_{k}\right],$$

for an arbitrary field η:U→g vanishing on the boundary, which results in the *Euler-Poincaré field equations*

(109) $$\sum_{k=1}^{n}\partial_{k}\frac{\delta \ell }{\delta \xi_{k}}+\sum_{k=1}^{n}{\mathrm{ad}}_{\xi_{k}}^{*}\frac{\delta \ell }{\delta \xi_{k}}=0.$$

To guarantee the existence of a field g:U→G such that $\xi_{k}=\partial_{k}gg^{-1}$, k=1,…,n, the fields $\xi_{i}$ in Equation (109) must satisfy the relation $\partial_{k}\xi_{i}-\partial_{i}\xi_{k}=\left[\xi_{k},\xi_{i}\right]$. In terms of the associated Hamiltonian $h:g^{*}⊕\ldots ⊕g^{*}\to R$, these equations give Equation (54) without cocycle, i.e., with $\Theta^{k}=0$.

To include the case with cocycle in a variational setting, we shall proceed exactly as in Section 4.2, by passing to a central extension of *G*. This is here done in the context of the Euler-Poincaré field equations, rather than for the ordinary Euler-Poincaré equations. This is the content of the next paragraph.

**Variational principle for the Lie-Poisson field equations with cocycle.** The goal of this paragraph is to obtain a variational principle for the Lie-Poisson field equations with cocycle Equation (54) associated with Souriau’s polysymplectic model. By considering the Euler-Poincaré field equations Equation (109) on a central extension, we get the system

(110) $$\left\{\begin{array}{c} \sum_{k}\partial_{k}\frac{\delta \hat{\ell }}{\delta \xi_{k}}+\sum_{k}{\mathrm{ad}}_{\xi_{k}}^{*}\frac{\delta \hat{\ell }}{\delta \xi_{k}}=\sum_{k}\frac{\delta \hat{\ell }}{\delta u_{k}}\Theta \left(\xi_{k},\cdot \right)\\ \sum_{k}\partial_{k}\frac{\delta \hat{\ell }}{\delta u_{k}}=0. \end{array}\right.$$

They are the critical conditions for the variational principle

(111) $$\begin{array}{cc} \; & \delta \int_{0}^{T}\hat{\ell }\left(\left(\xi_{1},u_{1}\right),\ldots ,\left(\xi_{n},u_{n}\right)\right)dt=0,\\ \; & \mathrm{for}\;\mathrm{variations}\;\delta \xi_{k}=\partial_{k}\eta +\left[\eta ,\xi_{k}\right],\;\delta u_{k}=\partial_{k}v-\Theta \left(\eta ,\xi_{k}\right), \end{array}$$

which is just a special instance of Equation (109) applied to central extensions. The existence of a field $\left(g,\alpha \right):U\to \hat{G}$ imposes the conditions $\partial_{k}\xi_{i}-\partial_{i}\xi_{k}=\left[\xi_{k},\xi_{i}\right]$ and $\partial_{k}u_{i}-\partial_{i}u_{k}=-\Theta \left(\xi_{k},\xi_{i}\right)$.

Given a Lagrangian ℓ:g⊕…⊕g→R, one can define the Lagrangian

(112) $$\hat{\ell }\left(\left(\xi_{1},u_{1}\right),\ldots ,\left(\xi_{n},u_{n}\right)\right)=\ell \left(\xi_{1},\ldots ,\xi_{n}\right)+\sum_{k}u_{k}$$

on $\hat{g}⊕\ldots ⊕\hat{g}$ for which Equation (110) does reduce to a Lagrangian version of the Lie-Poisson field equation with cocycle Equation (54), as desired, where it is assumed that $\Theta^{k}=\Theta$, for all *k*.

The same reasoning also directly applies on the Hamiltonian side, in which case the Lie-Poisson field equation with cocycle Equation (89) is an invariant subsystem of an ordinary Lie-Poisson field equation associated with a central extension of *G*.

**Remark** **18** (Polysymplectic vs multisymplectic setting). The field theories that are used in this paper can be described within the restricted setting of *polysymplectic geometry*. For such particular field theories the configuration bundle of the theory is trivial, the base is Euclidean, and the Lagrangian does not depend on the variables in the base. General classical field theories cannot be described by polysymplectic geometry and fit into the more general setting of *multisymplectic geometry*. This mainly comes from the fact that the field theoretic analogue to the cotangent bundle (endowed with the canonical symplecic form) of mechanics is the dual jet bundle of the configuration bundle (endowed with a canonical multisymplectic form). We shall apply below multisymplectic variational integrators to field equations belonging to the setting of polysysmplectic geometry. In this case, they could logically be called polysymplectic variational integrators. Please note that we kept the original naming multisymplectic variational integrators, since the theory applies to general multisymplectic field theories not just polysymplectic ones. See, e.g., [62,64] for applications of multisymplectic integrators to situations that are not covered by the polysymplectic formalism.

**Multisymplectic Lie group integrators.** To present multisymplectic integrators, we shall focus on the two dimensional case and assume that the fields are defined on a rectangle $U=\left[0,A\right]\times \left[0,B\right]\subset R^{2}$. We shall write $\left(x_{1},x_{2}\right)=\left(x,y\right)$. Let *Q* be a configuration manifold and let L:TQ⊕TQ→R be a Lagrangian. We shall consider the very special case of a discrete grid determined by $\left\{\left(x_{k},y_{a}\right)=\left(k\Delta x,a\Delta y\right)|k=0,\ldots ,N_{1},\;a=1,\ldots ,N_{2}\right\}$ with given Δx and Δy. We shall denote by $q_{d}:{\left\{\left(x_{k},y_{a}\right)\right\}}_{k=0}^{N}\to Q$, $q_{d}\left(x_{k},y_{a}\right)=q_{k}^{a}$ a discrete field. A *discrete Lagrangian* is a map $L_{d}:Q\times Q\times Q\to R$, $L_{d}=L_{d}\left(q_{k}^{a},q_{k+1}^{a},q_{a+1}^{k}\right)$ that approximates the action integral of *L* on the rectangle $\left[x_{k},x_{k+1}\right]\times \left[y_{a},y_{a+1}\right]$ for a field interpolating the values $q_{k}^{a},q_{k+1}^{a},q_{a+1}^{k}$. The discrete Hamilton principle reads

(113) $$\delta \sum_{k=0}^{N_{1}-1}\sum_{a=0}^{N_{2}-1}L_{d}\left(q_{k}^{a},q_{k+1}^{a},q_{a+1}^{k}\right)=0,$$

for all variations $\delta q_{d}$ of $q_{d}$ with vanishing boundary values. The discrete Euler-Lagrange equations are obtained as the critical point condition for a discrete field $q_{d}$.

Given a Lie group action Φ:G×Q→Q, the *discrete Lagrangian field momentum maps* $J_{L_{d}}^{i},:Q\times Q\times Q\to g^{*}$, i=1,2,3 are defined by

(114) $$\begin{array}{cc} \langle J_{L_{d}}^{1}\left(q_{k}^{a},q_{k+1}^{a},q_{k}^{a+1}\right),\xi \rangle & =\langle D_{1}L_{d}\left(q_{k}^{a},q_{k+1}^{a},q_{k}^{a+1}\right),\xi_{Q}\left(q_{k}^{a}\right)\rangle \\ \langle J_{L_{d}}^{2}\left(q_{k}^{a},q_{k+1}^{a},q_{k}^{a+1}\right),\xi \rangle & =\langle D_{2}L_{d}\left(q_{k}^{a},q_{k+1}^{a},q_{k}^{a+1}\right),\xi_{Q}\left(q_{k+1}^{a}\right)\rangle \\ \langle J_{L_{d}}^{3}\left(q_{k}^{a},q_{k+1}^{a},q_{k}^{a+1}\right),\xi \rangle & =\langle D_{3}L_{d}\left(q_{k}^{a},q_{k+1}^{a},q_{k}^{a+1}\right),\xi_{Q}\left(q_{k}^{a+1}\right)\rangle \end{array}$$

which satisfies $J_{L_{d}}^{1}+J_{L_{d}}^{2}+J_{L_{d}}^{3}=0$.

We refer to [61,62] for an introduction to multisymplectic variational integrators, including the notion of discrete multisymplecticity, discrete Cartan forms, and discrete field momentum maps, see also [71]. These integrators, also satisfy a discrete Noether theorem in presence of symmetries, as we shall see below in the special case of Lie groups.

Multisymplectic variational integrators on Lie groups were developed in [63,71], for application to geometrically exact (Cosserat) rods. As above, we shall focus on the two dimensional case and $U=\left[0,A\right]\times \left[0,B\right]\subset R^{2}$. For Q=G a Lie group, the discrete Lagrangian is a map $L_{d}:G\times G\times G\to R$. We assume that the continuous Lagrangian is *G* invariant and that the discrete Lagrangian $L_{d}$ inherits this invariance, i.e.,

$$L\left(g_{k}^{a}h,g_{k+1}^{a}h,g_{a+1}^{k}h\right)=L\left(g_{k}^{a},g_{k+1}^{a},g_{a+1}^{k}\right),$$

for every h∈G. Hence, by passing to the quotient associated with this action we get a reduced Lagrangian $L_{d}:G\times G\to R$, $L_{d}\left(g_{k+1}^{a}{\left(g_{k}^{a}\right)}^{-1},g_{k}^{a+1}{\left(g_{k}^{a}\right)}^{-1}\right)=L\left(g_{k}^{a},g_{k+1}^{a},g_{a+1}^{k}\right)$. As mentioned earlier, it is advantageous to introduce a retraction map τ:g→G, τ(0)=e, from which the discrete reduced Lagrangian can be defined on a neighborhood of (0,0) in g×g via the relation

(115) $$\begin{array}{cc} \; & \ell_{g}\left(\xi_{k}^{a},\zeta_{k}^{a}\right)=L_{d}\left(g_{k+1}^{a}{\left(g_{k}^{a}\right)}^{-1},g_{k}^{a+1}{\left(g_{k}^{a}\right)}^{-1}\right)\\ \; & \mathrm{with}\;\tau \left(\Delta x\xi_{k}^{a}\right)=g_{k+1}^{a}{\left(g_{k}^{a}\right)}^{-1}\;\mathrm{and}\;\tau \left(\Delta y\zeta_{k}^{a}\right)=g_{k}^{a+1}{\left(g_{k}^{a}\right)}^{-1}. \end{array}$$

The last two relations are thought of as discrete versions of $\xi =\partial_{1}gg^{-1}$, $\zeta =\partial_{2}gg^{-1}$.

The discrete Euler-Poincaré field equations for $\ell_{d}$ are obtained by computing the discrete variational principle induced on the discrete action $\sum_{k=0}^{N_{1}-1}\sum_{a=0}^{N_{2}-1}\ell_{d}\left(\xi_{k}^{a},\zeta_{k}^{a}\right)$ from the discrete Hamilton principle $\delta \sum_{k=0}^{N_{1}-1}\sum_{a=0}^{N_{2}-1}L\left(g_{k}^{a},g_{k+1}^{a},g_{k}^{a+1}\right)=0$ recalled above in Equation (113). The main step in this process is to compute the variations $\delta \xi_{k}^{a}$$\delta \zeta_{k}^{a}$ induced by arbitrary variations $\delta g_{k}^{a}$. One finds the expression

(116) $$\begin{array}{cc} \delta \xi_{k}^{a} & =\frac{1}{\Delta x}d^{L}\tau^{-1}\left(\Delta x\xi_{k}^{a}\right)\cdot \left({\mathrm{Ad}}_{\tau {\left(\Delta x\xi_{k}^{a}\right)}^{-1}}\eta_{k+1}^{a}-\eta_{k}^{a}\right)\\ \delta \zeta_{k}^{a} & =\frac{1}{\Delta y}d^{L}\tau^{-1}\left(\Delta y\zeta_{k}^{a}\right)\cdot \left({\mathrm{Ad}}_{\tau {\left(\Delta y\zeta_{k}^{a}\right)}^{-1}}\eta_{k}^{a+1}-\eta_{k}^{a}\right). \end{array}$$

The *discrete field Euler-Poincaré variational principle* thus reads

(117) $$\delta \sum_{k=0}^{N_{1}-1}\sum_{a=0}^{N_{2}-1}\ell_{d}\left(\xi_{k}^{a},\zeta_{k}^{a}\right)=0,$$

with respect to variations $\delta \xi_{k}^{a}$, $\delta \zeta_{k}^{a}$ of the form Equation (116) with $\eta_{k}^{a}$ vanishing at the boundary. It yields the *discrete Euler-Poincaré field equations*.

(118) $$\begin{array}{cc} \; & \frac{1}{\Delta x}\left({\mathrm{Ad}}_{\tau {\left(\Delta x\xi_{k-1}^{a}\right)}^{-1}}^{*}\mu_{k-1}^{a}-\mu_{k}^{a}\right)+\frac{1}{\Delta y}\left({\mathrm{Ad}}_{\tau {\left(\Delta y\zeta_{k}^{a-1}\right)}^{-1}}^{*}\nu_{k}^{a-1}-\nu_{k}^{a}\right)=0,\\ \; & \mu_{k}^{a}:=d^{L}\tau^{-1}{\left(\Delta x\xi_{k}^{a}\right)}^{*}\frac{\delta \ell }{\delta \xi_{k}^{a}},\;\nu_{k}^{a}:=d^{L}\tau^{-1}{\left(\Delta y\zeta_{k}^{a}\right)}^{*}\frac{\delta \ell }{\delta \zeta_{k}^{a}}. \end{array}$$

We refer to [63,71] for details, including the treatment of boundary conditions, the description of the associated discrete Cartan forms, the discrete field momentum maps, as well as the symplectic and multisymplectic characters of the scheme.

We just recall below the expression of the field momentum maps Equation (114) which take the following form:

$$\begin{array}{cc} J_{L_{d}}^{1}\left(g_{k}^{a},g_{k+1}^{a},g_{k}^{a+1}\right) & =-\frac{1}{\Delta x}{\mathrm{Ad}}_{g_{k}}^{*}\mu_{k}^{a}-\frac{1}{\Delta y}{\mathrm{Ad}}_{g_{k}}^{*}\nu_{k}^{a}\\ J_{L_{d}}^{2}\left(g_{k}^{a},g_{k+1}^{a},g_{k}^{a+1}\right) & =\frac{1}{\Delta x}{\mathrm{Ad}}_{g_{k}}^{*}\mu_{k}^{a}\\ J_{L_{d}}^{3}\left(g_{k}^{a},g_{k+1}^{a},g_{k}^{a+1}\right) & =\frac{1}{\Delta y}{\mathrm{Ad}}_{g_{k}}^{*}\nu_{k}^{a}. \end{array}$$

The *discrete Noether theorem*, then asserts that a certain $g^{*}$-valued discrete integral of $J_{L_{d}}^{i}$ along the boundary of any subgrid domain is zero, see [71].

**Multisymplectic variational discretization for Lie-Poisson field equations with cocycle.** Based on the previous result, we first give below a multisymplectic integrator for the Euler-Poincaré field equations on central extensions. Then we deduce a multisymplectic integrator for the Lie-Poisson field equations with cocycle appearing in the polysymplectic Souriau model. The next proposition is the multisymplectic version of Proposition 15.

**Proposition 19.**  (Discrete Euler-Poincaré field equations on central extensions) *The following are equivalent:*

(a) *The discrete curve*$\left(\xi_{k},u_{k}\right)$*is critical for the discrete Euler-Poincaré field variational principle*
  $$\delta \sum_{a,k}\hat{\ell }\left(\left(\xi_{k}^{a},u_{k}^{a}\right),\left(\zeta_{k}^{a},w_{k}^{a}\right)\right)=0,$$
  *with respect to variations*
  $$\left\{\begin{array}{c} \delta \xi_{k}^{a}=\frac{1}{\Delta x}d^{L}{\bar{\tau }}^{-1}\left(\Delta x\xi_{k}^{a}\right)\cdot \left({\mathrm{Ad}}_{\tau {\left(\Delta x\xi_{k}^{a}\right)}^{-1}}\eta_{k+1}^{a}-\eta_{k}^{a}\right)\\ \delta \zeta_{k}^{a}=\frac{1}{\Delta y}d^{L}\tau^{-1}\left(\Delta y\zeta_{k}^{a}\right)\cdot \left({\mathrm{Ad}}_{\tau {\left(\Delta y\zeta_{k}^{a}\right)}^{-1}}\eta_{k}^{a+1}-\eta_{k}^{a}\right)\\ \delta u_{k}^{a}=\frac{1}{\Delta x}\left(v_{k+1}^{a}-v_{k}^{a}-D_{2}B\left(\bar{\tau }\left(\Delta x\xi_{k}^{a}\right),e\right)\cdot \eta_{k}^{a}+D_{1}B\left(e,\bar{\tau }\left(\Delta x\xi_{k}^{a}\right)\right)\cdot \eta_{k+1}^{a}\right)\\ \delta w_{k}^{a}=\frac{1}{\Delta y}\left(v_{k}^{a+1}-v_{k}^{a}-D_{2}B\left(\bar{\tau }\left(\Delta y\zeta_{k}^{a}\right),e\right)\cdot \eta_{k}^{a}+D_{1}B\left(e,\bar{\tau }\left(\Delta y\zeta_{k}^{a}\right)\right)\cdot \eta_{k}^{a+1}\right) \end{array}\right.$$
  *where*$\eta_{k}^{a}\in g$ and $v_{k}^{a}\in R$ *are arbitrary discrete fields vanishing at the boundary.*
(b) *The discrete curve* $\left(\xi_{k}^{a},u_{k}^{a},\zeta_{k}^{a},w_{k}^{a}\right)$ *is a solution of the discrete Euler-Poincaré field equations*
  (119) $$\left\{\begin{array}{c} \frac{1}{\Delta x}\left({\mathrm{Ad}}_{\tau {\left(\Delta x\xi_{k-1}^{a}\right)}^{-1}}^{*}\mu_{k-1}^{a}+a_{k-1}^{a}\theta \left(\bar{\tau }\left(\Delta x\xi_{k-1}^{a}\right)\right)-\mu_{k}^{a}\right)\\ \;\;+\frac{1}{\Delta y}\left({\mathrm{Ad}}_{\tau {\left(\Delta y\zeta_{k}^{a-1}\right)}^{-1}}^{*}\nu_{k}^{a-1}+b_{k}^{a-1}\theta \left(\bar{\tau }\left(\Delta y\zeta_{k}^{a-1}\right)\right)-\nu_{k}^{a}\right)=0\\ \frac{1}{\Delta x}\left(a_{k-1}^{a}-a_{k}^{a}\right)+\frac{1}{\Delta y}\left(b_{k}^{a-1}-b_{k}^{a}\right)=0 \end{array}\right.$$
  *with*
  (120) $$\left\{\begin{array}{c} \mu_{k}^{a}=d^{L}{\bar{\tau }}^{-1}{\left(\Delta x\xi_{k}^{a}\right)}^{*}\frac{\delta \hat{\ell }}{\delta \xi_{k}^{a}}+\frac{\delta \hat{\ell }}{\delta u_{k}^{a}}D_{2}B\left(\bar{\tau }\left(\Delta x\xi_{k}^{a}\right),e\right)\\ \nu_{k}^{a}=d^{L}{\bar{\tau }}^{-1}{\left(\Delta y\zeta_{k}^{a}\right)}^{*}\frac{\delta \hat{\ell }}{\delta \zeta_{k}^{a}}+\frac{\delta \hat{\ell }}{\delta w_{k}^{a}}D_{2}B\left(\bar{\tau }\left(\Delta y\zeta_{k}^{a}\right),e\right)\\ a_{k}^{a}=\frac{\delta \hat{\ell }}{\delta u_{k}^{a}},\;b_{k}^{a}=\frac{\delta \hat{\ell }}{\delta w_{k}^{a}}. \end{array}\right.$$

**Proof.**  The proof can be obtained by appropriate extension of the proof of Proposition 15, by using the multisymplectic variational setting recalled in the previous paragraph. ☐

We note that the relation between the solution of the discrete Euler-Poincaré equations and the solution $\left(g_{k}^{a},\alpha_{k}^{a}\right)$ of the discrete Euler-Lagrange field equations on the Lie group $\hat{G}$ is given as

$$\begin{array}{cc} \left(\xi_{k}^{a},u_{k}^{a}\right) & =\frac{1}{\Delta x}\tau^{-1}\left(\left(g_{k+1}^{a},\alpha_{k+1}^{a}\right){\left(g_{k}^{a},\alpha_{k}^{a}\right)}^{-1}\right)\\ \left(\zeta_{k}^{a},w_{k}^{a}\right) & =\frac{1}{\Delta y}\tau^{-1}\left(\left(g_{k}^{a+1},\alpha_{k}^{a+1}\right){\left(g_{k}^{a},\alpha_{k}^{a}\right)}^{-1}\right) \end{array}$$

which is explicitly given by the relations

(121) $$\begin{array}{cc} \xi_{k}^{a} & =\frac{1}{\Delta x}\tau^{-1}\left(g_{k+1}^{a}{\left(g_{k}^{a}\right)}^{-1}\right),\;\zeta_{k}=\frac{1}{\Delta y}\tau^{-1}\left(g_{k}^{a+1}{\left(g_{k}^{a}\right)}^{-1}\right)\\ u_{k}^{a} & =\frac{1}{\Delta x}\left(\alpha_{k+1}^{a}-\alpha_{k}^{a}-B\left(g_{k}^{a},{\left(g_{k}^{a}\right)}^{-1}\right)+B\left(g_{k+1}^{a},{\left(g_{k}^{a}\right)}^{-1}\right)\right)\\ w_{k}^{a} & =\frac{1}{\Delta y}\left(\alpha_{k}^{a+1}-\alpha_{k}^{a}-B\left(g_{k}^{a},{\left(g_{k}^{a}\right)}^{-1}\right)+B\left(g_{k}^{a+1},{\left(g_{k}^{a}\right)}^{-1}\right)\right). \end{array}$$

Similarly, the variations $\eta_{k}^{a},v_{k}^{a}$ used in discrete Euler-Poincaré variational principle are related to the variations $\delta g_{k}^{a}$, $\delta \alpha_{k}^{a}$ used in the discrete Hamilton principle via the equality $\left(\eta_{k}^{a},v_{k}^{a}\right)=\left(\delta g_{k}^{a},\delta \alpha_{k}^{a}\right){\left(g_{k}^{a},\alpha_{k}^{a}\right)}^{-1}=\left(\delta g_{k}^{a}{\left(g_{k}^{a}\right)}^{-1},\delta \alpha_{k}^{a}+D_{1}B\left(g_{k}^{a},{\left(g_{k}^{a}\right)}^{-1}\right)\cdot \delta g_{k}^{a}\right)$.

The discrete field momentum maps are computed as

$$\begin{array}{cc} J_{L_{d}}^{1}\left(\left(g_{k}^{a},\alpha_{k}^{a}\right),\left(g_{k+1}^{a},\alpha_{k+1}^{a}\right),\left(g_{k}^{a+1},\alpha_{k}^{a+1}\right)\right) & =-\frac{1}{\Delta x}\left({\mathrm{Ad}}_{g_{k}^{a}}^{*}\mu_{k}^{a}+a_{k}^{a}\theta \left({\left(g_{k}^{a}\right)}^{-1}\right),a_{k}^{a}\right)\\ \; & \;-\frac{1}{\Delta y}\left({\mathrm{Ad}}_{g_{k}^{a}}^{*}\nu_{k}^{a}+b_{k}^{a}\theta \left({\left(g_{k}^{a}\right)}^{-1}\right),b_{k}^{a}\right)\\ J_{L_{d}}^{2}\left(\left(g_{k}^{a},\alpha_{k}^{a}\right),\left(g_{k+1}^{a},\alpha_{k+1}^{a}\right),\left(g_{k}^{a+1},\alpha_{k}^{a+1}\right)\right) & =\frac{1}{\Delta x}\left({\mathrm{Ad}}_{g_{k}^{a}}^{*}\mu_{k}^{a}+a_{k}^{a}\theta \left({\left(g_{k}^{a}\right)}^{-1}\right),a_{k}^{a}\right)\\ J_{L_{d}}^{3}\left(\left(g_{k}^{a},\alpha_{k}^{a}\right),\left(g_{k+1}^{a},\alpha_{k+1}^{a}\right),\left(g_{k}^{a+1},\alpha_{k}^{a+1}\right)\right) & =\frac{1}{\Delta y}{\mathrm{Ad}}_{g_{k}}^{*}\left({\mathrm{Ad}}_{g_{k}^{a}}^{*}\nu_{k}^{a}+b_{k}^{a}\theta \left({\left(g_{k}^{a}\right)}^{-1}\right),b_{k}^{a}\right) \end{array}$$

from which the discrete field Noether theorem can be stated for the solutions of Equation (119).

The multisymplectic integrator for the Lie-Poisson field equations with cocycle is obtained in the next Proposition, which is the multisymplectic analogue to Proposition 16.

**Proposition 20.**  (Multisymplectic integrator for Lie-Poisson field equations with cocycle) *Let* $h:g^{*}\times g^{*}\to R$ *be a Hamiltonian assumed to be hyperregular, with associated Lagrangian* ℓ:g×g→R*. Then the numerical scheme*

(122) $$\begin{array}{cc} \; & \frac{1}{\Delta x}\left({\mathrm{Ad}}_{\tau {\left(\Delta x\xi_{k-1}^{a}\right)}^{-1}}^{*}\mu_{k-1}^{a}+\theta \left(\bar{\tau }\left(\Delta x\xi_{k-1}^{a}\right)\right)-\mu_{k}^{a}\right)\\ \; & \;\;+\frac{1}{\Delta y}\left({\mathrm{Ad}}_{\tau {\left(\Delta y\zeta_{k}^{a-1}\right)}^{-1}}^{*}\nu_{k}^{a-1}+\theta \left(\bar{\tau }\left(\Delta y\zeta_{k}^{a-1}\right)\right)-\nu_{k}^{a}\right)=0 \end{array}$$

*with*

(123) $$\left\{\begin{array}{c} \;\mu_{k}^{a}=d^{L}{\bar{\tau }}^{-1}{\left(\Delta x\xi_{k}^{a}\right)}^{*}\frac{\delta \ell }{\delta \xi_{k}^{a}}+D_{2}B\left(\bar{\tau }\left(\Delta x\xi_{k}^{a}\right),e\right)\\ \;\nu_{k}^{a}=d^{L}{\bar{\tau }}^{-1}{\left(\Delta y\zeta_{k}^{a}\right)}^{*}\frac{\delta \ell }{\delta \zeta_{k}^{a}}+D_{2}B\left(\bar{\tau }\left(\Delta y\zeta_{k}^{a}\right),e\right) \end{array}\right.$$

*is a multisymplectic scheme for the Lie-Poisson field equations with cocycle*

(124) $$\partial_{x}\mu +\partial_{y}\nu +{\mathrm{ad}}_{\frac{\delta h}{\delta \mu }}^{*}\mu +{\mathrm{ad}}_{\frac{\delta h}{\delta \nu }}^{*}\nu =\Theta \left(\frac{\delta h}{\delta \mu },\cdot \right)+\Theta \left(\frac{\delta h}{\delta \nu },\cdot \right).$$

**Proof.**  This follows from Proposition 19 and the choice Equation (112). ☐

If the retraction map τ satisfies τ(−ξ)τ(ξ)=e, the scheme can be rewritten in a simpler way, as done in Equation (107) in the symplectic case.

The benefit of the structure preserving properties of the proposed numerical schemes will be exploited in a future work.

## 5. Conclusions

In the context of artificial intelligence, machine learning algorithms use more and more methodological tools coming from physics or statistical mechanics. The laws and principles that underpin this physics can shed new light on the conceptual basis of artificial intelligence. Thus, the principles of maximum entropy and François Massieu’s notions of characteristic functions enrich the variational formalism of machine learning. Conversely, the pitfalls encountered by artificial intelligence to extend its application domains, question the foundations of statistical physics, such as the generalization of the notions of Gibbs densities in spaces of more elaborate representation such as data on homogeneous symplectic manifolds and Lie groups. The porosity between the two disciplines has been established since the birth of artificial intelligence with the use of Boltzmann machines and the problem of robust methods for calculating partition function. More recently, gradient algorithms for neural network learning use large-scale robust extensions of the natural gradient of Fisher-based information geometry (to ensure reparameterization invariance), and stochastic gradient based on the Langevin equation (to ensure regularization), or their coupling called “Natural Langevin Dynamics”. Concomitantly, during the last fifty years, statistical physics has been the object of new geometrical formalizations (contact, Dirac, or symplectic geometry, variational principles, etc.) to try to give a new covariant formalization to the thermodynamics of dynamical systems, as Lie Groups thermodynamics. Finally, the study of geometric integrators as symplectic integrators with good properties of covariances and stability (use of symmetries, preservation of invariants and momentum maps) will open the door to new generation of numerical schemes. Machine learning inference processes are just beginning to adapt these new integration schemes and their remarkable stability properties to increasingly abstract data representation spaces. Artificial intelligence currently uses only a very limited portion of the conceptual and methodological tools of statistical physics. The purpose of this paper was to encourage constructive dialogue around a common foundation, to allow the establishment of new principles and laws governing the two disciplines in a unified approach.
